# Supplementary material for: Machine Learning Based Multi-Parameter Modeling for Prediction of Post-Inflammatory Lung Changes
Source: Diagnostics (Basel). 2025 Mar 20;15(6):783. doi: 10.3390/diagnostics15060783 (PMC11941013; doi:10.3390/diagnostics15060783)
Supplement: Supplementary file 1 [file diagnostics-15-00783-s001.zip › figure_s10_tuning_regression_dlco.pdf]

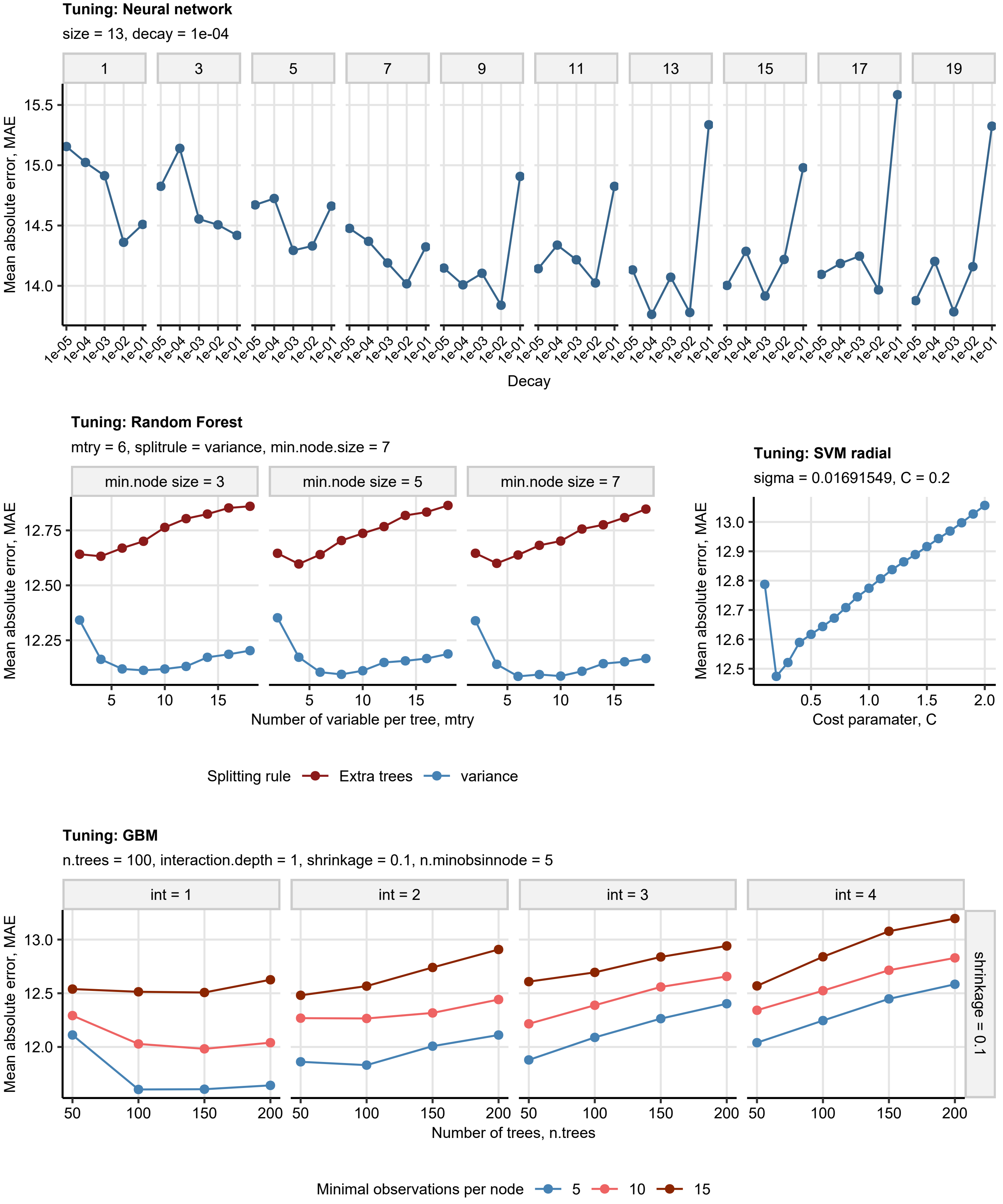
Tuning: Neural network
size = 13, decay = 1e-04
1
3
5
7
9
11
13
15
17
19
Mean absolute error, MAE
15.5
15.0
14.5
14.0
1e-05
1e-04
1e-03
1e-02
1e-01
Decay
Tuning: Random Forest
mtry = 6, splitrule = variance, min.node.size = 7
min.node size = 3
min.node size = 5
min.node size = 7
Mean absolute error, MAE
12.75
12.50
12.25
5
10
15
Number of variable per tree, mtry
Tuning: SVM radial
sigma = 0.01691549, C = 0.2
Mean absolute error, MAE
13.0
12.9
12.8
12.7
12.6
12.5
0.5
1.0
1.5
2.0
Cost paramater, C
Splitting rule
Extra trees
variance
Tuning: GBM
n.trees = 100, interaction.depth = 1, shrinkage = 0.1, n.minobsinnode = 5
int = 1
int = 2
int = 3
int = 4
shrinkage = 0.1
Mean absolute error, MAE
13.0
12.5
12.0
50
100
150
200
Number of trees, n.trees
Minimal observations per node
5
10
15
