# Supplementary material for: Machine Learning Based Multi-Parameter Modeling for Prediction of Post-Inflammatory Lung Changes
Source: Diagnostics (Basel). 2025 Mar 20;15(6):783. doi: 10.3390/diagnostics15060783 (PMC11941013; doi:10.3390/diagnostics15060783)
Supplement: Supplementary file 1 [file diagnostics-15-00783-s001.zip › figure_s11_performance_fvc_fev_findings_models.pdf]

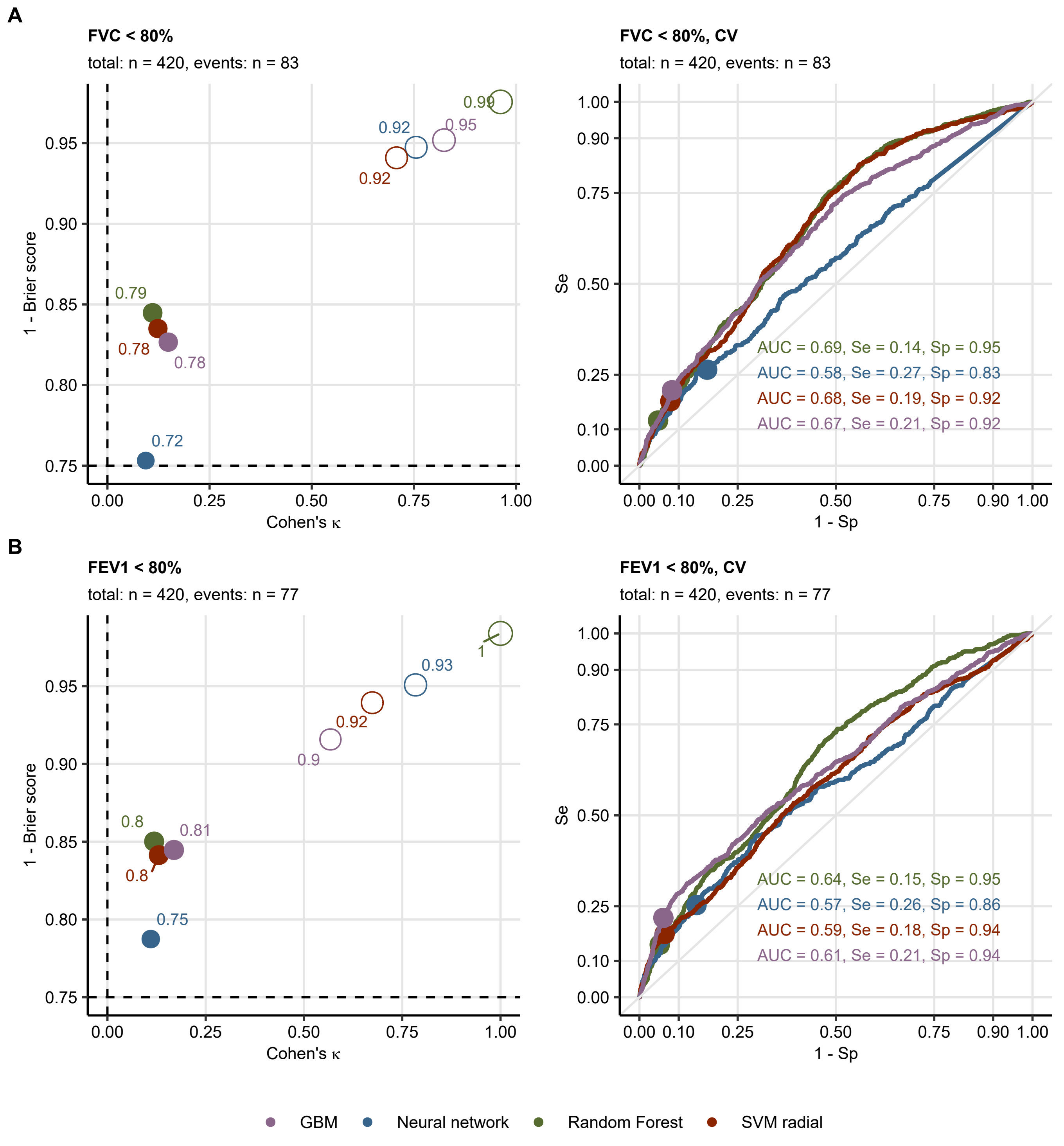
A
FVC < 80%
total: n = 420, events: n = 83
1 - Brier score
Cohen's κ
FVC < 80%, CV
total: n = 420, events: n = 83
Se
1 - Sp
AUC = 0.69, Se = 0.14, Sp = 0.95
AUC = 0.58, Se = 0.27, Sp = 0.83
AUC = 0.68, Se = 0.19, Sp = 0.92
AUC = 0.67, Se = 0.21, Sp = 0.92
B
FEV1 < 80%
total: n = 420, events: n = 77
1 - Brier score
Cohen's κ
FEV1 < 80%, CV
total: n = 420, events: n = 77
Se
1 - Sp
AUC = 0.64, Se = 0.15, Sp = 0.95
AUC = 0.57, Se = 0.26, Sp = 0.86
AUC = 0.59, Se = 0.18, Sp = 0.94
AUC = 0.61, Se = 0.21, Sp = 0.94
GBM
Neural network
Random Forest
SVM radial
