# Supplementary material for: Machine Learning Based Multi-Parameter Modeling for Prediction of Post-Inflammatory Lung Changes
Source: Diagnostics (Basel). 2025 Mar 20;15(6):783. doi: 10.3390/diagnostics15060783 (PMC11941013; doi:10.3390/diagnostics15060783)
Supplement: Supplementary file 1 [file diagnostics-15-00783-s001.zip › figure_s13_model_errors_severity_follow_up.pdf]

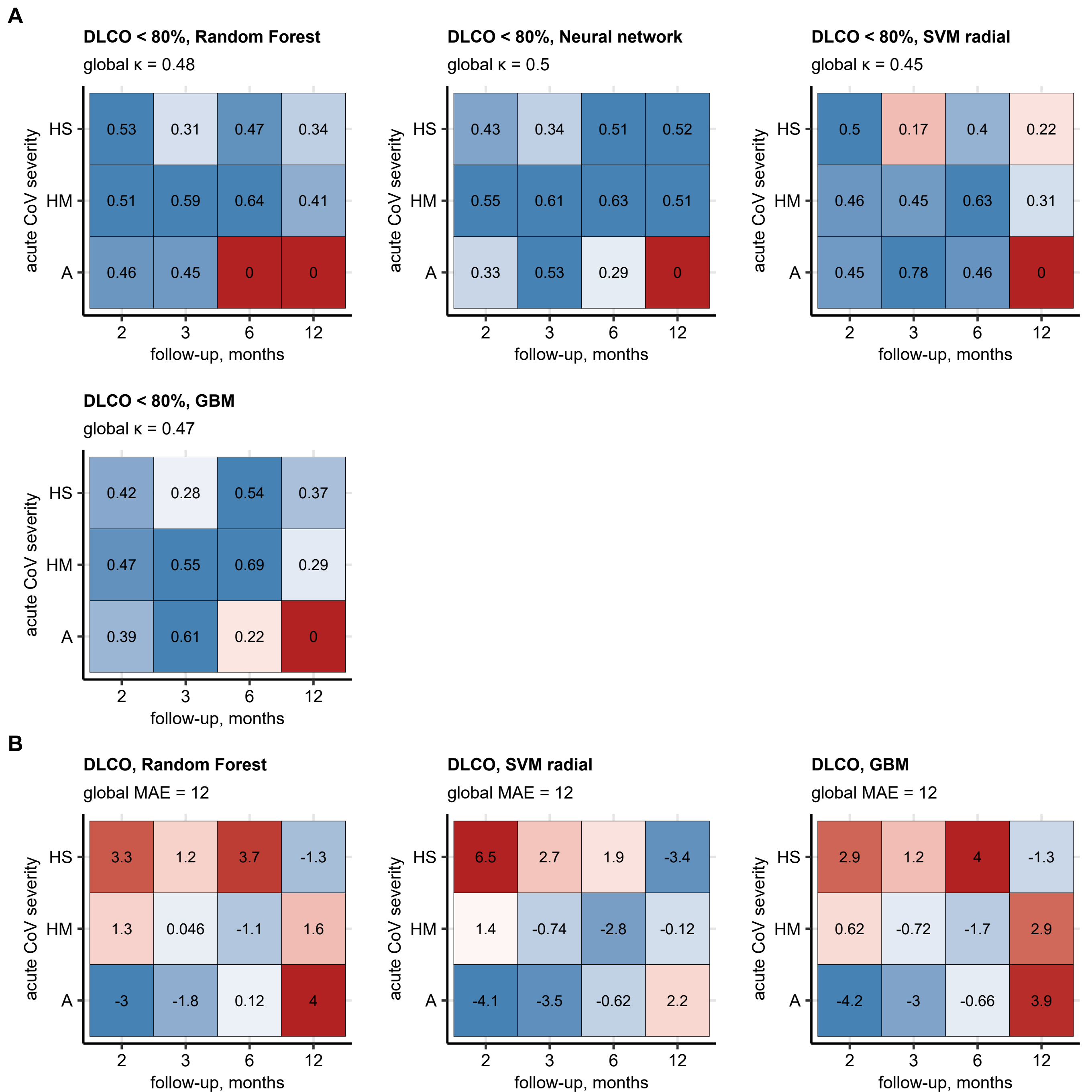
A
DLCO < 80%, Random Forest
global κ = 0.48
HS: 0.53, 0.31, 0.47, 0.34
HM: 0.51, 0.59, 0.64, 0.41
A: 0.46, 0.45, 0, 0
DLCO < 80%, Neural network
global κ = 0.5
HS: 0.43, 0.34, 0.51, 0.52
HM: 0.55, 0.61, 0.63, 0.51
A: 0.33, 0.53, 0.29, 0
DLCO < 80%, SVM radial
global κ = 0.45
HS: 0.5, 0.17, 0.4, 0.22
HM: 0.46, 0.45, 0.63, 0.31
A: 0.45, 0.78, 0.46, 0
DLCO < 80%, GBM
global κ = 0.47
HS: 0.42, 0.28, 0.54, 0.37
HM: 0.47, 0.55, 0.69, 0.29
A: 0.39, 0.61, 0.22, 0
B
DLCO, Random Forest
global MAE = 12
HS: 3.3, 1.2, 3.7, -1.3
HM: 1.3, 0.046, -1.1, 1.6
A: -3, -1.8, 0.12, 4
DLCO, SVM radial
global MAE = 12
HS: 6.5, 2.7, 1.9, -3.4
HM: 1.4, -0.74, -2.8, -0.12
A: -4.1, -3.5, -0.62, 2.2
DLCO, GBM
global MAE = 12
HS: 2.9, 1.2, 4, -1.3
HM: 0.62, -0.72, -1.7, 2.9
A: -4.2, -3, -0.66, 3.9
acute CoV severity
follow-up, months
2
3
6
12
