# Supplementary material for: Machine Learning Based Multi-Parameter Modeling for Prediction of Post-Inflammatory Lung Changes
Source: Diagnostics (Basel). 2025 Mar 20;15(6):783. doi: 10.3390/diagnostics15060783 (PMC11941013; doi:10.3390/diagnostics15060783)
Supplement: Supplementary file 1 [file diagnostics-15-00783-s001.zip › figure_s17_top_variables_co_linearity.pdf]

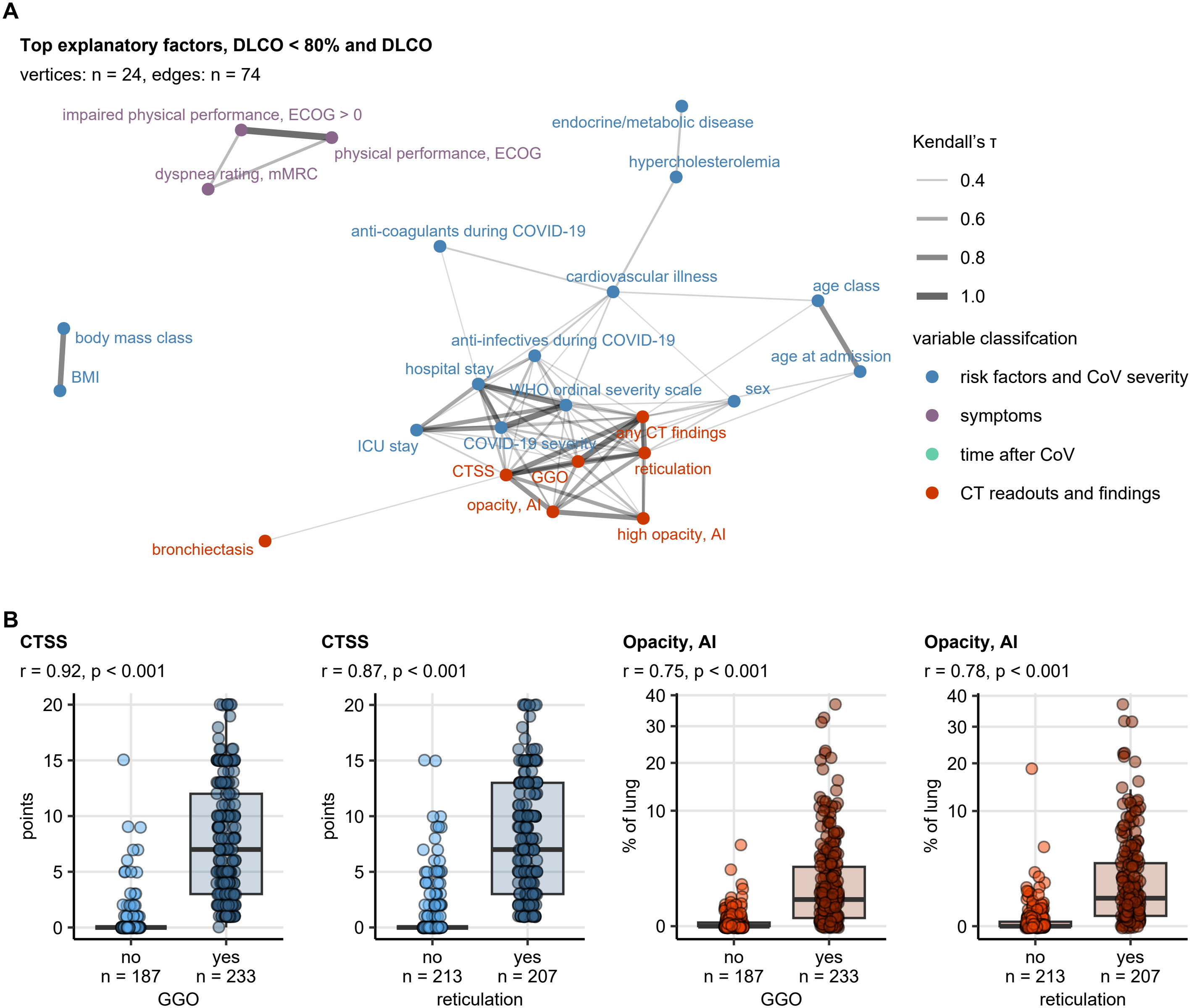
A
Top explanatory factors, DLCO < 80% and DLCO
vertices: n = 24, edges: n = 74
impaired physical performance, ECOG > 0
physical performance, ECOG
dyspnea rating, mMRC
endocrine/metabolic disease
hypercholesterolemia
anti-coagulants during COVID-19
cardiovascular illness
age class
body mass class
BMI
anti-infectives during COVID-19
age at admission
hospital stay
WHO ordinal severity scale
sex
ICU stay
COVID-19 severity
any CT findings
reticulation
CTSS
GGO
opacity, AI
high opacity, AI
bronchiectasis
Kendall's τ
0.4
0.6
0.8
1.0
variable classifcation
risk factors and CoV severity
symptoms
time after CoV
CT readouts and findings
B
CTSS
r = 0.92, p < 0.001
points
no
n = 187
yes
n = 233
GGO
CTSS
r = 0.87, p < 0.001
points
no
n = 213
yes
n = 207
reticulation
Opacity, AI
r = 0.75, p < 0.001
% of lung
no
n = 187
yes
n = 233
GGO
Opacity, AI
r = 0.78, p < 0.001
% of lung
no
n = 213
yes
n = 207
reticulation
