# Supplementary material for: Machine Learning Based Multi-Parameter Modeling for Prediction of Post-Inflammatory Lung Changes
Source: Diagnostics (Basel). 2025 Mar 20;15(6):783. doi: 10.3390/diagnostics15060783 (PMC11941013; doi:10.3390/diagnostics15060783)
Supplement: Supplementary file 1 [file diagnostics-15-00783-s001.zip › figure_s3_cooccurrence_ct_findings.pdf]

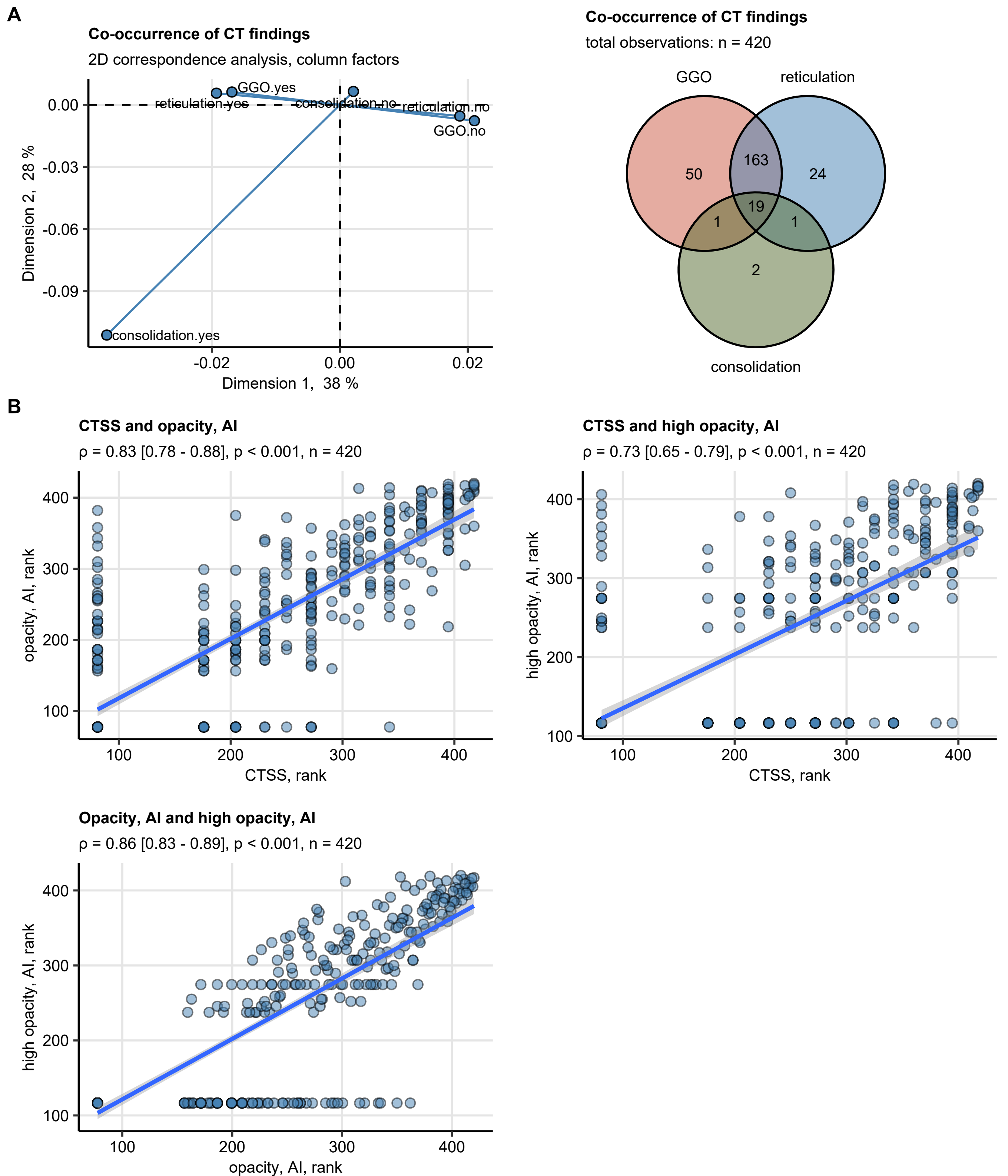

A
Co-occurrence of CT findings
2D correspondence analysis, column factors
GGO.yes
reticulation.yes
consolidation.no
reticulation.no
GGO.no
consolidation.yes
Dimension 1, 38 %
Dimension 2, 28 %
Co-occurrence of CT findings
total observations: n = 420
GGO
reticulation
consolidation
50
163
24
19
1
1
2
B
CTSS and opacity, AI
ρ = 0.83 [0.78 - 0.88], p < 0.001, n = 420
CTSS, rank
opacity, AI, rank
CTSS and high opacity, AI
ρ = 0.73 [0.65 - 0.79], p < 0.001, n = 420
CTSS, rank
high opacity, AI, rank
Opacity, AI and high opacity, AI
ρ = 0.86 [0.83 - 0.89], p < 0.001, n = 420
opacity, AI, rank
high opacity, AI, rank
