# Supplementary material for: Machine Learning Based Multi-Parameter Modeling for Prediction of Post-Inflammatory Lung Changes
Source: Diagnostics (Basel). 2025 Mar 20;15(6):783. doi: 10.3390/diagnostics15060783 (PMC11941013; doi:10.3390/diagnostics15060783)
Supplement: Supplementary file 1 [file diagnostics-15-00783-s001.zip › figure_s6_cooccurrence_lft_findings.pdf]

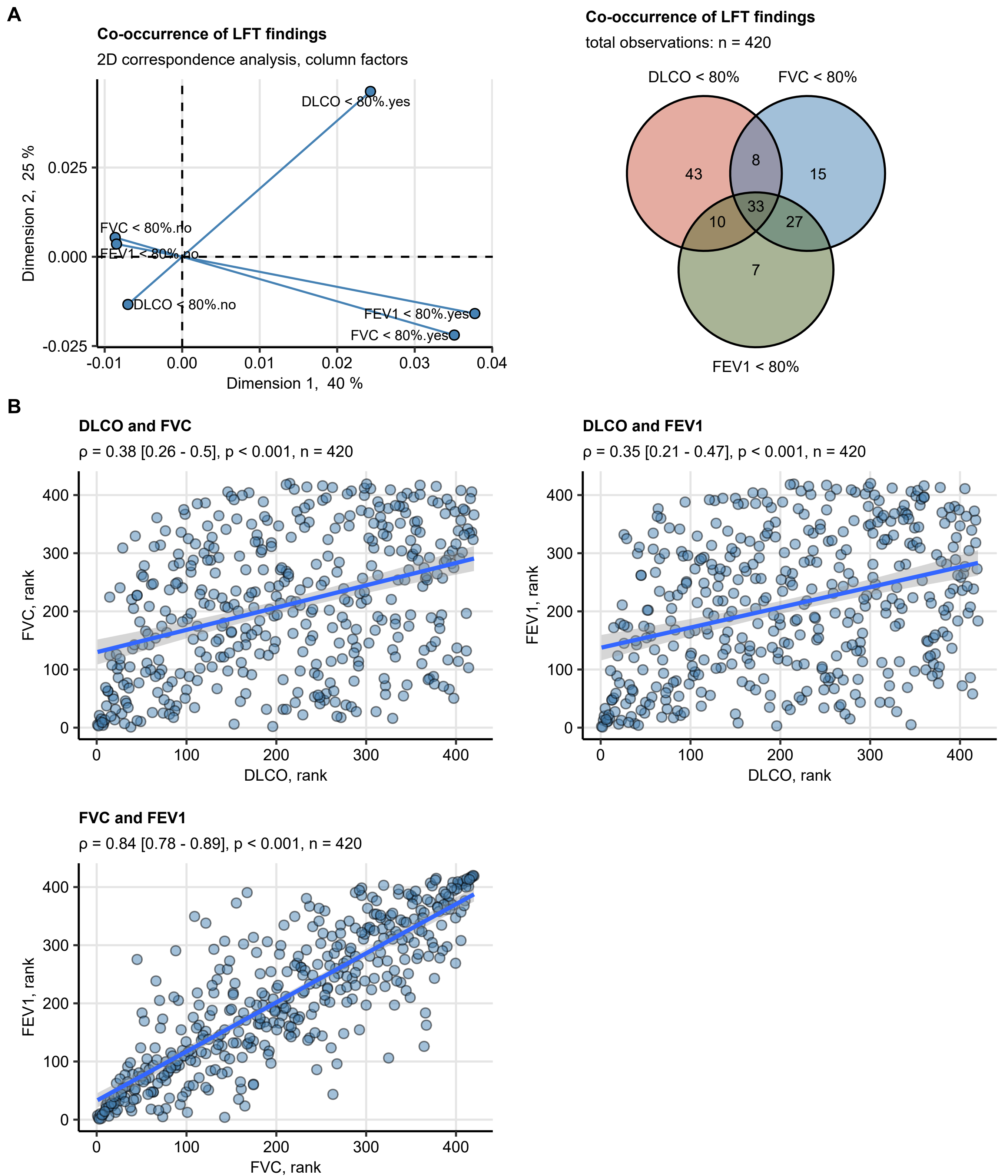
A
Co-occurrence of LFT findings
2D correspondence analysis, column factors
DLCO < 80%.yes
FVC < 80%.no
FEV1 < 80%.no
DLCO < 80%.no
FEV1 < 80%.yes
FVC < 80%.yes
Dimension 2, 25 %
Dimension 1, 40 %
0.025
0.000
-0.025
-0.01
0.00
0.01
0.02
0.03
0.04
Co-occurrence of LFT findings
total observations: n = 420
DLCO < 80%
FVC < 80%
FEV1 < 80%
43
8
15
33
10
27
7
B
DLCO and FVC
ρ = 0.38 [0.26 - 0.5], p < 0.001, n = 420
FVC, rank
DLCO, rank
DLCO and FEV1
ρ = 0.35 [0.21 - 0.47], p < 0.001, n = 420
FEV1, rank
DLCO, rank
FVC and FEV1
ρ = 0.84 [0.78 - 0.89], p < 0.001, n = 420
FEV1, rank
FVC, rank
0
100
200
300
400
