# Supplementary material for: Machine Learning Based Multi-Parameter Modeling for Prediction of Post-Inflammatory Lung Changes
Source: Diagnostics (Basel). 2025 Mar 20;15(6):783. doi: 10.3390/diagnostics15060783 (PMC11941013; doi:10.3390/diagnostics15060783)
Supplement: Supplementary file 1 [file diagnostics-15-00783-s001.zip › figure_s9_tuning_classification_reduced_dlco.pdf]

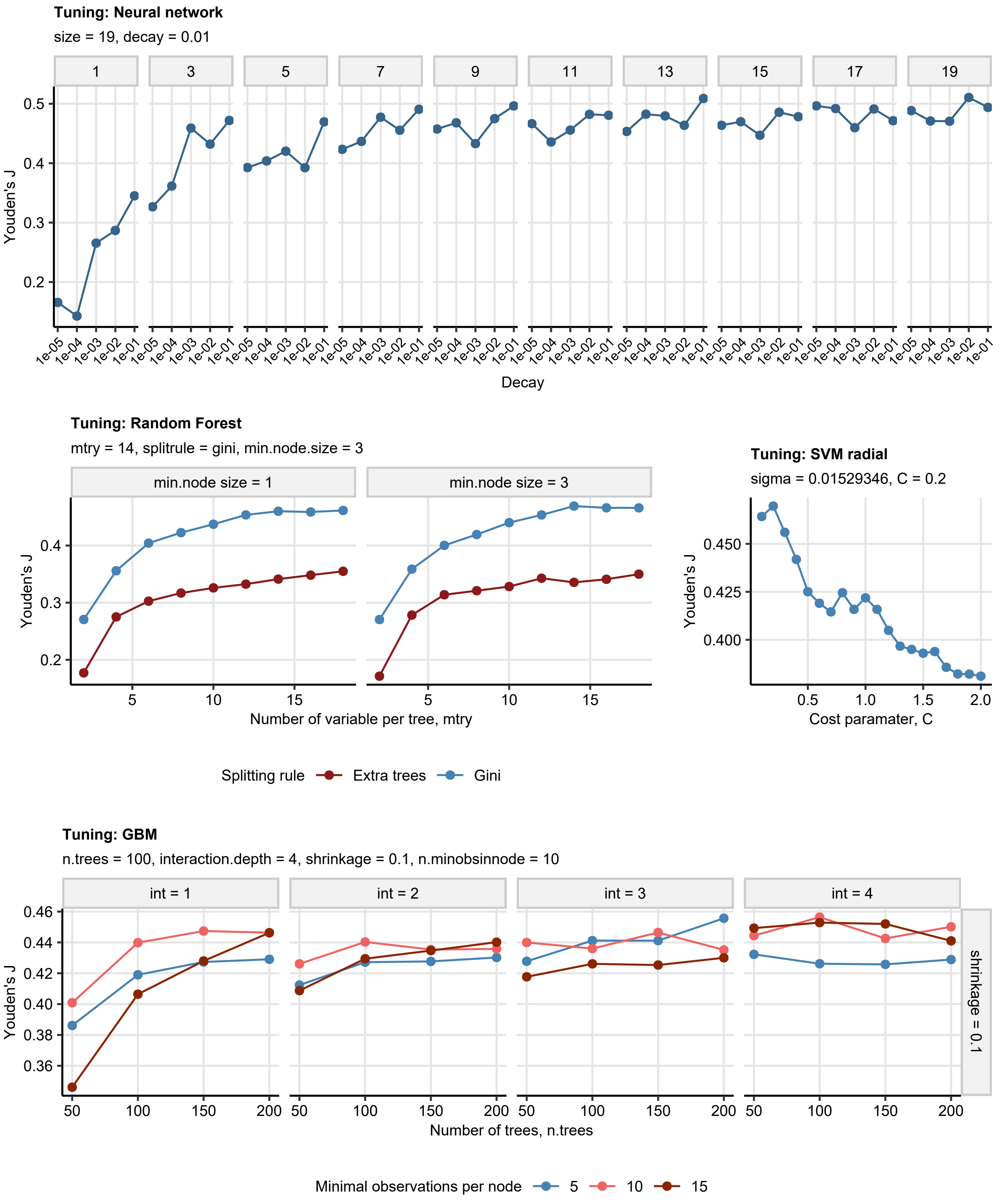
Tuning: Neural network
size = 19, decay = 0.01
1
3
5
7
9
11
13
15
17
19
Youden's J
0.2
0.3
0.4
0.5
1e-05
1e-04
1e-03
1e-02
1e-01
Decay
Tuning: Random Forest
mtry = 14, splitrule = gini, min.node.size = 3
min.node size = 1
min.node size = 3
Youden's J
0.2
0.3
0.4
5
10
15
Number of variable per tree, mtry
Tuning: SVM radial
sigma = 0.01529346, C = 0.2
Youden's J
0.400
0.425
0.450
0.5
1.0
1.5
2.0
Cost paramater, C
Splitting rule
Extra trees
Gini
Tuning: GBM
n.trees = 100, interaction.depth = 4, shrinkage = 0.1, n.minobsinnode = 10
int = 1
int = 2
int = 3
int = 4
shrinkage = 0.1
Youden's J
0.36
0.38
0.40
0.42
0.44
0.46
50
100
150
200
Number of trees, n.trees
Minimal observations per node
5
10
15
