# Supplementary material for: Machine Learning Based Multi-Parameter Modeling for Prediction of Post-Inflammatory Lung Changes
Source: Diagnostics (Basel). 2025 Mar 20;15(6):783. doi: 10.3390/diagnostics15060783 (PMC11941013; doi:10.3390/diagnostics15060783)
Supplement: Supplementary file 1 [file diagnostics-15-00783-s001.zip › supplementary_material.pdf]

Artificial intelligence-assisted analysis of CT abnormalities during COVID-19 recovery

Supplementary Material

Department of Radiology, Medical University of Innsbruck

2025-03-15

Supplementary Methods

Ethics

The CovILD study was conducted in accordance with the Declaration of Helsinki and the European Data policy. All participants gave written informed consent to participate and to process their data. The study data were processed, stored and analyzed in anonymized form. The study protocol was approved by the ethics committee of the Medical University of Innsbruck, Austria (approval number: 1103/2020). The study was registered at ClinicalTrials.gov (NCT04416100).

Study design and participants, handling of missing data

The longitudinal observation CovILD study aimed at investigation of symptom, cardiopulmonary and mental health recovery of COVID-19 patients. The details of the study design and the cohort are provided in our recent publications (1–4). In brief, n = 145 convalescent COVID-19 survivors were recruited among patients of the University Hospital of Innsbruck, St. Vinzenz Hospital in Zams and Karl-Landsteiner Rehabilitation Center in Münster (all in Austria) between March and June 2020. The inclusion criteria were age ≥ 18 years, SARS-CoV-2 positivity confirmed by PCR and presence of COVID-19 symptoms. All participants were infected with the wild-type form of SARS-CoV-2. The study visits were scheduled at two, three, six, and twelve months after COVID-19 diagnosis. In the current analysis, participant-matched longitudinal observations were utilized. The analysis inclusion criterion was availability of data from computed tomography (CT) of the chest and lung function testing (LFT). This criterion was fulfilled by 420 observations obtained from 140 CovILD study participants. For the patients included in the analysis, the set of explanatory variables concerning demographic and clinical background as well as the course and treatment of acute COVID-19 was complete.

The study design and analysis inclusion process are summarized in a flow chart in **Figure 1**. Numbers of observations at study follow-up examinations are presented in **Supplementary Table S1**. Analyzed variables are listed in **Supplementary Table S2**. Baseline demographic and clinical characteristic of the study cohort and characteristic of acute COVID-19 are provided in **Table 1**. Frequency of CT and LFT abnormalities, scoring of CT lesions and values of LFT readouts are listed in **Supplementary Tables S3** and **S4**. Frequencies of COVID-19 -related symptoms of relevance for lung function are presented in **Supplementary Table S5**.

Study procedures and data sources

Information on demography (age, sex), smoking status, routine medication, pre-existing conditions, as well as the course and treatment of acute COVID-19 were obtained in an interview and retrieved from electronic patient's record at the two-month follow-up visit. The standard follow-up visit protocol included a survey of symptoms and self-reported physical performance, examination and interview by a physician, determination of standard blood markers (hemoglobin, iron turnover, complete blood count, markers of inflammation and vascular pathology), trans-thoracic echocardiography, LFT, and CT of the chest. The details of the study procedures are provided in our recent publications (1–4). The complete list of study variables with their format and descriptions is provided in **Supplementary Table S2**.

Regarding the severity of acute COVID-19, the study participants were classified as 'ambulatory' (home isolated, WHO ordinal scale for clinical improvement: 1 - 2), 'hospitalized moderate' (hospitalized, without oxygen therapy or oxygen by mask or nasal prongs, WHO: 3 - 4), and 'hospitalized severe' (hospitalized, high flow oxygen or mechanical ventilation, WHO: 5 - 7).

Chest CT was done with a 128 slice multi-detector SOMATOM Definition Flash device (Siemens Healthineers, Erlangen, Germany) with a 128 × 0.6 mm collimation and spiral pitch factor of 1.1. Scans were obtained in craniocaudal direction without iodine contrast agent and in low-dose setting (100 kVp tube potential). Artificial intelligence (AI) supported analysis of the lung CT scans was done with the Syngo.via CT Pneumonia Analysis Software (Siemens Healthineers, Erlangen, Germany), which returned percentage of the lungs with opacity and high opacity. Evaluation of the CT scans by the radiologists was done in accordance with the Fleischner society guidelines as described before (1,3,5). In brief, the scans were examined for presence of ground glass opacity (GGO), reticulation, consolidations, and bronchiectasis. Severity of lung abnormalities was rated by the radiologist with the CT severity score (CTSS) described before (1,3). For computation of CTSS, radiological abnormalities were scored separately for each lobe with the following scheme:

- 0: no abnormalities
- 1: minimal, subtle GGO
- 2: mild, several GGO and subtle reticulations
- 3: moderate, multiple GGO, reticulation, small consolidation
- 4: severe, extensive GGO, consolidation, reticulation with distortion
- 5: massive findings, parenchymal destruction

CTSS was calculated as a sum of the scores over all lobes and ranged from 0 to 25 (1,3).

The following LFT parameters were recorded: diffusion capacity for carbon monoxide (DLCO), forced vital capacity (FVC), forced expiratory volume in one second (FEV1), total lung capacity (TLC), and FEV1 to FVC ratio (FEV1:FVC). Among them, DLCO, FVC, and FEV1, whose abnormalities were the most frequent in the CovILD cohort, were analyzed in the current report. These variables were expressed as percentage of the patient's individual age-, sex- and weight-specific reference. Insufficiency of DLCO, FVC, or FEV1 were defined as values below 80% of the reference (1,2,4).

The following longitudinally recorded symptoms of potential relevance for lung function were evaluated: dyspnea measured by Modified Medical Research Council scale (mMRC, presence of dyspnea defined as mMRC > 0), self-reported cough, and self-rated physical performance measured by Eastern Cooperative Oncology Group (ECOG, impaired physical performance defined as ECOG > 0) (4). Missing information on the symptoms or their intensity in the patient's symptom survey were interpreted as absence of the complaint.

The source study data set was fetched from the CovILD study data repository available as an R package to authorized users.

Software

The analysis was done with R version 4.2.3. Tabular data and code pipelines were handled by tools provided by the packages *tidyverse* (6), *rlang* (7), and *trafo*. Text data were handled with *stringi* (8). Parallelization was accomplished with *furrr* (9) and *doParallel* (10).

In exploratory data analysis, statistical hypothesis testing and analysis of correspondence and correlations, the packages *rstatix* (11), *rcompanion* (12), *MASS* (13), *clustTools*, and *ExDA* were used. Graph analyses were done with *igraph* (14) and development package *graphExtra*.

Receiver-operating characteristic (ROC), confusion matrix analysis and inter-rater reliability analyses were done with *caret* (15), *OptimalCutpoints* (16) and *bootStat*.

For machine learning modeling of lung function testing outcomes, the packages *caret* (15) and *caretExtra* were utilized, which provide wrappers around the R implementations of the random forest algorithm (*ranger*) (17,18), neural network (*nnet*) (19), support vector machines (SVM, *kernlab*) (20,21), and gradient boosted machines (GBM, *gbm*) (22–24). Model diagnostic statistics and performance evaluation metrics were computed with the development package *caretExtra*. SHAP (Shapley additive explanations) as a metric of global variance importance in machine learning models was calculated with the package *kernelshap* (25–27). Moving averages of statistics of performance of machine learning models were computed with *zoo* (28).

Analysis results were visualized with *ggplot* (6) and *ExDA* (scatter, stack and box plots), *clustTools* (visualization of results of correspondence analysis), *ggvenn* (29) (Venn plots), *caretExtra* (plots for machine learning model diagnostic and evaluation), *plotROC* (30) (ROC curves), *shapviz* (27,31) (violin/scatter plots of absolute SHAP values), and *graphExtra* (graph visualizations, re-implementation of tools from *igraph* and *ggnetwork*) (14,32). Result tables were created with *flextable* (33). Figures were generated with *cowplot* (34). Parts of the manuscript and supplementary material were written in the *rmarkdown* environment (35) with the *bookdown* package (36). Figures, tables, links and R expressions in the Rmarkdown document were managed with *figur*.

Descriptive statistic, statistical tests and effect sizes, correspondence analysis

If not stated otherwise, numeric values are presented as medians with interquartile ranges and ranges within the analysis data set or analysis strata. Qualitative variables are presented as percentages and counts of the category within the analysis data set or analysis strata. Normality of distribution of numeric variables upon identity, logarithm and square root transformation was checked with Shapiro-Wilk test (function `shapiro_test`, package *rstatix*). Since multiple numeric variables were not normally distributed, Mann-Whitney test or Kruskal-Wallis test were routinely used for comparison of independently distributed numeric variables between analysis groups. Differences in distribution of categorical variables between the study groups were assessed with χ^2 test. P values were adjusted for multiple testing with the false discovery rate method separately for each analysis task (e.g. comparison of explanatory variables between observations with and without LFT findings) (37). Effects with $p < 0.05$ were considered significant.

The following effect size metrics were used to assess differences between analysis groups and to evaluate performance of machine learning models (38–40):

- biserial r was used for two-group comparisons of numeric variables: < 0.3: small, 0.3 - 0.5: moderate, ≥ 0.5: large effect size
- η^2 was used for multi-group comparison of numeric variables: < 0.13: small, 0.13 - 0.26: moderate, ≥ 0.26: large effect size
- Cramer's V was used for comparisons of categorical variables: < 0.3: small, 0.3 - 0.5: moderate, ≥ 0.5: large effect size
- Spearman's ρ coefficient of correlation: < 0.3: small, 0.3 - 0.5: moderate, ≥ 0.5: large effect size
- Kendall's τ coefficient of correlation: < 0.1: small, 0.1 - 0.3: moderate, ≥ 0.3: large effect size

- pseudo-R^2 was used as a measure of explained variance of a model: < 0.13: small, 0.13 - 0.26: moderate, ≥ 0.26: large effect size

- Cohen's κ inter-rater reliability statistic: < 0.20: no effect, 0.2 - 0.4: fair, 0.4 - 0.6: moderate, 0.6 - 0.8: good, ≥ 0.8: excellent concordance

Statistical significance and effect sizes for comparisons and correlations of independent observations was assessed with the functions `compare_variables()` and `correlate_variables()` (package *ExDA*). Because of non-independent observations included in the analysis, pairwise correlations of numeric LFT or CT variables were investigated with blocked bootstrap Spearman's rank and Kendall' tests described below. Analogically, for comparison of medians between two subsets of non-independent observations, blocked bootstrap test was utilized. Please refer to **Comparison of CT readouts between observations with and without LFT abnormality, correlation analysis** for details.

Co-occurrence of LFT abnormalities (DLCO < 80% reference, FVC < 80% of reference, and FEV1 < 780% of reference) was investigated by two-dimensional correspondence analysis (function `mca()`, package *MASS*) (13) and whose column factors were visualized in a scatter plot with functions from the *clustTools* package. Additionally, counts of observations with co-occurring LFT abnormalities were visualized in Venn plot (function `ggvenn()`, package *ggvenn*).

Multi-parameter modeling of lung function

Presence of reduced DLCO, FVC and FEV1 (each < 80% of reference), as well as values of DLCO, FVC and FEV1 expressed as percentages of the reference value were modeled with 37 explanatory variables including:

- baseline demographic and clinical characteristic (e.g. age, sex, smoking, comorbidities)

- explanatory variables referring to the course of acute COVID-19 (e.g. WHO ordinal scale for clinical improvement, hospitalization status and length, treatment)

- follow-up after COVID-19 diagnosis (two, three, six and twelve months)

- presence and rating of longitudinally recorded symptoms of relevance for lung function (dyspnea, cough, impaired physical performance)

- presence and severity of lung CT findings rated by the radiologist (e.g. GGO, consolidations, CTSS) and AI (opacity and high opacity)

To reduce the number of explanatory variables, some qualitative variables with rare categories were re-coded. This concerned:

- *pulmonary illness* which subsumed pre-existing chronic obstructive lung disease (COPD), bronchial asthma, interstitial lung disease, chronic lung diseases and other pulmonary conditions
- *anti-coagulants during COVID-19* which subsumed anti-coagulation and anti-platelet treatment during acute COVID-19
- *anti-infectives during COVID-19* which subsumed anti-infective and macrolide treatment during acute COVID-19
- *smoking history*, where ex- and active smokers were grouped together

Severity of acute COVID-19 measured by the WHO ordinal scale of clinical improvement was re-coded as categorical explanatory variable. The response and explanatory variables are listed in **Supplementary Table S2**, the modeling strategy is schematically summarized in **Supplementary Figure S8**.

Multi-parameter models were constructed with four machine learning algorithms employing diverse mathematical principles: canonical random forest (implemented in R by the *ranger* package, caret's method name: 'ranger') (17,18), GBM (gradient boosted machines, *gbm* package, caret's method name: 'gbm') (22–24), neural network with a single hidden layer (*nnet* package, caret's method name: 'nnet') (19), and SVM with radial kernel (support vector machines, *kernlab* package, caret's method name: 'svmRadial') (20,21). For random forest models, 1000 random trees per model were generated. Bernoulli and Gaussian loss functions were implemented in classification and regression GBM models, respectively.

Hyper-parameters that control the modeling algorithm's behavior were found by searching grids of hyper-parameter values with maximizing the Youden's J statistic ($J = Sensitivity + Specificity - 1$, classification models of binary LFT outcomes) or by minimizing mean absolute error (MAE, regression models of numeric LFT outcomes) in 10-repeats 10-fold cross-validation, in a process called tuning. The tunable hyper-parameters were:

- for random forest, `mtry`: number of randomly chosen explanatory variables pre decision tree in the random forest ensemble, `splitrule`: criterion for splits of the explanatory variables at branches of the decision trees, `min.node.size`: minimal size of the node (leaf) of the decision tree
- for GBM, `n.trees`: number of decision trees in the GBM ensemble, `interaction.depth`: number of splits in the decision trees, `shrinkage`: regularization penalty applied to predictions made by single decision trees, `n.minobsinnode`: minimal size of the node (leaf) of the decision tree
- for neural network, `size`: number of neurons in the hidden layer, `decay`: reduction of weights during algorithm iteration

- for SVM, `sigma`: width of the radial kernel, `C`: cost/penalty applied to misclassified observations. For the SVM algorithm, the optimal value of `sigma` was selected by running `sigest()` function from *kernlab* package (21) on the modeling data set and kept constant in the tuning grid.

To account for the participant matching of the observations in the cross-validated tuning of hyper-parameters, a blocked cross-validation design was applied to keep all observations obtained from a particular participant either in the training or in the test portion of the cross-validation split. Construction of such 'participant-wise' blocked cross-validation folds was done with an in-house-developed wrapper around `createMultiFolds()` function (package *caret*). For tuning and training of the machine learning models, the `train()` wrapper from the *caret* package was employed; the tuning/training setting such as participant-blocked indexes for cross-validation folds and Youden's J cost function was specified by via a `trainControl()` object provided to the `train()` function (15). Of note, similar tuning strategy involving cross-validation or repeated cross-validation with *caret* was applied in other reports in the field of respiratory medicine (41–43). The complete tuning grids, i.e. all investigated combinations of the ghyper-parameters, with the corresponding values of Youden's J and MAE cost functions are listed in **Supplementary Table S6**. The optimal values of the tuning parameters are listed in **Supplementary Table S7**.

Performance of the machine learning models was evaluated by comparison of the model predictions with the observed outcome in the genuine training data set and in participant-wise blocked 10-repeats 10-fold cross-validation. Performance of the classification models was assessed by accuracy statistic defined as a fraction of correct predictions, Cohen's κ as a measure of concordance between the predicted and observed outcome (38,40), standard receiver operating characteristic metrics (AUC, overall accuracy, sensitivity, specificity) to assess the ability of the model to detect the pathological outcome (15), and with Brier score to investigate credibility of the predictions and model calibration (44,45) (**Table 2** and **Supplementary Table S8**). Performance of the regression models was assessed with mean absolute error (MAE) as a measure of fit quality, pseudo-R^2 as a metric of explained variance and Spearman's ρ for correlation between the predicted and observed outcome as as measure of overall model calibration (15) (**Table 3** and **Supplementary Table S9**). Pseudo-R^2 was computed with the following formulas:

$$pseudoR^2 = 1 - \frac{MSE(y)}{Var(y)}$$

$$MSE(y) = \frac{1}{N} \sum_{i}^{N} (y_i - \hat{y}_i)^2$$

where $MSE(y)$ stands for mean squared error of the outcome variable y, $Var(y)$ is the variance of the outcome variable y, N is the total observation number, i represents the observation index, y_i is the observed outcome value for the i-th observation, and $\hat{y}_i$ is the

predicted outcome value for the i-th observation. Performance statistics were retrieved from the `caret` models with the `summary()` method from the *caretExtra* package. Visualization of the model tuning and evaluation results was accomplished with the method `plot()` from the *caretExtra* package and in-house developed, project-specific functions.

In order to assess the model accuracy as a function of acute COVID-19 severity and time after diagnosis, we computed Cohen's κ (classification models) and MAE (regression models) of out-of-fold predictions for observations stratified by acute COVID-19 severity (mild, moderate, severe) and follow-up visit (2-, 3-, 6- and 12-month follow-up). The error values were subsequently visualized as heat maps.

Performance of the machine learning models at prediction of reduced DLCO < 80% of the patient's reference was assessed in observations with increasing values of DLCO expressed as percentage of the patient's reference. To this end, we sorted the observations by increasing DLCO values and computed moving averages of accuracy and squared distance to 0/1-coded DLCO < 80% outcome. The moving averages were computed with the sliding window of k = 20 observations (function `rollmean()` package *zoo*). The squared distance to the outcome were calculated with the following formula:

$$SqDist = (p - o)^2$$

where p stands for probability of DLCO < 80%, and o equals to 1 for DLCO < 80% and 0 for DLCO ≥ 80%.

For assessment of potential over- and under-fitting of predictions made by the machine learning models, we resorted to visualizations of learning curves (46). The learning curves were constructed as values of model performance metrics (classification models: accuracy and Cohen's κ,regression models: mean absolute error and pseudo-R^2) as functions of increasing size of the training data derived from the genuine COVILD cohort data set used for model construction. The performance metrics were computed for the training subset, a test subset (1/4 of observations not used for training), and out-of-fold predictions in 10-repeats 10-fold cross-validation. The learning curves were computed with `learning_curve_dat()` function from *caret* package and visualized as LOESS trends (Locally Estimated/weighted Scatterplot Smoothing).

Variable importance for multi-parameter models of lung function

Shapley additive explanations (SHAP) were used as a global importance statistic for the model's explanatory variables. In brief, SHAP measures contribution of the given variable to the model fit by comparing a goodness-of-fit statistic between models re-fitted with subsets of explanatory variables including the variable of interest and models re-fitted with subsets of explanatory variables without the variable of interest (25,26). Matrices of SHAP values for observations and variables were computed for the machine learning models of

lung function outcomes with the function `kernelshap()` provided by the *kernelshap* package (31). As a background for SHAP calculation, a single observation data frame was used with numeric explanatory variables set to the 25th percentile of the values in the entire data set and with qualitative explanatory variables set to the baseline category (31). For mean absolute SHAP values of the most influential explanatory variables in the Random Forest, neural network, SVM, and GBM models, see: **Table 10**. Visualization of the mean SHAP values for the top most influential explanatory variables was accomplished with the `shapviz()`, `sv_importance()` (package *shapviz*) (27), and in-house developed functions.

Co-linearity of explanatory variables

The most influential explanatory factors for predictions of DLCO (algorithms: random forest, GBM, neuronal network, and SVM radial) and DLCO < 80% of reference (algorithms: random forest, GBM, SVM radial) were identified as top 15 variables with the largest mean absolute SHAP importance values for each of the modeling algorithms. Associations between these highly influential variables were assessed with a soft-threshold weighted graph of correlations, a method widely used in omics and system biology (14,47). For construction of the correlation graph, ordered categorical variables (i.e. factors in R) were converted to integers. The graph edges were defined by pairwise correlations between the variables with tie-adjusted Kendall's $\tau \geq 0.3$. The edge weights corresponded to values of the τ correlation coefficients. The graph was constructed with `as_iGraph()` function, the correlation matrix was computed with `cor()` function from *stats* package passed to`as_iGraph()`. Isolated vertices, i.e. variables without any correlations with $\tau \geq 0.3$ were removed from the graph with `prune_degree()` (both functions from *graphExtra*). The graph was visualized with Fruchterman-Reingold algorithm (method `plot()`, package *graphExtra*).

Comparison and correlation of CT readouts

Because observations were non-independent, i.e. participant-matched, comparison of CTSS, opacity and high opacity of the lung between data points with and without LFT and CT abnormalities could not be investigated by standard statistical tests (e.g. Mann-Whitney test) and required instead a special approach. Differences in median of the CT parameters between observations with and without LFT or CT abnormalities were investigated with blocked bootstrap test with biserial r effect size statistic (H0: no difference in CT parameter, H1: CT parameter values are higher in observations with the LFT abnormality). The blocked bootstrap scheme involved re-sampling with repetition of the participants instead of single observations (B = 2000 re-samples). P values were obtained by counting re-samples in which the test hypothesis H1 was not met and dividing the count by the total re-sample number. The effect size statistic was computed as an arithmetic average of effect sizes in the re-samples.

A similar blocked bootstrap approach was applied in a correlation analysis of the CT (CTSS, opacity, high opacity) and LFT readouts (DLCO, FVC, FEV1, expressed as percentages of reference values). The H0 null hypothesis of such correlation test was no or positive correlation of a CT and LFT readout. The H1 hypothesis was the presence of negative correlation between the CT and LFT readout. Spearman's ρ correlation coefficients were computed for the bootstrap re-samples (B = 2000). The p values were obtained by counting re-samples with $\rho \geq 0$ and dividing the count by the total re-sample number. The ρ values were averaged and their confidence values were calculated as the 2.5 - 97.5 percentile range.

Bootstrap tests were performed with a functional programming interface implemented in the development package *bootStat*. The analysis results are listed in **Supplementary Tables S11** and **S14**.

Inter-rater reliability and receiver-operating characteristic analysis

The optimal cutoffs of human-determined CTSS, and AI-determined lung opacity and high opacity for detection of insufficient DLCO (< 80% of reference) were computed with the maximum of the Youden's J statistic ($J = Sensitivity + Specificity - 1$) in the entire analysis data set (function `optimal.cutpoints()`, package *OptimalCutpoints*) (16). Those optimal cutoffs were subsequently used to dichotomize the analyzed observations. Concordance between the CTSS^{high}, opacity^{high} and high opacity^{high} strata assignment and presence of LFT insufficiency of reference was assessed by Cohen's κ inter-rater reliability statistic (38,40) as well as sensitivity, specificity and AUC metrics. Those statistics were computed with the function `multiClassSummary()` from the *caret* package (15). Boundaries of the statistic's 95% confidence intervals were obtained by blocked bootstrap with 2000 iterations and were defined by the 2.5 and 97.5 percentiles of the blocked bootstrap estimates. Bootstrapping was accomplished by a functional interface implemented in the development package *bootStat*. The analysis results are listed in **Table 4**.

Data and code availability

The entire R analysis pipeline is available as a GitHub repository. The CovILD data set will be made available on reasonable request to the corresponding author.

Supplementary Tables

Supplementary Table S1: Numbers of observations included in the analysis split by acute COVID-19 severity and the follow-up examination.

Follow-up, months	Cohort	Ambulatory, mild COVID-19	Hospitalized, moderate COVID-19	Hospitalized, severe COVID-19
all time points	420	85	234	101
2-month	120	27	71	22
3-month	124	29	66	29
6-month	85	10	51	24
12-month	91	19	46	26

Supplementary Table S2: Study variables. The table is available as a supplementary Excel file.

Supplementary Table S3: Chest computed tomography variables at consecutive follow-ups. Numeric variables are presented as medians with interquartile ranges (IQR) and ranges. Categorical variables are presented as percentages and counts within the complete observation set.

Severity subset	Variable[a]	all time points	2-month follow-up	3-month follow-up	6-month follow-up	12-month follow-up
CovILD cohort	any CT findings	62% (n = 259)<br>complete: n = 420	71% (n = 85)<br>complete: n = 120	57% (n = 71)<br>complete: n = 124	64% (n = 54)<br>complete: n = 85	54% (n = 49)<br>complete: n = 91
	GGO	55% (n = 233)<br>complete: n = 420	69% (n = 83)<br>complete: n = 120	53% (n = 66)<br>complete: n = 124	52% (n = 44)<br>complete: n = 85	44% (n = 40)<br>complete: n = 91
	reticulation	49% (n = 207)<br>complete: n = 420	51% (n = 61)<br>complete: n = 120	48% (n = 60)<br>complete: n = 124	55% (n = 47)<br>complete: n = 85	43% (n = 39)<br>complete: n = 91
	consolidation	5.5% (n = 23)<br>complete: n = 420	11% (n = 13)<br>complete: n = 120	6.5% (n = 8)<br>complete: n = 124	1.2% (n = 1)<br>complete: n = 85	1.1% (n = 1)<br>complete: n = 91
	bronchiectasis	8.1% (n = 34)<br>complete: n = 420	10% (n = 12)<br>complete: n = 120	5.6% (n = 7)<br>complete: n = 124	8.2% (n = 7)<br>complete: n = 85	8.8% (n = 8)<br>complete: n = 91
	CTSS, points	2 [IQR: 0 - 8]<br>range: 0 - 20<br>complete: n = 420	5 [IQR: 0 - 13]<br>range: 0 - 20<br>complete: n = 120	2 [IQR: 0 - 7.2]<br>range: 0 - 18<br>complete: n = 124	2 [IQR: 0 - 5]<br>range: 0 - 15<br>complete: n = 85	1 [IQR: 0 - 5]<br>range: 0 - 15<br>complete: n = 91
	opacity, AI, % of lung	0.04 [IQR: 0 - 0.71]<br>range: 0 - 37<br>complete: n = 420	0.19 [IQR: 0 - 2.7]<br>range: 0 - 37<br>complete: n = 120	0.074 [IQR: 0 - 0.86]<br>range: 0 - 22<br>complete: n = 124	0.04 [IQR: 0 - 0.43]<br>range: 0 - 12<br>complete: n = 85	0 [IQR: 0 - 0.12]<br>range: 0 - 6.2<br>complete: n = 91
	high opacity, AI, % of lung	0 [IQR: 0 - 0.025]<br>range: 0 - 3.1<br>complete: n = 420	0.005 [IQR: 0 - 0.11]<br>range: 0 - 3.1<br>complete: n = 120	0.002 [IQR: 0 - 0.053]<br>range: 0 - 2.4<br>complete: n = 124	0 [IQR: 0 - 0.01]<br>range: 0 - 0.46<br>complete: n = 85	0 [IQR: 0 - 0.01]<br>range: 0 - 0.11<br>complete: n = 91
ambulatory	any CT findings	20% (n = 17)<br>complete: n = 85	26% (n = 7)<br>complete: n = 27	17% (n = 5)<br>complete: n = 29	30% (n = 3)<br>complete: n = 10	11% (n = 2)<br>complete: n = 19
	GGO	14% (n = 12)<br>complete: n = 85	22% (n = 6)<br>complete: n = 27	14% (n = 4)<br>complete: n = 29	10% (n = 1)<br>complete: n = 10	5.3% (n = 1)<br>complete: n = 19
	reticulation	11% (n = 9)<br>complete: n = 85	7.4% (n = 2)<br>complete: n = 27	6.9% (n = 2)<br>complete: n = 29	30% (n = 3)<br>complete: n = 10	11% (n = 2)<br>complete: n = 19
	consolidation	2.4% (n = 2)<br>complete: n = 85	3.7% (n = 1)<br>complete: n = 27	3.4% (n = 1)<br>complete: n = 29	0% (n = 0)<br>complete: n = 10	0% (n = 0)<br>complete: n = 19
	bronchiectasis	0% (n = 0)<br>complete: n = 85	0% (n = 0)<br>complete: n = 27	0% (n = 0)<br>complete: n = 29	0% (n = 0)<br>complete: n = 10	0% (n = 0)<br>complete: n = 19

Severity subset	Variable[a]	all time points	2-month follow-up	3-month follow-up	6-month follow-up	12-month follow-up
	CTSS, points	0 [IQR: 0 - 0]<br>range: 0 - 20<br>complete: n = 85	0 [IQR: 0 - 1]<br>range: 0 - 20<br>complete: n = 27	0 [IQR: 0 - 0]<br>range: 0 - 9<br>complete: n = 29	0 [IQR: 0 - 2.2]<br>range: 0 - 5<br>complete: n = 10	0 [IQR: 0 - 0]<br>range: 0 - 3<br>complete: n = 19
	opacity, AI, % of lung	0 [IQR: 0 - 0.02]<br>range: 0 - 14<br>complete: n = 85	0 [IQR: 0 - 0.04]<br>range: 0 - 14<br>complete: n = 27	0 [IQR: 0 - 0.04]<br>range: 0 - 1.2<br>complete: n = 29	0 [IQR: 0 - 0.025]<br>range: 0 - 0.17<br>complete: n = 10	0 [IQR: 0 - 0]<br>range: 0 - 0.16<br>complete: n = 19
	high opacity, AI, % of lung	0 [IQR: 0 - 0]<br>range: 0 - 1.9<br>complete: n = 85	0 [IQR: 0 - 0.001]<br>range: 0 - 1.9<br>complete: n = 27	0 [IQR: 0 - 0]<br>range: 0 - 0.12<br>complete: n = 29	0 [IQR: 0 - 0]<br>range: 0 - 0.03<br>complete: n = 10	0 [IQR: 0 - 0]<br>range: 0 - 0.05<br>complete: n = 19
moderate	any CT findings	65% (n = 151)<br>complete: n = 234	80% (n = 57)<br>complete: n = 71	61% (n = 40)<br>complete: n = 66	57% (n = 29)<br>complete: n = 51	54% (n = 25)<br>complete: n = 46
	GGO	59% (n = 137)<br>complete: n = 234	79% (n = 56)<br>complete: n = 71	56% (n = 37)<br>complete: n = 66	49% (n = 25)<br>complete: n = 51	41% (n = 19)<br>complete: n = 46
	reticulation	50% (n = 117)<br>complete: n = 234	55% (n = 39)<br>complete: n = 71	52% (n = 34)<br>complete: n = 66	47% (n = 24)<br>complete: n = 51	43% (n = 20)<br>complete: n = 46
	consolidation	5.6% (n = 13)<br>complete: n = 234	9.9% (n = 7)<br>complete: n = 71	9.1% (n = 6)<br>complete: n = 66	0% (n = 0)<br>complete: n = 51	0% (n = 0)<br>complete: n = 46
	bronchiectasis	6.4% (n = 15)<br>complete: n = 234	7% (n = 5)<br>complete: n = 71	7.6% (n = 5)<br>complete: n = 66	5.9% (n = 3)<br>complete: n = 51	4.3% (n = 2)<br>complete: n = 46
	CTSS, points	2 [IQR: 0 - 7]<br>range: 0 - 20<br>complete: n = 234	6 [IQR: 1.5 - 12]<br>range: 0 - 20<br>complete: n = 71	2 [IQR: 0 - 5.8]<br>range: 0 - 13<br>complete: n = 66	1 [IQR: 0 - 4.5]<br>range: 0 - 13<br>complete: n = 51	1 [IQR: 0 - 2.8]<br>range: 0 - 13<br>complete: n = 46
	opacity, AI, % of lung	0.04 [IQR: 0 - 0.53]<br>range: 0 - 22<br>complete: n = 234	0.21 [IQR: 0.0055 - 2]<br>range: 0 - 22<br>complete: n = 71	0.057 [IQR: 0 - 0.59]<br>range: 0 - 11<br>complete: n = 66	0.02 [IQR: 0 - 0.29]<br>range: 0 - 12<br>complete: n = 51	0 [IQR: 0 - 0.048]<br>range: 0 - 2<br>complete: n = 46
	high opacity, AI, % of lung	0 [IQR: 0 - 0.014]<br>range: 0 - 3.1<br>complete: n = 234	0.005 [IQR: 0 - 0.053]<br>range: 0 - 3.1<br>complete: n = 71	5e-04 [IQR: 0 - 0.027]<br>range: 0 - 2.4<br>complete: n = 66	0 [IQR: 0 - 0]<br>range: 0 - 0.46<br>complete: n = 51	0 [IQR: 0 - 0]<br>range: 0 - 0.11<br>complete: n = 46
severe	any CT findings	90% (n = 91)<br>complete: n = 101	95% (n = 21)<br>complete: n = 22	90% (n = 26)<br>complete: n = 29	92% (n = 22)<br>complete: n = 24	85% (n = 22)<br>complete: n = 26
	GGO	83% (n = 84)<br>complete: n = 101	95% (n = 21)<br>complete: n = 22	86% (n = 25)<br>complete: n = 29	75% (n = 18)<br>complete: n = 24	77% (n = 20)<br>complete: n = 26
	reticulation	80% (n = 81)	91% (n = 20)	83% (n = 24)	83% (n = 20)	65% (n = 17)

Severity subset	Variable[a]	all time points	2-month follow-up	3-month follow-up	6-month follow-up	12-month follow-up
		complete: n = 101	complete: n = 22	complete: n = 29	complete: n = 24	complete: n = 26
	consolidation	7.9% (n = 8)<br>complete: n = 101	23% (n = 5)<br>complete: n = 22	3.4% (n = 1)<br>complete: n = 29	4.2% (n = 1)<br>complete: n = 24	3.8% (n = 1)<br>complete: n = 26
	bronchiectasis	19% (n = 19)<br>complete: n = 101	32% (n = 7)<br>complete: n = 22	6.9% (n = 2)<br>complete: n = 29	17% (n = 4)<br>complete: n = 24	23% (n = 6)<br>complete: n = 26
	CTSS, points	10 [IQR: 3 - 14]<br>range: 0 - 20<br>complete: n = 101	14 [IQR: 10 - 15]<br>range: 0 - 20<br>complete: n = 22	10 [IQR: 5 - 15]<br>range: 0 - 18<br>complete: n = 29	6 [IQR: 2.8 - 10]<br>range: 0 - 15<br>complete: n = 24	5 [IQR: 2 - 11]<br>range: 0 - 15<br>complete: n = 26
	opacity, AI, % of lung	0.68 [IQR: 0.054 - 3.7]<br>range: 0 - 37<br>complete: n = 101	5.8 [IQR: 0.34 - 9.3]<br>range: 0 - 37<br>complete: n = 22	1.9 [IQR: 0.39 - 6.8]<br>range: 0 - 22<br>complete: n = 29	0.32 [IQR: 0.042 - 2]<br>range: 0 - 6.7<br>complete: n = 24	0.28 [IQR: 0 - 0.67]<br>range: 0 - 6.2<br>complete: n = 26
	high opacity, AI, % of lung	0.02 [IQR: 0 - 0.16]<br>range: 0 - 2.1<br>complete: n = 101	0.19 [IQR: 0.066 - 0.81]<br>range: 0 - 2.1<br>complete: n = 22	0.06 [IQR: 0.011 - 0.26]<br>range: 0 - 1<br>complete: n = 29	0 [IQR: 0 - 0.02]<br>range: 0 - 0.23<br>complete: n = 24	0.01 [IQR: 0 - 0.02]<br>range: 0 - 0.11<br>complete: n = 26

[a]any CT findings: any CT findings diagnosed by a human pathologist; GGO: ground glass opacity; CTSS: human-determined CT severity score; reticulation, consolidation, bronchiectasis: diagnosed by a human radiologist; opacity and high opacity, AI: opacity and high opacity determined by artificial intelligence.

Supplementary Table S4: Lung function testing variables at consecutive follow-ups. Numeric variables are presented as medians with interquartile ranges (IQR) and ranges. Categorical variables are presented as percentages and counts within the complete observation set.

Severity subset	Variable[a]	all time points	2-month follow-up	3-month follow-up	6-month follow-up	12-month follow-up
CovILD cohort	FVC, % of reference	90 [IQR: 82 - 98]<br>range: 46 - 130<br>complete: n = 420	88 [IQR: 80 - 97]<br>range: 46 - 130<br>complete: n = 120	90 [IQR: 83 - 97]<br>range: 54 - 120<br>complete: n = 124	91 [IQR: 84 - 98]<br>range: 50 - 120<br>complete: n = 85	90 [IQR: 84 - 99]<br>range: 55 - 120<br>complete: n = 91
	FVC < 80%	20% (n = 83)<br>complete: n = 420	24% (n = 29)<br>complete: n = 120	21% (n = 26)<br>complete: n = 124	18% (n = 15)<br>complete: n = 85	14% (n = 13)<br>complete: n = 91
	FEV1, % of reference	93 [IQR: 83 - 100]<br>range: 30 - 140<br>complete: n = 420	92 [IQR: 82 - 100]<br>range: 50 - 140<br>complete: n = 120	92 [IQR: 82 - 100]<br>range: 30 - 130<br>complete: n = 124	94 [IQR: 84 - 100]<br>range: 30 - 130<br>complete: n = 85	95 [IQR: 84 - 110]<br>range: 60 - 140<br>complete: n = 91
	FEV1 < 80%	18% (n = 77)<br>complete: n = 420	20% (n = 24)<br>complete: n = 120	21% (n = 26)<br>complete: n = 124	15% (n = 13)<br>complete: n = 85	15% (n = 14)<br>complete: n = 91
	DLCO, % of reference	94 [IQR: 82 - 110]<br>range: 31 - 140<br>complete: n = 420	91 [IQR: 77 - 110]<br>range: 44 - 140<br>complete: n = 120	94 [IQR: 83 - 110]<br>range: 31 - 140<br>complete: n = 124	95 [IQR: 83 - 110]<br>range: 32 - 140<br>complete: n = 85	93 [IQR: 86 - 110]<br>range: 48 - 130<br>complete: n = 91
	DLCO < 80%	22% (n = 94)<br>complete: n = 420	29% (n = 35)<br>complete: n = 120	22% (n = 27)<br>complete: n = 124	21% (n = 18)<br>complete: n = 85	15% (n = 14)<br>complete: n = 91
ambulatory	FVC, % of reference	94 [IQR: 86 - 100]<br>range: 46 - 110<br>complete: n = 85	95 [IQR: 87 - 100]<br>range: 46 - 110<br>complete: n = 27	94 [IQR: 87 - 100]<br>range: 64 - 110<br>complete: n = 29	91 [IQR: 86 - 100]<br>range: 64 - 110<br>complete: n = 10	95 [IQR: 85 - 100]<br>range: 72 - 110<br>complete: n = 19
	FVC < 80%	15% (n = 13)<br>complete: n = 85	11% (n = 3)<br>complete: n = 27	17% (n = 5)<br>complete: n = 29	10% (n = 1)<br>complete: n = 10	21% (n = 4)<br>complete: n = 19
	FEV1, % of reference	96 [IQR: 86 - 100]<br>range: 50 - 120<br>complete: n = 85	94 [IQR: 86 - 100]<br>range: 50 - 110<br>complete: n = 27	98 [IQR: 87 - 100]<br>range: 65 - 120<br>complete: n = 29	95 [IQR: 85 - 100]<br>range: 70 - 110<br>complete: n = 10	96 [IQR: 88 - 100]<br>range: 72 - 110<br>complete: n = 19
	FEV1 < 80%	13% (n = 11)<br>complete: n = 85	11% (n = 3)<br>complete: n = 27	14% (n = 4)<br>complete: n = 29	10% (n = 1)<br>complete: n = 10	16% (n = 3)<br>complete: n = 19
	DLCO, % of reference	100 [IQR: 92 - 110]<br>range: 52 - 140<br>complete: n = 85	110 [IQR: 91 - 120]<br>range: 52 - 140<br>complete: n = 27	100 [IQR: 96 - 110]<br>range: 69 - 130<br>complete: n = 29	99 [IQR: 89 - 110]<br>range: 77 - 130<br>complete: n = 10	98 [IQR: 92 - 110]<br>range: 74 - 120<br>complete: n = 19
	DLCO < 80%	12% (n = 10)<br>complete: n = 85	15% (n = 4)<br>complete: n = 27	10% (n = 3)<br>complete: n = 29	20% (n = 2)<br>complete: n = 10	5.3% (n = 1)<br>complete: n = 19
moderate	FVC, % of reference	90 [IQR: 82 - 98]<br>range: 46 - 130	87 [IQR: 80 - 96]<br>range: 46 - 130	90 [IQR: 84 - 98]<br>range: 54 - 110	96 [IQR: 84 - 99]<br>range: 50 - 120	91 [IQR: 85 - 99]<br>range: 55 - 120

Severity subset	Variable[a]	all time points	2-month follow-up	3-month follow-up	6-month follow-up	12-month follow-up
		complete: n = 234	complete: n = 71	complete: n = 66	complete: n = 51	complete: n = 46
	FVC < 80%	18% (n = 41) complete: n = 234	24% (n = 17) complete: n = 71	17% (n = 11) complete: n = 66	16% (n = 8) complete: n = 51	11% (n = 5) complete: n = 46
	FEV1, % of reference	94 [IQR: 84 - 100] range: 30 - 140 complete: n = 234	92 [IQR: 83 - 100] range: 60 - 140 complete: n = 71	92 [IQR: 85 - 100] range: 30 - 120 complete: n = 66	97 [IQR: 85 - 110] range: 30 - 130 complete: n = 51	95 [IQR: 87 - 110] range: 60 - 140 complete: n = 46
	FEV1 < 80%	15% (n = 36) complete: n = 234	17% (n = 12) complete: n = 71	17% (n = 11) complete: n = 66	12% (n = 6) complete: n = 51	15% (n = 7) complete: n = 46
	DLCO, % of reference	96 [IQR: 84 - 110] range: 31 - 140 complete: n = 234	93 [IQR: 79 - 100] range: 52 - 120 complete: n = 71	96 [IQR: 85 - 110] range: 31 - 140 complete: n = 66	100 [IQR: 89 - 110] range: 32 - 140 complete: n = 51	97 [IQR: 87 - 110] range: 54 - 130 complete: n = 46
	DLCO < 80%	20% (n = 47) complete: n = 234	25% (n = 18) complete: n = 71	20% (n = 13) complete: n = 66	18% (n = 9) complete: n = 51	15% (n = 7) complete: n = 46
severe	FVC, % of reference	86 [IQR: 77 - 93] range: 54 - 120 complete: n = 101	83 [IQR: 71 - 91] range: 58 - 110 complete: n = 22	86 [IQR: 71 - 91] range: 54 - 120 complete: n = 29	87 [IQR: 80 - 91] range: 61 - 110 complete: n = 24	86 [IQR: 83 - 93] range: 58 - 120 complete: n = 26
	FVC < 80%	29% (n = 29) complete: n = 101	41% (n = 9) complete: n = 22	34% (n = 10) complete: n = 29	25% (n = 6) complete: n = 24	15% (n = 4) complete: n = 26
	FEV1, % of reference	88 [IQR: 79 - 98] range: 60 - 130 complete: n = 101	83 [IQR: 75 - 97] range: 62 - 120 complete: n = 22	88 [IQR: 78 - 98] range: 60 - 130 complete: n = 29	88 [IQR: 83 - 95] range: 66 - 110 complete: n = 24	94 [IQR: 83 - 99] range: 63 - 120 complete: n = 26
	FEV1 < 80%	30% (n = 30) complete: n = 101	41% (n = 9) complete: n = 22	38% (n = 11) complete: n = 29	25% (n = 6) complete: n = 24	15% (n = 4) complete: n = 26
	DLCO, % of reference	85 [IQR: 71 - 92] range: 44 - 130 complete: n = 101	76 [IQR: 58 - 87] range: 44 - 130 complete: n = 22	84 [IQR: 72 - 90] range: 49 - 110 complete: n = 29	85 [IQR: 71 - 93] range: 50 - 110 complete: n = 24	89 [IQR: 83 - 97] range: 48 - 120 complete: n = 26
	DLCO < 80%	37% (n = 37) complete: n = 101	59% (n = 13) complete: n = 22	38% (n = 11) complete: n = 29	29% (n = 7) complete: n = 24	23% (n = 6) complete: n = 26

[a]LFT: lung function testing; FVC: forced vital capacity; FEV1: forced expiratory volume in one second; DLCO: diffusion capacity for carbon monoxide.

Supplementary Table S5: Presence and rating of symptoms of relevance for lung function at consecutive follow-ups. Numeric variables are presented as medians with interquartile ranges (IQR) and ranges. Categorical variables are presented as percentages and counts within the complete observation set.

Severity subset	Variable[a]	all time points	2-month follow-up	3-month follow-up	6-month follow-up	12-month follow-up
CovILD cohort	dyspnea rating, mMRC, points	0 [IQR: 0 - 1]<br>range: 0 - 4<br>complete: n = 420	0 [IQR: 0 - 1]<br>range: 0 - 4<br>complete: n = 240	0 [IQR: 0 - 1]<br>range: 0 - 3<br>complete: n = 248	0 [IQR: 0 - 1]<br>range: 0 - 4<br>complete: n = 170	0 [IQR: 0 - 0]<br>range: 0 - 4<br>complete: n = 182
	dyspnea, mMRC > 0	33% (n = 139)<br>complete: n = 420	45% (n = 108)<br>complete: n = 240	34% (n = 84)<br>complete: n = 248	27% (n = 46)<br>complete: n = 170	22% (n = 40)<br>complete: n = 182
	cough	16% (n = 68)<br>complete: n = 420	18% (n = 44)<br>complete: n = 240	17% (n = 42)<br>complete: n = 248	13% (n = 22)<br>complete: n = 170	15% (n = 28)<br>complete: n = 182
	physical performance, ECOG, points	0 [IQR: 0 - 1]<br>range: 0 - 4<br>complete: n = 420	1 [IQR: 0 - 1]<br>range: 0 - 4<br>complete: n = 240	0.5 [IQR: 0 - 1]<br>range: 0 - 4<br>complete: n = 248	0 [IQR: 0 - 1]<br>range: 0 - 1<br>complete: n = 170	0 [IQR: 0 - 1]<br>range: 0 - 2<br>complete: n = 182
	impaired physical performance, ECOG > 0	44% (n = 185)<br>complete: n = 420	53% (n = 128)<br>complete: n = 240	50% (n = 124)<br>complete: n = 248	33% (n = 56)<br>complete: n = 170	34% (n = 62)<br>complete: n = 182
ambulatory	dyspnea rating, mMRC, points	0 [IQR: 0 - 1]<br>range: 0 - 4<br>complete: n = 85	0 [IQR: 0 - 1.8]<br>range: 0 - 4<br>complete: n = 54	0 [IQR: 0 - 1]<br>range: 0 - 3<br>complete: n = 58	0 [IQR: 0 - 1]<br>range: 0 - 1<br>complete: n = 20	0 [IQR: 0 - 0]<br>range: 0 - 1<br>complete: n = 38
	dyspnea, mMRC > 0	36% (n = 31)<br>complete: n = 85	44% (n = 24)<br>complete: n = 54	38% (n = 22)<br>complete: n = 58	40% (n = 8)<br>complete: n = 20	21% (n = 8)<br>complete: n = 38
	cough	22% (n = 19)<br>complete: n = 85	22% (n = 12)<br>complete: n = 54	24% (n = 14)<br>complete: n = 58	20% (n = 4)<br>complete: n = 20	21% (n = 8)<br>complete: n = 38
	physical performance, ECOG, points	1 [IQR: 0 - 1]<br>range: 0 - 4<br>complete: n = 85	1 [IQR: 0 - 1]<br>range: 0 - 2<br>complete: n = 54	1 [IQR: 0 - 1]<br>range: 0 - 4<br>complete: n = 58	0 [IQR: 0 - 1]<br>range: 0 - 1<br>complete: n = 20	0 [IQR: 0 - 1]<br>range: 0 - 1<br>complete: n = 38
	impaired physical performance, ECOG > 0	52% (n = 44)<br>complete: n = 85	59% (n = 32)<br>complete: n = 54	59% (n = 34)<br>complete: n = 58	40% (n = 8)<br>complete: n = 20	37% (n = 14)<br>complete: n = 38
moderate	dyspnea rating, mMRC, points	0 [IQR: 0 - 1]<br>range: 0 - 4<br>complete: n = 234	0 [IQR: 0 - 1]<br>range: 0 - 4<br>complete: n = 142	0 [IQR: 0 - 1]<br>range: 0 - 3<br>complete: n = 132	0 [IQR: 0 - 0.75]<br>range: 0 - 3<br>complete: n = 102	0 [IQR: 0 - 0]<br>range: 0 - 4<br>complete: n = 92
	dyspnea,	31% (n = 73)	38% (n = 54)	36% (n = 48)	25% (n = 26)	20% (n = 18)

**Severity subset**	**Variable[a]**	**all time points**	**2-month follow-up**	**3-month follow-up**	**6-month follow-up**	**12-month follow-up**
	mMRC > 0	complete: n = 234	complete: n = 142	complete: n = 132	complete: n = 102	complete: n = 92
	cough	15% (n = 35)<br>complete: n = 234	17% (n = 24)<br>complete: n = 142	15% (n = 20)<br>complete: n = 132	12% (n = 12)<br>complete: n = 102	15% (n = 14)<br>complete: n = 92
	physical performance, ECOG, points	0 [IQR: 0 - 1]<br>range: 0 - 3<br>complete: n = 234	0 [IQR: 0 - 1]<br>range: 0 - 3<br>complete: n = 142	0 [IQR: 0 - 1]<br>range: 0 - 1<br>complete: n = 132	0 [IQR: 0 - 0.75]<br>range: 0 - 1<br>complete: n = 102	0 [IQR: 0 - 1]<br>range: 0 - 1<br>complete: n = 92
	impaired physical performance, ECOG > 0	36% (n = 84)<br>complete: n = 234	44% (n = 62)<br>complete: n = 142	41% (n = 54)<br>complete: n = 132	25% (n = 26)<br>complete: n = 102	28% (n = 26)<br>complete: n = 92
severe	dyspnea rating, mMRC, points	0 [IQR: 0 - 1]<br>range: 0 - 4<br>complete: n = 101	1 [IQR: 0 - 1]<br>range: 0 - 4<br>complete: n = 44	0 [IQR: 0 - 0]<br>range: 0 - 3<br>complete: n = 58	0 [IQR: 0 - 0.25]<br>range: 0 - 4<br>complete: n = 48	0 [IQR: 0 - 1]<br>range: 0 - 1<br>complete: n = 52
	dyspnea, mMRC > 0	35% (n = 35)<br>complete: n = 101	68% (n = 30)<br>complete: n = 44	24% (n = 14)<br>complete: n = 58	25% (n = 12)<br>complete: n = 48	27% (n = 14)<br>complete: n = 52
	cough	14% (n = 14)<br>complete: n = 101	18% (n = 8)<br>complete: n = 44	14% (n = 8)<br>complete: n = 58	12% (n = 6)<br>complete: n = 48	12% (n = 6)<br>complete: n = 52
	physical performance, ECOG, points	1 [IQR: 0 - 1]<br>range: 0 - 4<br>complete: n = 101	1 [IQR: 1 - 1]<br>range: 0 - 4<br>complete: n = 44	1 [IQR: 0 - 1]<br>range: 0 - 2<br>complete: n = 58	0 [IQR: 0 - 1]<br>range: 0 - 1<br>complete: n = 48	0 [IQR: 0 - 1]<br>range: 0 - 2<br>complete: n = 52
	impaired physical performance, ECOG > 0	56% (n = 57)<br>complete: n = 101	77% (n = 34)<br>complete: n = 44	62% (n = 36)<br>complete: n = 58	46% (n = 22)<br>complete: n = 48	42% (n = 22)<br>complete: n = 52

[a]mMRC: modified Medical Research Council scale; ECOG: Eastern Cooperative Oncology Group performance scale.

Supplementary Table S6: Selection of machine learning algorithm hyper-parameters by cross-validation-mediated tuning: the complete tuning grids. The selection criteria were the maximum of Youden's J statistic for the classification models. and the minimum of mean absolute error for the regression models. The table is available as a supplementary Excel file.

Supplementary Table S7: Selection of machine learning algorithm hyper-parameters by cross-validation-mediated tuning: the optimal combinations of the hyper-parameters.

**Response**[ab]	**Algorithm**	**Parameters**[c]
DLCO < 80%	Random Forest	mtry = 14<br>splitrule = gini<br>min.node.size = 3
	Neural network	size = 19<br>decay = 0.01
	Support vector machines, radial kernel	sigma = 0.0153<br>C = 0.2
	Gradient boosted machines	n.trees = 100<br>interaction.depth = 4<br>shrinkage = 0.1<br>n.minobsinnode = 10
FVC < 80%	Random Forest	mtry = 8<br>splitrule = gini<br>min.node.size = 3
	Neural network	size = 13<br>decay = 1e-04
	Support vector machines, radial kernel	sigma = 0.0153<br>C = 0.9
	Gradient boosted machines	n.trees = 150<br>interaction.depth = 3<br>shrinkage = 0.1<br>n.minobsinnode = 15
FEV1 < 80%	Random Forest	mtry = 14<br>splitrule = gini<br>min.node.size = 1
	Neural network	size = 11<br>decay = 1e-04
	Support vector machines, radial kernel	sigma = 0.0153<br>C = 0.1
	Gradient boosted machines	n.trees = 200<br>interaction.depth = 1<br>shrinkage = 0.1<br>n.minobsinnode = 5
DLCO	Random Forest	mtry = 6

Response[ab]	Algorithm	Parameters[c]
		splitrule = variance<br>min.node.size = 7
	Neural network	size = 13<br>decay = 1e-04
	Support vector machines, radial kernel	sigma = 0.0169<br>C = 0.2
	Gradient boosted machines	n.trees = 100<br>interaction.depth = 1<br>shrinkage = 0.1<br>n.minobsinnode = 5
FVC	Random Forest	mtry = 2<br>splitrule = variance<br>min.node.size = 5
	Neural network	size = 1<br>decay = 1e-04
	Support vector machines, radial kernel	sigma = 0.0169<br>C = 0.1
	Gradient boosted machines	n.trees = 50<br>interaction.depth = 1<br>shrinkage = 0.1<br>n.minobsinnode = 5
FEV1	Random Forest	mtry = 2<br>splitrule = variance<br>min.node.size = 3
	Neural network	size = 1<br>decay = 1e-05
	Support vector machines, radial kernel	sigma = 0.0169<br>C = 0.1
	Gradient boosted machines	n.trees = 50<br>interaction.depth = 1<br>shrinkage = 0.1<br>n.minobsinnode = 5

[a]LFT: lung function testing; DLCO: diffusion capacity for carbon monoxide; FVC: forced vital capacity; FEV1: forced expiratory volume in one second.

[b]Tuning criteria in participant-blocked 10-repeats cross-validation: maximum of Youden's J for DLCO < 80%, FVC < 80%, FEV1 < 80%; minimum of mean absolute error for DLCO, FVC, FEV1.

Response[ab]	Algorithm	Parameters[c]

[c]mtry: number of randomly chosen explanatory variables for construction of decision trees; splitrule: rule used for splitting of explanatory variable at branches of decision trees; min.node.size: minimal size of the decision tree node; size: number of neurons in the hidden layer of the neuronal network; decay: decay of weights in the neuronal network; sigma: sigma parameter that defines width of the radial kernel; C: cost/penalty applied to misclassified observations; n.tree: number of decision trees; interaction.depth: number of splits in the decision tree; shrinkage: regularization penalty applied to predictions by single decision trees; n.minobsinnode: minimal size of the decision tree node.

Supplementary Table S8: Performance of binary machine learning classifiers at predicting lung function testing (LFT) abnormalities in the entire data set.

Response[a]	Algorithm[b]	Overall accuracy[c]	κ[d]	Brier score	AUC[e]	Sensitivity	Specificity
DLCO < 80%	Random Forest	1.00	1.00	0.014000	1.00	1.00	1.00
	Neural network	1.00	1.00	0.000043	1.00	1.00	1.00
	SVM radial	0.90	0.72	0.066000	0.96	0.77	0.94
	GBM	0.98	0.94	0.019000	1.00	0.94	0.99
FVC < 80%	Random Forest	0.99	0.96	0.024000	1.00	0.95	1.00
	Neural network	0.92	0.76	0.053000	0.95	0.82	0.95
	SVM radial	0.92	0.71	0.059000	0.97	0.63	0.99
	GBM	0.95	0.82	0.048000	0.98	0.78	0.99
FEV1 < 80%	Random Forest	1.00	1.00	0.016000	1.00	1.00	1.00
	Neural network	0.93	0.78	0.049000	0.96	0.86	0.95
	SVM radial	0.92	0.67	0.061000	0.98	0.56	1.00
	GBM	0.90	0.57	0.084000	0.91	0.47	0.99

[a]LFT: lung function testing, DLCO: diffusion capacity for carbon monoxide, FVC: forced vital capacity; FEV1: forced expiratory volume in one second.

[b]SVM: support vector machines with radial kernel; GBM: gradient boosted machines.

[c]Ratio of correct predictions to the total observation number.

[d]Cohen κ statistic of inter-rater reliability between the predicted and observed outcome.

[e]AUC: are under the receiver-operating characteristic curve.

Supplementary Table S9: Performance of regression machine learning models at predicting values of lung function testing parameters in the entire data set.

Response[a]	Algorithm[b]	Data set[c]	pseudo-R^{2d}	MAE[e]	ρ
DLCO	Random Forest	training	0.8700	5.1	0.95
	Neural network	training	0.3600	12.0	0.54
	SVM radial	training	0.5500	9.4	0.77
	GBM	training	0.6200	9.3	0.74
FVC	Random Forest	training	0.6900	5.8	0.91
	Neural network	training	0.0024	10.0	
	SVM radial	training	0.2200	8.9	0.57
	GBM	training	0.2800	8.9	0.50
FEV1	Random Forest	training	0.7400	5.9	0.94
	Neural network	training	0.0024	12.0	
	SVM radial	training	0.2100	9.8	0.59
	GBM	training	0.3000	9.9	0.54

[a]DLCO: diffusion capacity for carbon monoxide, FVC: forced vital capacity; FEV1: forced expiratory volume in one second.

[b]SVM: support vector machines with radial kernel; GBM: gradient boosted machines.

[c]Defined as 1 - ratio of mean squared error and variance.

[d]MAE: mean absolute error.

[e]P: Spearman coefficient of correlation between the predicted and observed response values.

Supplementary Table S10: Mean values of SHAP (Shapley additive explanations) variable importance statistic for the models of reduced diffusion capacity for carbon monoxide and of diffusion capacity for carbon monoxide < 80% of reference. The table is available as a supplementary Excel file.

Supplementary Table S11: Correlations between chest computed tomography severity score, and AI-determined opacity and high opacity.

Variable 1[a]	Variable 2[a]	N	Correlation coefficient[b]	Significance[b]
CTSS	opacity, AI	420	ρ = 0.83 [0.78 - 0.88]	p < 0.001
CTSS	high opacity, AI	420	ρ = 0.73 [0.65 - 0.79]	p < 0.001
opacity, AI	high opacity, AI	420	ρ = 0.86 [0.83 - 0.89]	p < 0.001

[a]CTSS: CT severity score determined by a human radiologist, sum for al lobes; opacity and high opacity, AI: opacity and high opacity of the lungs determined by artificial intelligence.

[b]Blocked bootstrap Spearman correlation test; ρ correlation coefficients with 95% confidence intervals. P values were corrected for multiple testing with the false discovery rate method.

Supplementary Table S12: Differences in chest computed tomography severity score (CTSS), and AI-determined lung opacity and high opacity in CovILD study participants with and without ground glass opacity (GGO) and reticulation. Numeric variables are presented as medians with interquartile ranges (IQR) and ranges.

CT abnormality[a]	Variable[b]	Abnormality absent	Abnormality present	Significance[c]	Effect size[c]
GGO	Observations, n	187	233		
	CTSS, points	0 [IQR: 0 - 0]<br>range: 0 - 15<br>complete: n = 187	7 [IQR: 3 - 12]<br>range: 0 - 20<br>complete: n = 233	p < 0.001	r = 0.92
	opacity, AI, % of lung	0 [IQR: 0 - 0.01]<br>range: 0 - 4.8<br>complete: n = 187	0.53 [IQR: 0.05 - 2.6]<br>range: 0 - 37<br>complete: n = 233	p < 0.001	r = 0.75
	high opacity, AI, % of lung	0 [IQR: 0 - 0]<br>range: 0 - 1<br>complete: n = 187	0.012 [IQR: 0 - 0.091]<br>range: 0 - 3.1<br>complete: n = 233	p < 0.001	r = 0.55
reticulation	Observations, n	213	207		
	CTSS, points	0 [IQR: 0 - 0]<br>range: 0 - 15<br>complete: n = 213	7 [IQR: 3 - 13]<br>range: 1 - 20<br>complete: n = 207	p < 0.001	r = 0.87
	opacity, AI, % of lung	0 [IQR: 0 - 0.015]<br>range: 0 - 18<br>complete: n = 213	0.59 [IQR: 0.08 - 3]<br>range: 0 - 37<br>complete: n = 207	p < 0.001	r = 0.78
	high opacity, AI, % of lung	0 [IQR: 0 - 0]<br>range: 0 - 2.4<br>complete: n = 213	0.019 [IQR: 0 - 0.11]<br>range: 0 - 3.1<br>complete: n = 207	p < 0.001	r = 0.61

[a]LFT: lung function testing; DLCO: diffusion capacity for carbon monoxide; FVC: forced vital capacity; FEV1: forced expiratory volume in one second.

[b]CTSS: CT severity score determined by a human radiologist, sum for all lobes; opacity and high opacity, AI: opacity and high opacity of the lungs determined by artificial intelligence.

[c]Blocked bootstrap test with biserial r effect size statistic. P values corrected for multiple testing with the false discovery rate method.

Supplementary Table S13: Differences in chest computed tomography severity score (CTSS), and AI-determined lung opacity and high opacity in CovILD study participants with and without lung function testing (LFT) abnormalities. Numeric variables are presented as medians with interquartile ranges (IQR) and ranges.

LFT abnormality[a]	Variable[b]	Abnormality absent	Abnormality present	Significance[c]	Effect size[c]
DLCO < 80%	Observations, n	326	94		
	CTSS, points	1 [IQR: 0 - 5]<br>range: 0 - 20<br>complete: n = 326	10 [IQR: 4 - 15]<br>range: 0 - 20<br>complete: n = 94	p < 0.001	r = 0.57
	opacity, AI, % of lung	0.01 [IQR: 0 - 0.31]<br>range: 0 - 22<br>complete: n = 326	1.3 [IQR: 0.19 - 5.3]<br>range: 0 - 37<br>complete: n = 94	p < 0.001	r = 0.63
	high opacity, AI, % of lung	0 [IQR: 0 - 0.01]<br>range: 0 - 2.4<br>complete: n = 326	0.063 [IQR: 0.0075 - 0.38]<br>range: 0 - 3.1<br>complete: n = 94	p < 0.001	r = 0.58
FVC < 80%	Observations, n	337	83		
	CTSS, points	2 [IQR: 0 - 7]<br>range: 0 - 20<br>complete: n = 337	5 [IQR: 0 - 12]<br>range: 0 - 20<br>complete: n = 83	ns (p = 0.092)	r = 0.21
	opacity, AI, % of lung	0.03 [IQR: 0 - 0.53]<br>range: 0 - 31<br>complete: n = 337	0.43 [IQR: 5e-04 - 2.7]<br>range: 0 - 37<br>complete: n = 83	p = 0.0051	r = 0.29
	high opacity, AI, % of lung	0 [IQR: 0 - 0.017]<br>range: 0 - 2<br>complete: n = 337	0.01 [IQR: 0 - 0.19]<br>range: 0 - 3.1<br>complete: n = 83	ns (p = 0.062)	r = 0.3
FEV1 < 80%	Observations, n	343	77		
	CTSS, points	2 [IQR: 0 - 7]<br>range: 0 - 20<br>complete: n = 343	4 [IQR: 0 - 12]<br>range: 0 - 20<br>complete: n = 77	ns (p = 0.11)	r = 0.2
	opacity, AI, % of lung	0.03 [IQR: 0 - 0.55]<br>range: 0 - 31<br>complete: n = 343	0.33 [IQR: 0.001 - 2.5]<br>range: 0 - 37<br>complete: n = 77	p = 0.009	r = 0.27
	high opacity, AI, % of lung	0 [IQR: 0 - 0.017]<br>range: 0 - 3.1<br>complete: n = 343	0.016 [IQR: 0 - 0.2]<br>range: 0 - 2.5<br>complete: n = 77	ns (p = 0.062)	r = 0.3

[a]LFT: lung function testing; DLCO: diffusion capacity for carbon monoxide; FVC: forced vital capacity; FEV1: forced expiratory volume in

LFT abnormality[a]	Variable[b]	Abnormality absent	Abnormality present	Significance[c]	Effect size[c]
one second.					
[b]CTSS: CT severity score determined by a human radiologist, sum for all lobes; opacity and high opacity, AI: opacity and high opacity of the lungs determined by artificial intelligence.					
[c]Blocked bootstrap test with biserial r effect size statistic. P values corrected for multiple testing with the false discovery rate method.					

Supplementary Table S14: Correlation of LFT variables with chest computed tomography severity score, and AI-determined opacity and high opacity.

LFT variable[a]	CT variable[b]	N	Correlation coeffcient[c]	Significance[c]
DLCO	CTSS	420	ρ = -0.44 [-0.56 - -0.3]	$p < 0.001$
	opacity, AI	420	ρ = -0.46 [-0.57 - -0.33]	$p < 0.001$
	high opacity, AI	420	ρ = -0.46 [-0.56 - -0.36]	$p < 0.001$
FVC	CTSS	420	ρ = -0.21 [-0.34 - -0.074]	$p = 0.0023$
	opacity, AI	420	ρ = -0.27 [-0.39 - -0.13]	$p < 0.001$
	high opacity, AI	420	ρ = -0.3 [-0.42 - -0.18]	$p < 0.001$
FEV1	CTSS	420	ρ = -0.14 [-0.28 - 0.005]	$p = 0.029$
	opacity, AI	420	ρ = -0.21 [-0.34 - -0.067]	$p = 0.0019$
	high opacity, AI	420	ρ = -0.27 [-0.39 - -0.14]	$p < 0.001$

[a]DLCO: diffusion capacity for carbon monoxide; FVC: forced vital capacity; FEV1: forced expiratory volume in one second.

[b]CTSS: CT severity score determined by a human radiologist, sum for al lobes; opacity and high opacity, AI: opacity and high opacity of the lungs determined by artificial intelligence.

[c]Blocked bootstrap Spearman correlation test; ρ correlation coefficients with 95% confidence intervals. P values were corrected for multiple testing with the false discovery rate method.

Supplementary Figures

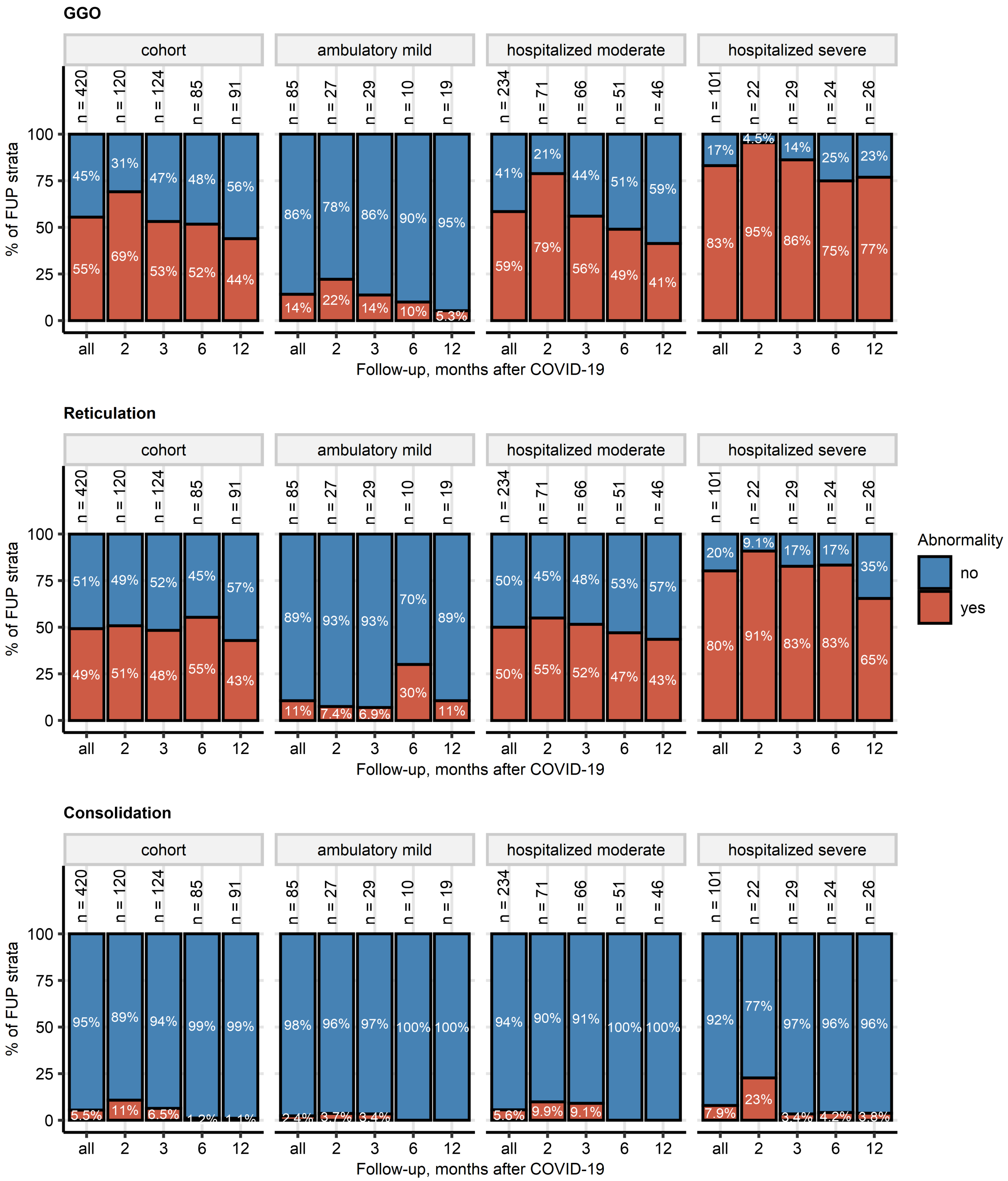

Supplementary Figure S1. Frequency of the most common abnormalities in computed tomography of the chest at the consecutive follow-ups in participants stratified by severity of acute COVID-19.

Frequencies of any chest computed tomography (CT) abnormalities, ground glass opacity (GGO), and reticulations identified by a human radiologist at the consecutive follow-ups after COVID-19 in the CovILD study patients stratified by the severity of acute COVID-19 were presented in stack plots. Numbers of complete observations at the follow-up examinations are displayed above the data bars.

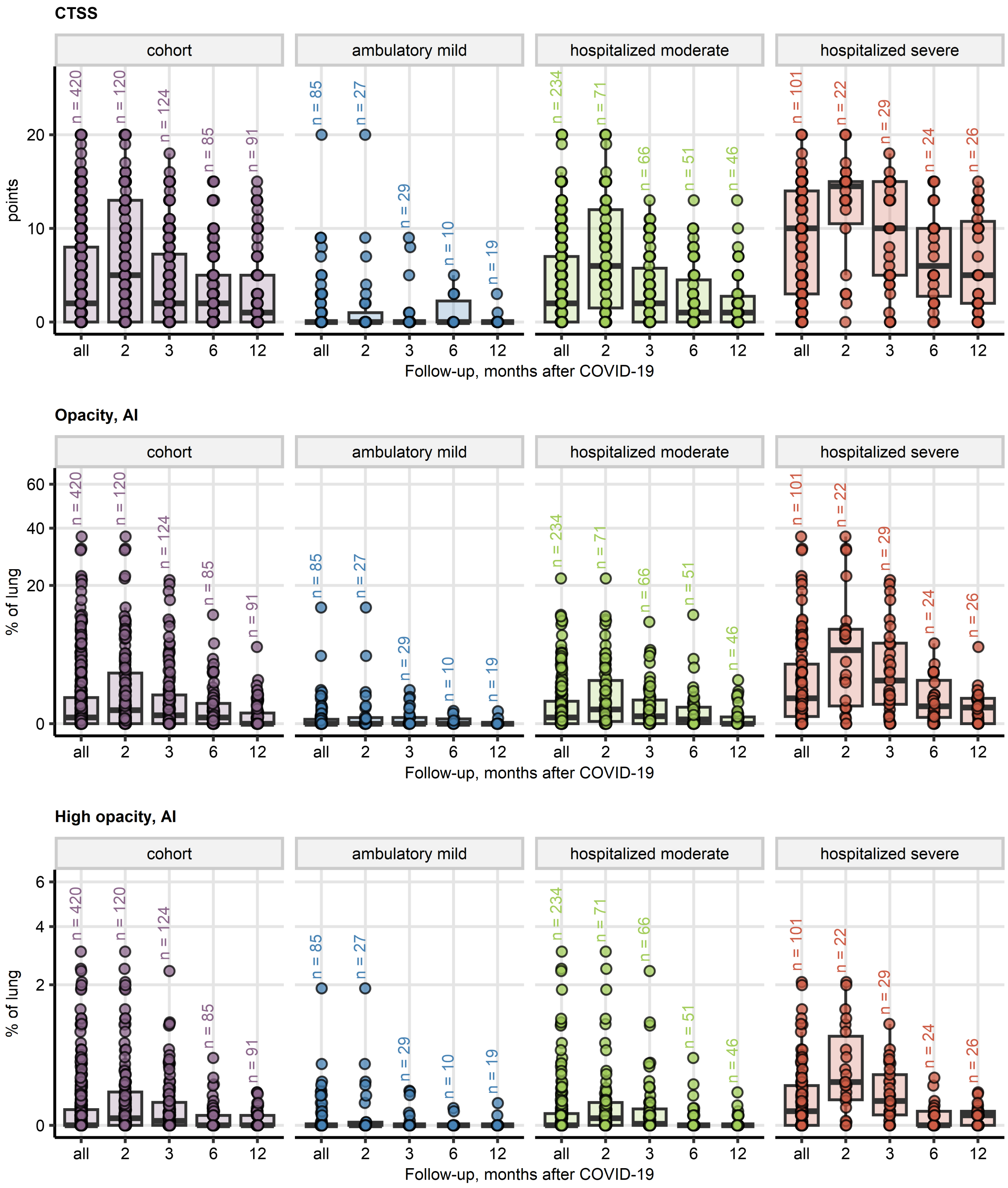

Supplementary Figure S2. Time course of numeric computed tomography readouts at the consecutive follow-ups in participants stratified by severity of acute COVID-19.

Severity of chest computed tomography (CT) abnormalities was assessed by a human-determined CT severity score (CTSS), and AI-determined percentages of the lung tissue with opacity and high opacity. Time courses of these parameters are visualized in box plots. Boxes represent medians with interquartile ranges, whiskers span over 150% of the interquartile range. Single observations are depicted as points. Numbers of complete observations at the follow-up examinations are displayed above the data points.

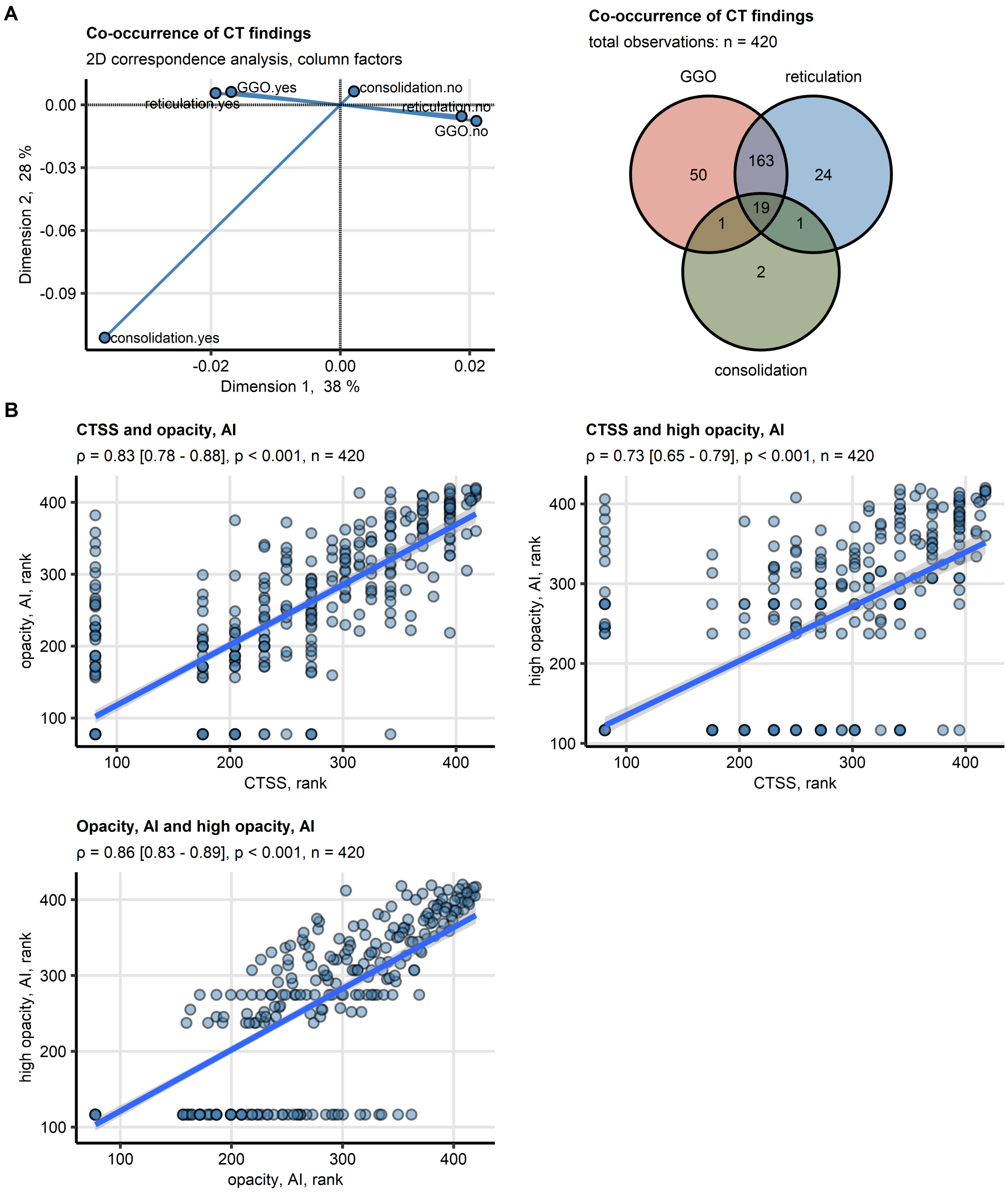

Supplementary Figure S3. Co-occurrence of abnormalities of chest computed tomography and correlation of computed tomography readouts in COVID-19 convalescents.

(A) Co-occurrence of ground glass opacity (GGO), reticulation, and consolidation diagnosed by a human radiologist was investigated by two-dimensional correspondence analysis. Left: plots of the column factors, proximity of points reflects co-occurrence of normal or pathological values of lung function testing. Right: Venn plot of frequencies of GGO, reticulation, and consolidation; the total observation number is displayed in the plot caption.

(B) Correlation between human-determined (CTSS: computed tomography severity score, sum of points form the entire lung) and artificial intelligence determined (AI, opacity and high opacity, percentage of the lung) readouts of severity of structural lung lesions was assessed by blocked bootstrap Spearman's test and visualized in scatter plots of observation ranks. Each point represents a single observation, linear trends with standard errors are depicted as blue lines with gray ribbons. Values of correlation coefficients with 95% confidence intervals and p values are displayed in the plot captions.

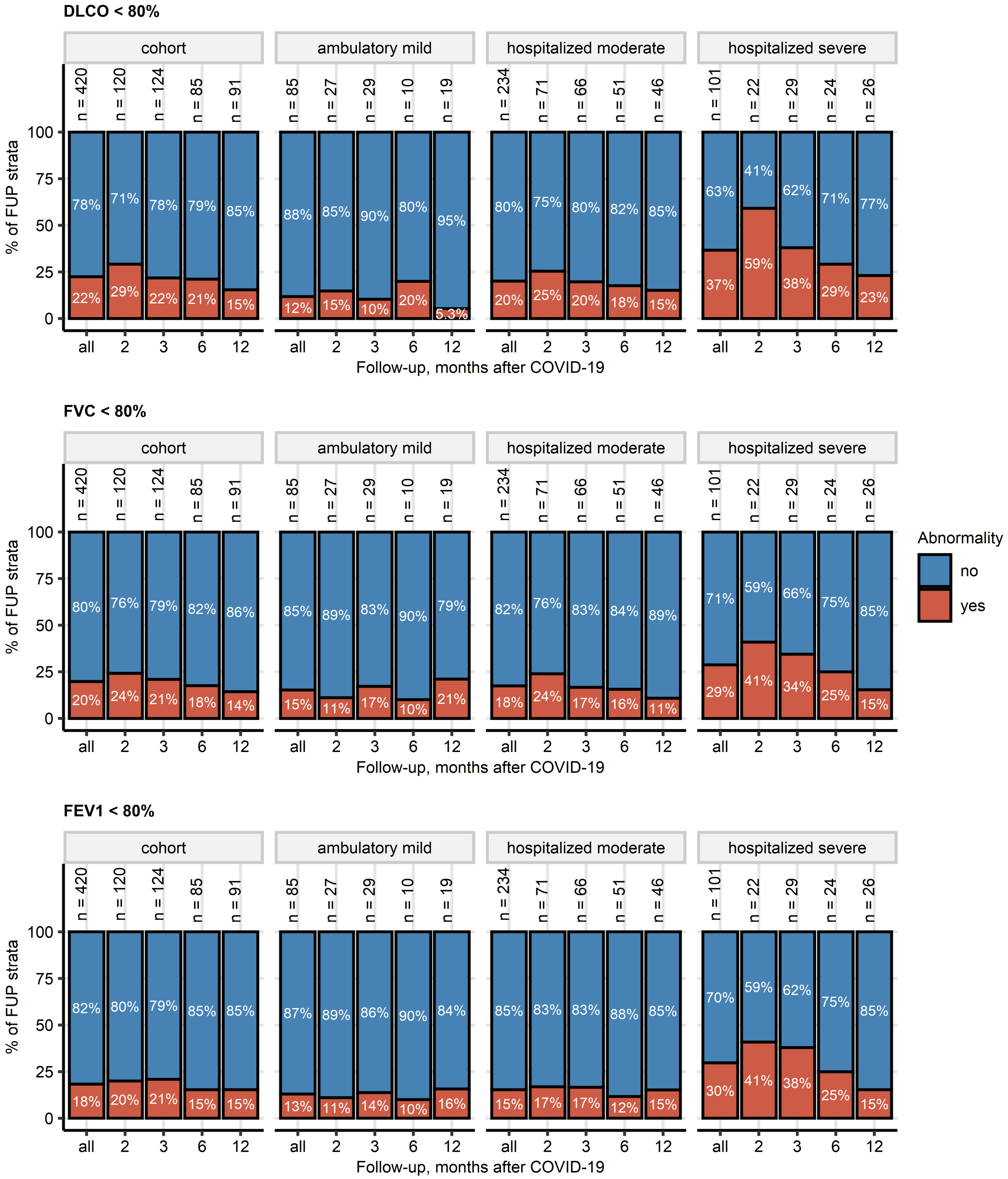

Supplementary Figure S4. Frequency of lung function testing abnormalities at the consecutive follow-ups in participants stratified by severity of acute COVID-19.

Insufficient diffusion capacity for carbon monoxide (DLCO), forced vital capacity (FVC), and forced expiratory volume in one second (FEV1) was defined as values < 80% of the individual's reference. Frequencies of these abnormalities in in the CovILD study patients stratified by the severity of acute COVID-19 were presented in stack plots. Numbers of complete observations at the follow-up examinations are displayed above the data bars.

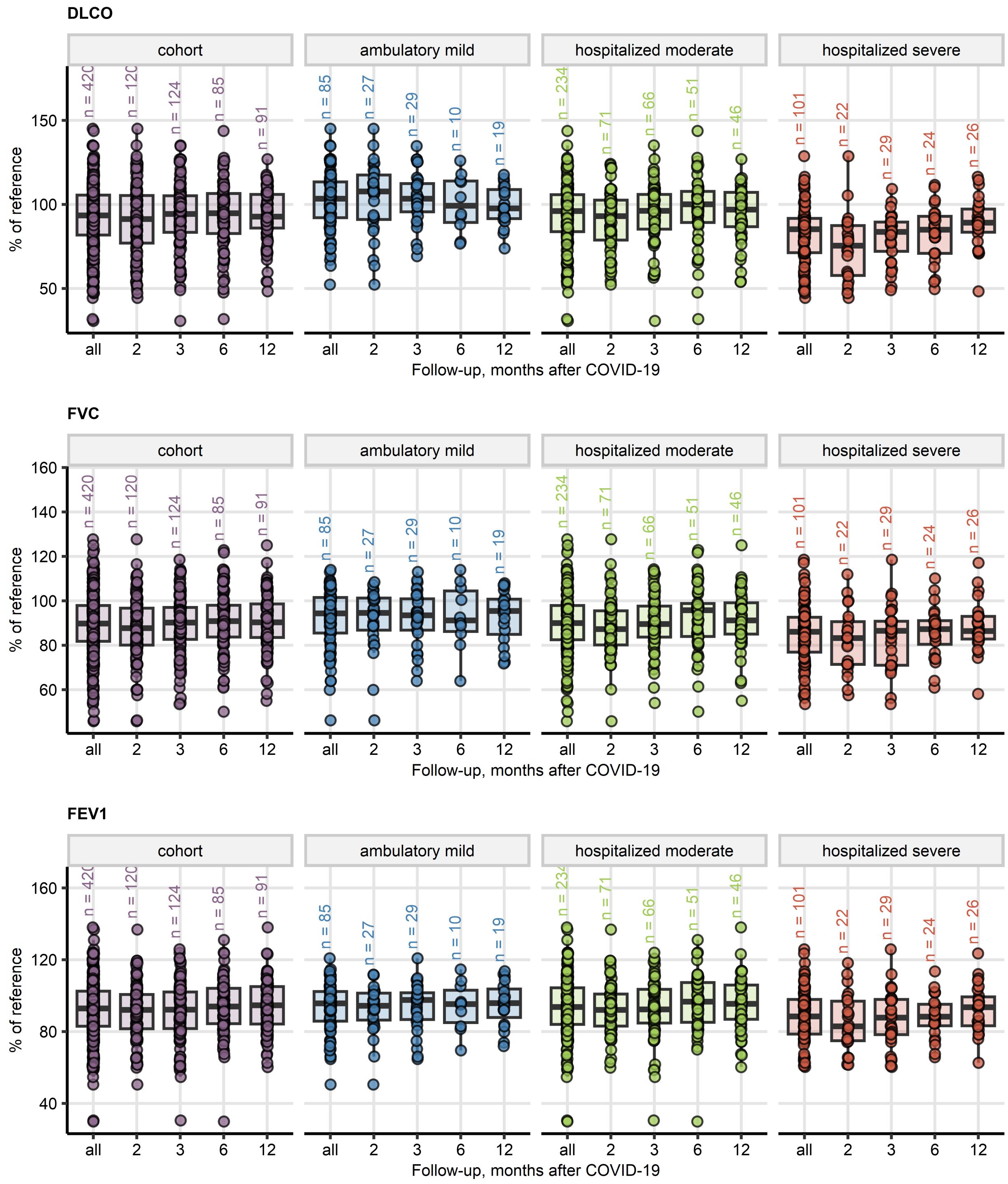

Supplementary Figure S5. Time course of numeric lung function testing readouts at the consecutive follow-ups in participants stratified by severity of acute COVID-19.

Diffusion capacity for carbon monoxide (DLCO), forced vital capacity (FVC), forced expiratory volume in one second (FEV1) were recorded a percentages of the patient's reference values. Time courses of these parameters are visualized in box plots. Boxes represent medians with interquartile ranges, whiskers span over 150% of the interquartile range. Single observations are depicted as points. Numbers of complete observations at the follow-up examinations are displayed above the data points.

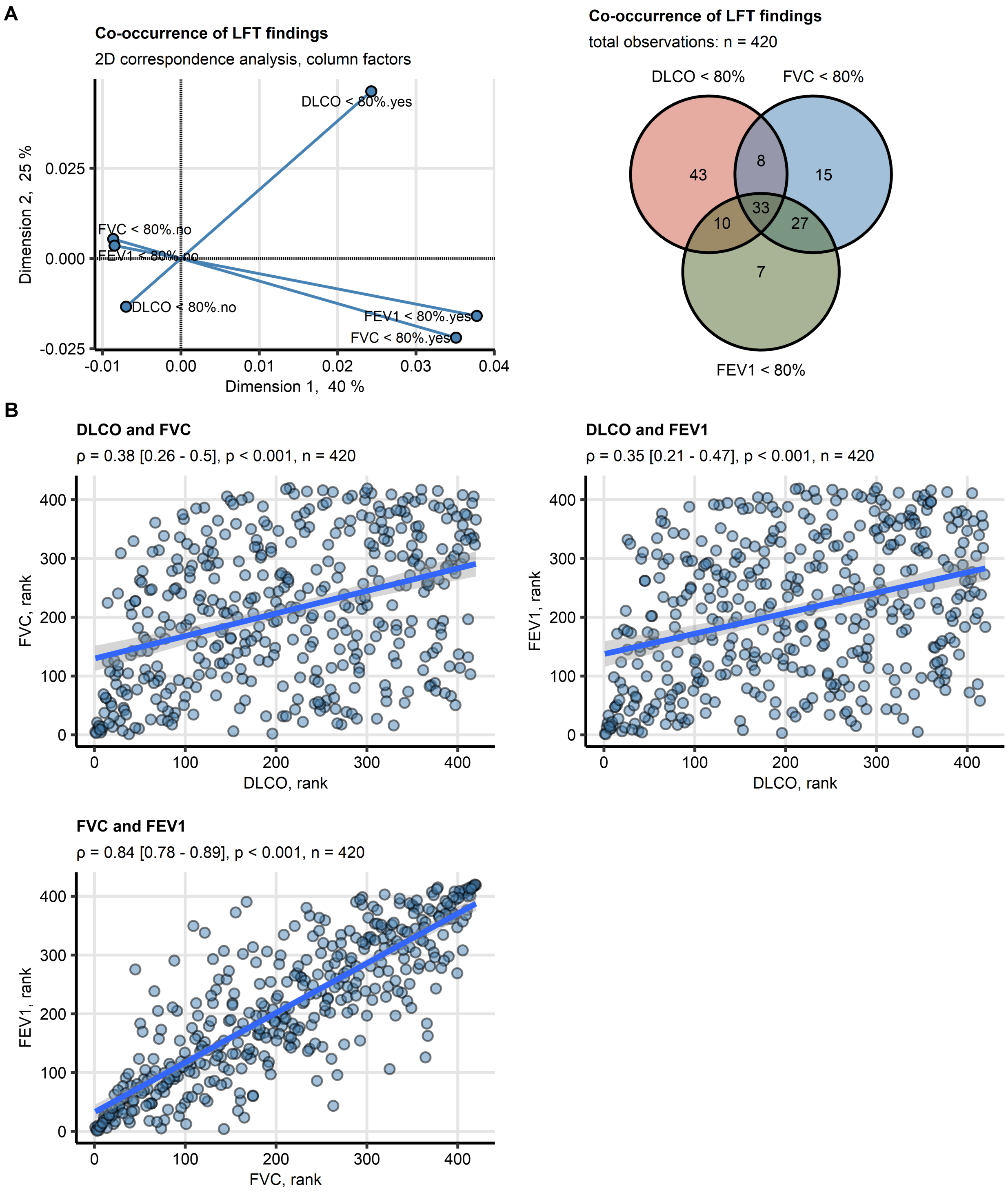

Supplementary Figure S6. Co-occurrence of abnormalities of lung function testing and correlation of lung function readouts in COVID-19 convalescents.

(A) Co-occurrence of insufficient diffusion capacity for carbon monoxide (DLCO < 80% of reference value), insufficient forced vital capacity (FVC < 80% of reference), and insufficient forced expiratory volume in one second (FEV1 < 80% of reference) was investigated by two-dimensional correspondence analysis. Left: plots of the column factors, proximity of points reflects co-occurrence of normal or pathological values of lung function testing. Right: Venn plot of frequencies of insufficient DLCO, FVC, and FEV1; the total observation number is displayed in the plot caption.

(B) Correlation between the lung function testing parameters was assessed by blocked bootstrap Spearman's test and visualized in scatter plots of observation ranks. Each point represents a single observation, linear trends with standard errors are depicted as blue lines with gray ribbons. Values of correlation coefficients with 95% confidence intervals and p values are displayed in the plot captions.

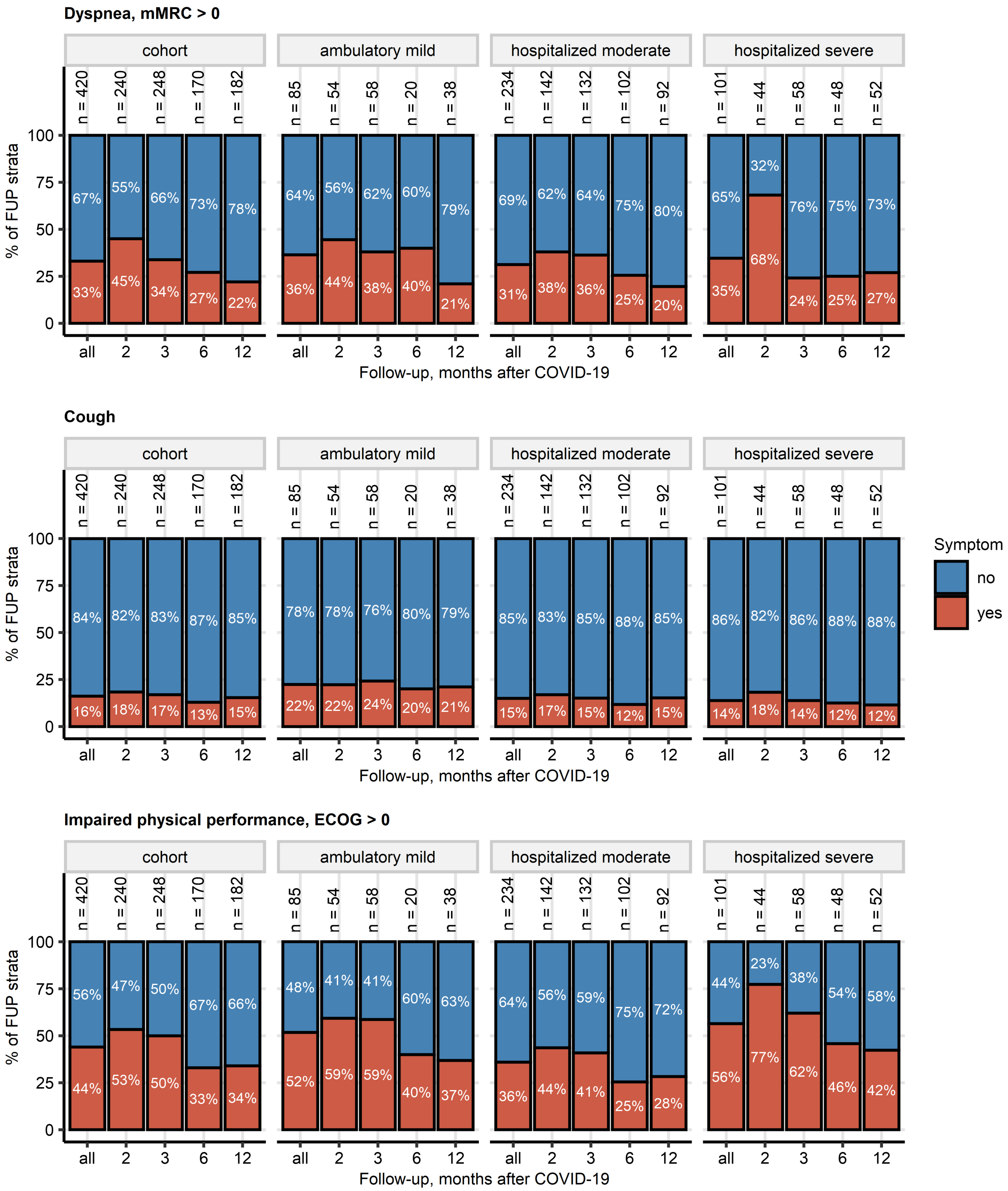

Supplementary Figure S7. Frequency of persistent symptoms of relevance for lung function at the consecutive follow-ups in participants stratified by severity of acute COVID-19.

Intensity of self-reported dyspnea was assessed with the modified Medical Research Council scale (mMRC) and presence of dyspnea was defined as mMRC > 0. Presence of cough was surveyed by a single 'yes'/'no' item. Impairment of physical performance was assessed with the Eastern Cooperative Oncology Group scale (ECOG, high values indicate more profound impairment) and impaired physical performance was defined as ECOG > 0. Frequency of dyspnea, cough and impairment of physical performance at the consecutive follow-ups after COVID-19 in the CovILD cohort stratified by the severity of acute COVID-19 was presented in stacked bar plots. Numbers of complete observations at the follow-up examinations are displayed above the data bars.

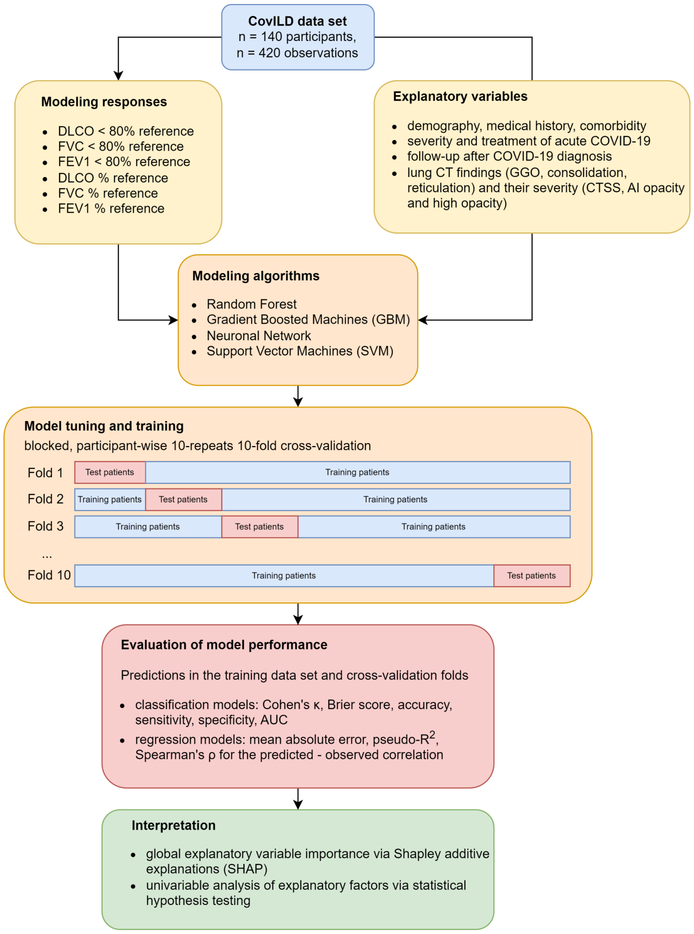

Supplementary Figure S8. Modeling strategy.

DLCO: diffusion capacity for carbon dioxide; FVC: forced vital capacity; FEV1: forced expiratory volume in 1 second; CT: computed tomography; GGO: ground glass opacity; CTSS: human-determined CT severity score; AI opacity and high opacity: artificial intelligence-determined opacity and high opacity of the lung; AUC: area under the receiver-operating characteristic curve.

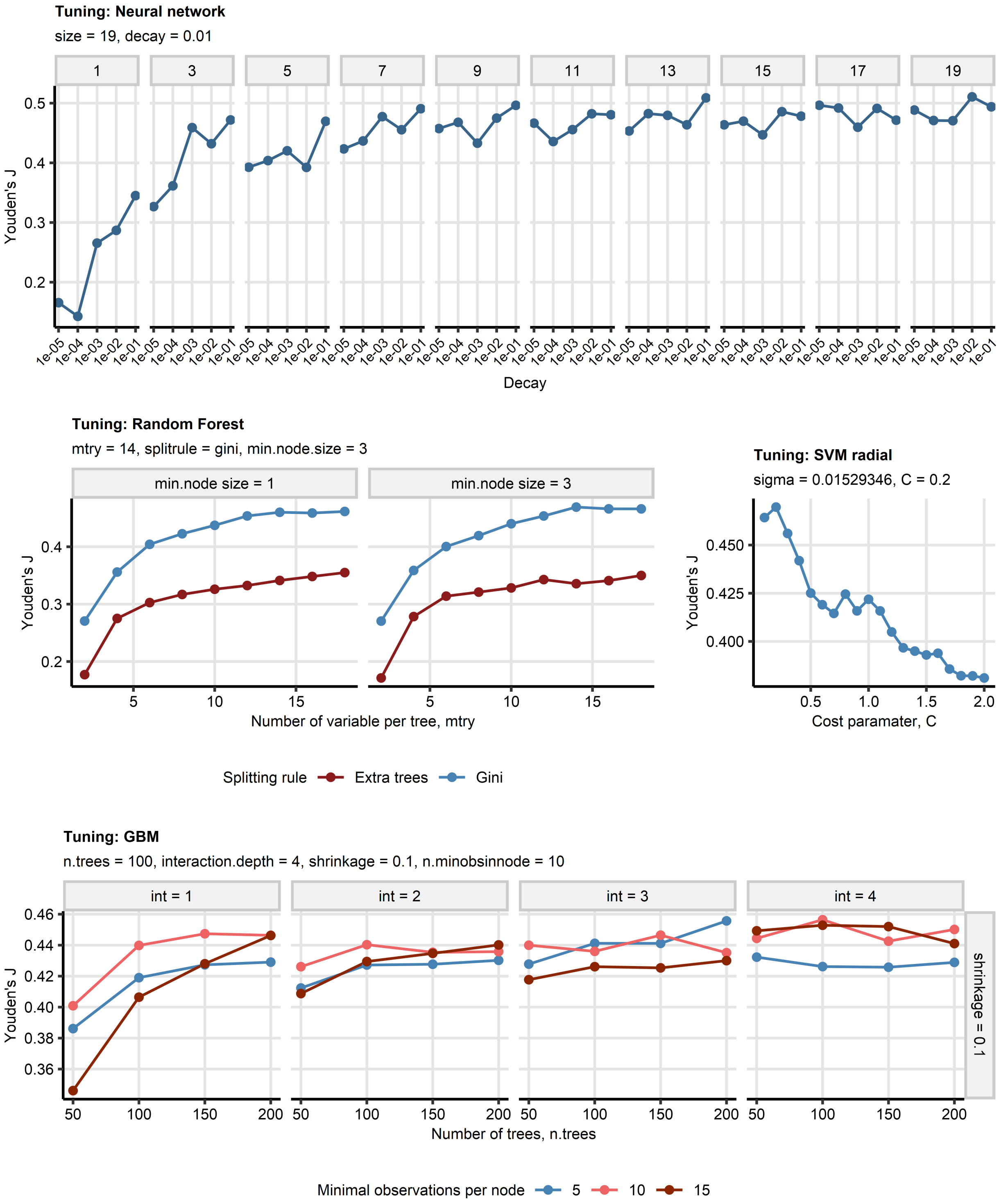

Supplementary Figure S9. Choice of hyper-parameters for machine learning classification models of reduced diffusion capacity for carbon monoxide.

Classification machine learning models of abnormalities of lung function testing (diffusion capacity of carbon monoxide [DLCO] < 80%, forced vital capacity [FVC] < 80%, forced expiratory volume in one second [FEV1] < 80% of the patient's reference) were constructed with the neural network, random forest, support vector machines with radial kernel (SVM), and gradient boosted machines (GBM) algorithms. The explanatory variables included demographic information, information on course of acute COVID-19, longitudinally recorded persistent symptoms, and longitudinally recorded readouts of structural lung damage via computed tomography of the chest. Choice of the algorithms' hyper-parameters (tuning) was accomplished by maximizing Youden's J statistic ($J = sensitivity + specificity - 1$) for out-of-fold predictions in patient-blocked 10-repeats 10-fold cross-validation. Trajectories of J values for combinations of the hyper-parameters of the models of DLCO < 80% are presented in line plots. The optimal combinations of the hyper-parameters used for training of the final models are displayed in the plot captions. The full results of tuning are presented in Supplementary Tables S6 and S7.

size: number of neurons in the hidden layer of the neuronal model; decay: decay of weights in the neuronal network; mtry: number of randomly chosen explanatory variables for construction of decision trees; splitrule: rule used for splitting of explanatory variable at branches of decision trees; min.node.size: minimal size of the decision tree node; sigma: sigma parameter that defines width of the radial kernel; C: cost/penalty applied to misclassified observations; n.tree: number of decision trees; interaction.depth/int: number of splits in the decision tree; shrinkage: regularization penalty applied to predictions by single decision trees; n.minobsinnode: minimal size of the decision tree node.

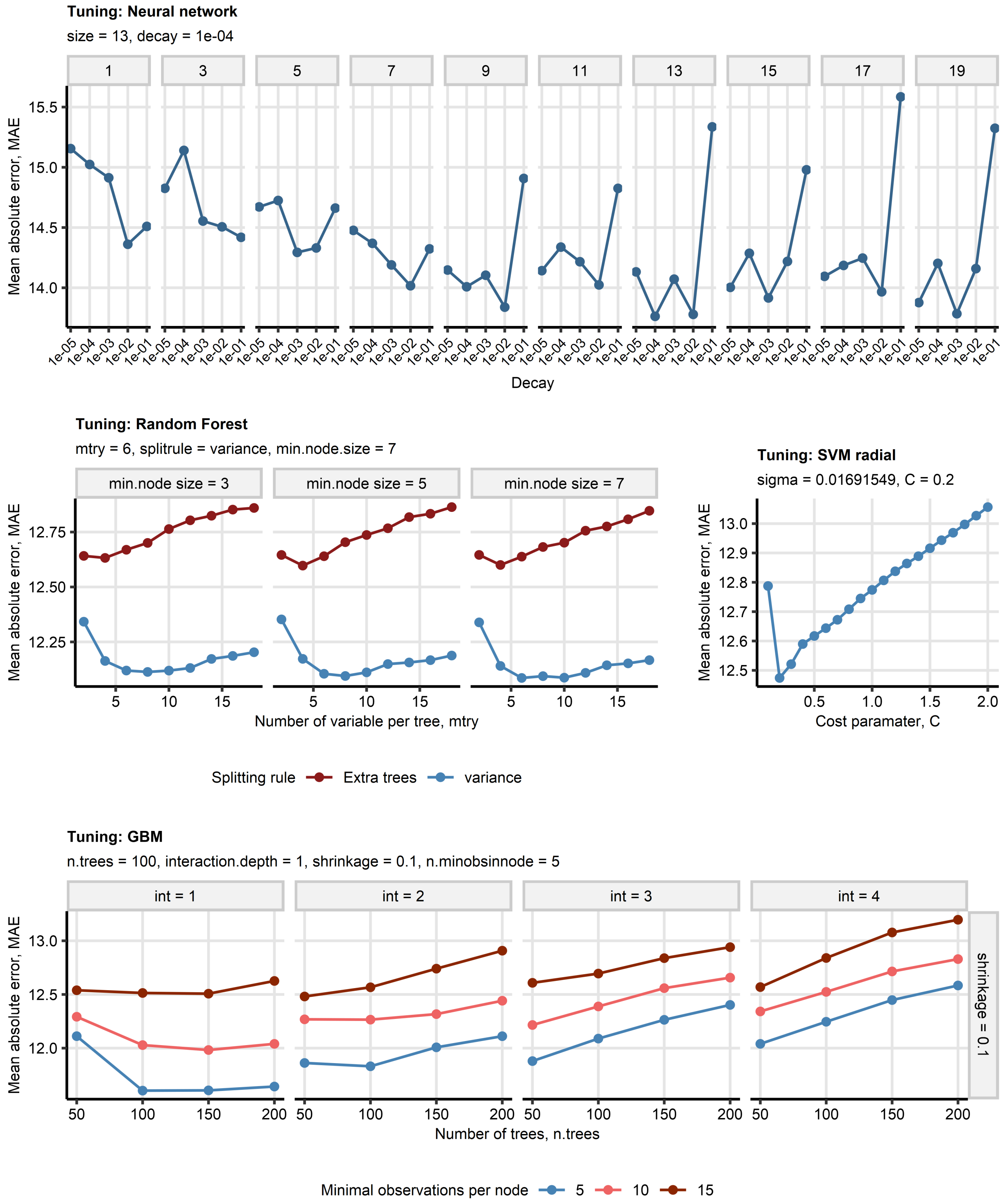

Supplementary Figure S10. Choice of hyper-parameters for machine learning regression models of diffusion capacity for carbon monoxide.

Regression machine learning models of parameters of lung function testing (diffusion capacity of carbon monoxide [DLCO], forced vital capacity [FVC], forced expiratory volume in one second [FEV1]) were constructed with the neural network, random forest, support vector machines with radial kernel (SVM), and gradient boosted machines (GBM) algorithms. The explanatory variables included demographic information, information on course of acute COVID-19, longitudinally recorded persistent symptoms, and longitudinally recorded readouts of structural lung damage via computed tomography of the chest. Choice of the algorithms' hyper-parameters (tuning) was accomplished by minimizing mean absolute errors (MAE) for out-of-fold predictions in patient-blocked 10-repeats 10-fold cross-validation. Trajectories of MAE values for combinations of the hyper-parameters of the models of DLCO are presented in line plots. The optimal combinations of the hyper-parameters used for training of the final models are displayed in the plot captions. The full results of tuning are presented in Supplementary Tables S6 and S7.

size: number of neurons in the hidden layer of the neuronal model; decay: decay of weights in the neuronal network; mtry: number of randomly chosen explanatory variables for construction of decision trees; splitrule: rule used for splitting of explanatory variable at branches of decision trees; min.node.size: minimal size of the decision tree node; sigma: sigma parameter that defines width of the radial kernel; C: cost/penalty applied to misclassified observations; n.tree: number of decision trees; interaction.depth/int: number of splits in the decision tree; shrinkage: regularization penalty applied to predictions by single decision trees; n.minobsinnode: minimal size of the decision tree node.

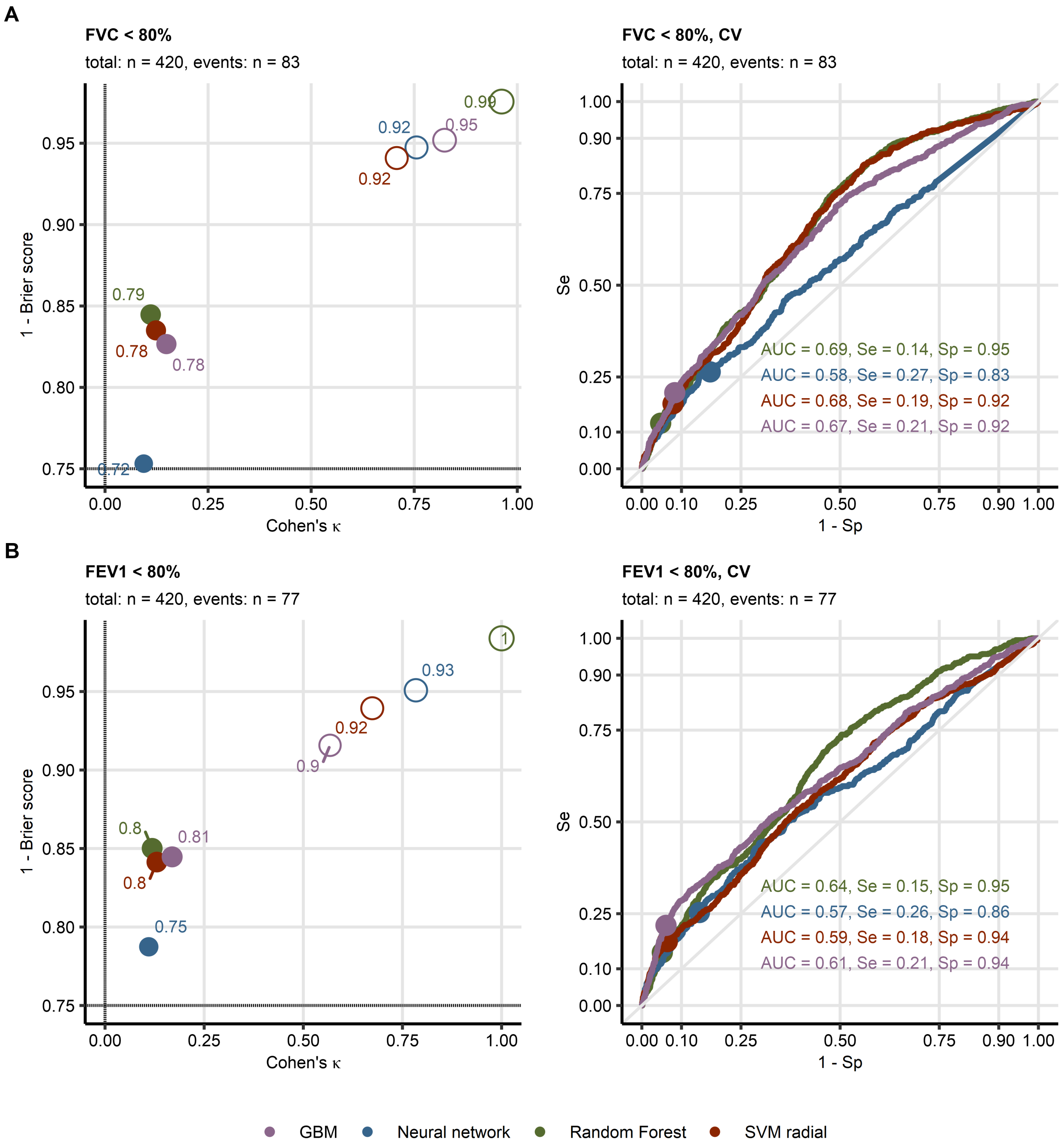

Supplementary Figure S11. Evaluation of performance of machine learning classification models at prediction of insufficient forced vital capacity and forced expiratory volume in one second.

Machine learning classification models of insufficient forced vital capacity (A, FVC < 80% of reference value: n = 83, total observations: n = 420) and of insufficient forced expiratory

volume in one second (B, FEV1 < 80% of reference value: n = 77, total observations: n = 420) employing computed tomography readouts, demographic and clinical explanatory variables were trained. The model performance was evaluated in the entire data set and 10-repeats 10-fold cross-validation with overall accuracy metric, Cohen's κ as a measure of concordance between predicted and observed outcome, and Brier score as a measure of model's calibration. Left: numeric performance measures of the models (open circles: the entire data set, filled circles: cross-validation); point sizes and point labels represent overall model accuracy, the dashed lines visualize values of Cohen's κ and Brier score expected for prediction of insufficient DLCO be chance. Right: receiver-operating characteristic curves for predictions in cross-validation folds, numeric statistics are displayed in the plot. Numbers of complete observations and observations with the particular LFT insufficiency ('events') are displayed in the plot captions.

FVC: forced vital capacity; FEV1: forced expiratory volume in one second, CV: cross-validation; AUC: are under the receiver-operating characteristic curve; Se: sensitivity; Sp: specificity; GBM: gradient boosted machines; SVM radial: support vector machines with radial kernel.

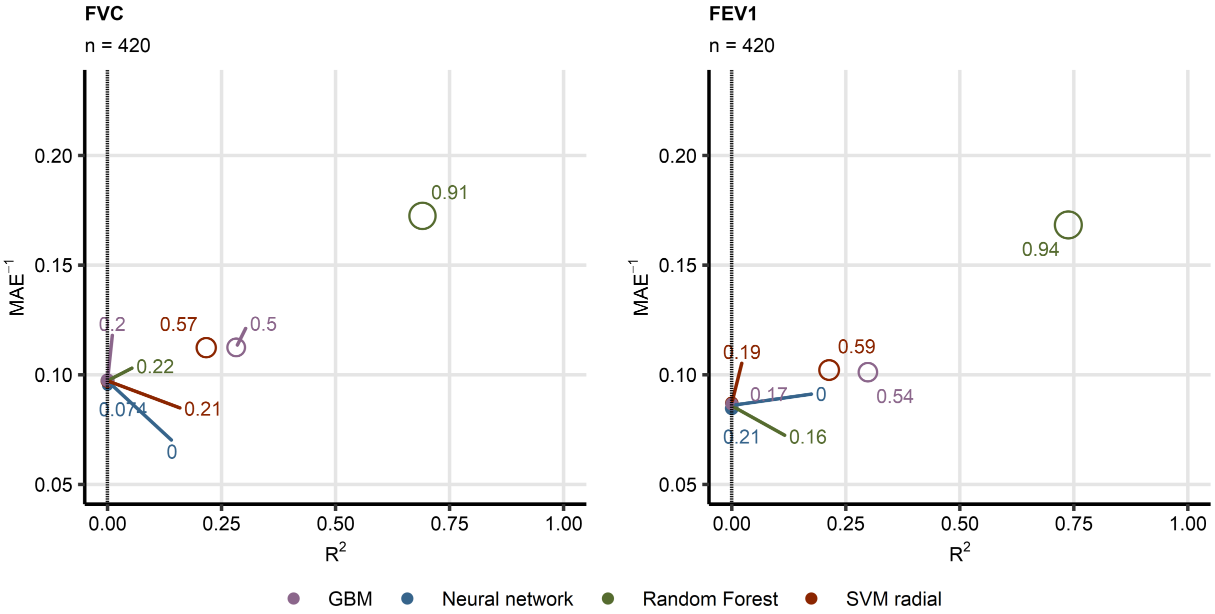

Supplementary Figure S12. Evaluation of performance of machine learning regression models at prediction of forced vital capacity and forced expiratory volume in one second.

Machine learning regression models of forced vital capacity (FVC, total observations: n = 420) and of forced expiratory volume in one second (FEV1, total observations: n = 420) employing computed tomography readouts, demographic and clinical explanatory variables were trained. Their performance was evaluated in th entire data set and 10-repeats 10-fold cross-validation with R^2 *as a measure of explained variation, mean absolute error, and ρ Spearman's coefficient of correlation between the predicted and observed values. Numeric performance measures of the model are presented in bubble plots (open circles: the entire data set, filled circles: cross-validation); point sizes and point labels represent values of ρ correlation coefficient, the dashed line visualizes* R^2 *value expected for a meaningless model. Numbers of complete observations are shown in the plot captions.*

FVC: forced vital capacity; FEV1: forced expiratory volume in one second, CV: cross-validation; MAE: mean absolute error; GBM: gradient boosted machines; SVM radial: support vector machines with radial kernel.

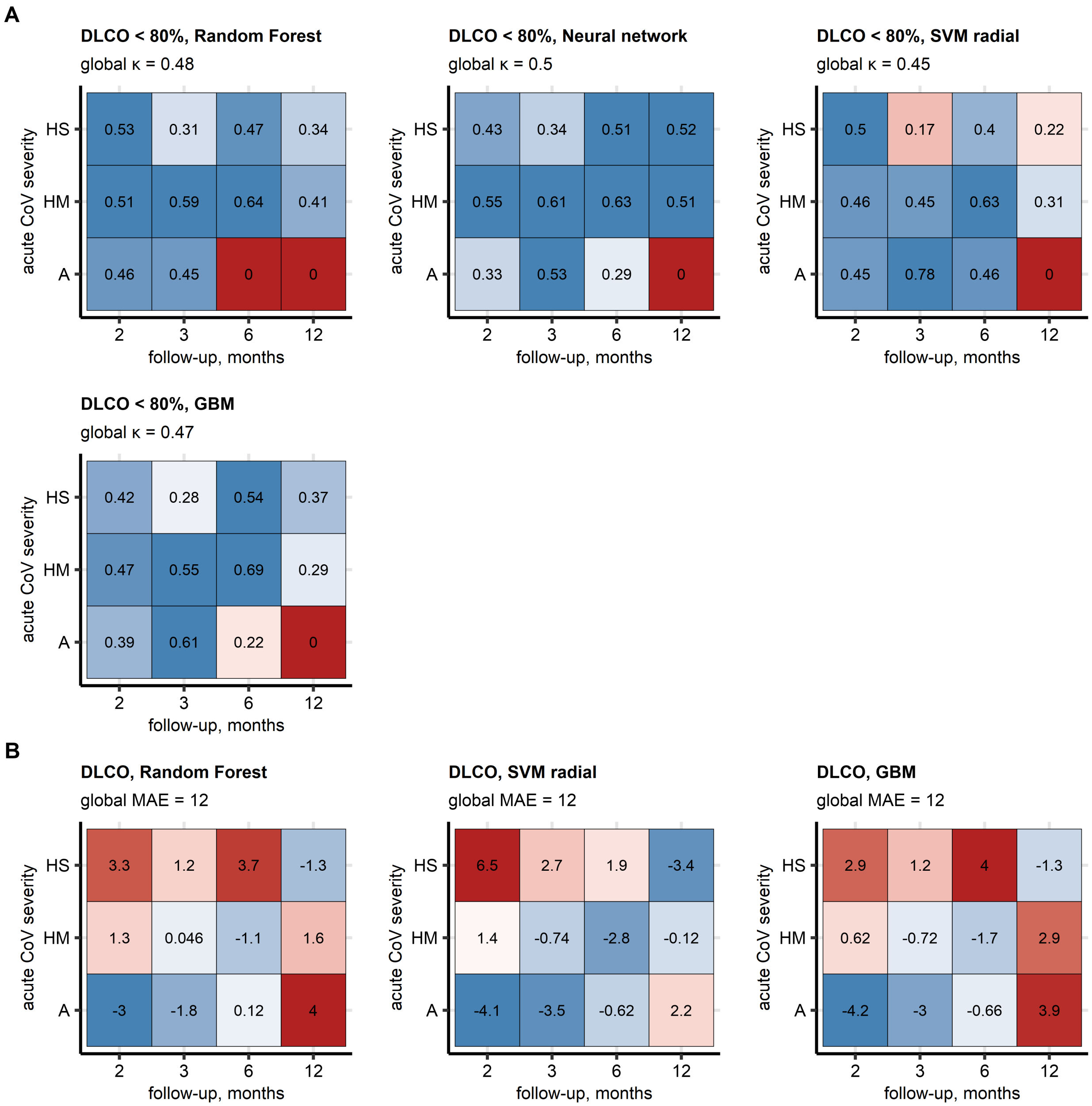

Supplementary Figure S13. Cohen's κ and mean error of the machine learning models of insufficiency and percentage of reference of diffusion capacity for carbon monoxide in observations stratified by acute COVID-19 severity and follow-up visit.

Classification machine learning models of insufficient diffusion capacity for carbon monoxide (A, DLCO < 80% of reference value: n = 94, total observations: n = 420) and regression models of diffusion capacity for carbon monoxide (DLCO, % of reference value, total observations: n = 420) were trained and evaluated as presented in Figure 2. Cohen's κ (A) and mean errors (B) of cross-validated model predictions for observations stratified by severity of acute COVID-19

and the consecutive follow-up visits were investigated. Cohen's κ and error values are visualized in heat maps; tiles are labeled with the error values. Global κ and mean absolute model errors are indicated in the plot captions.

DLCO: diffusion capacity for carbon monoxide; SVM radial: support vector machines with radial kernel; GBM: gradient boosted machines; MAE: mean absolute error; A: ambulatory COVID-19; HM: hospitalized, moderate COVID-19; HS: hospitalized, severe COVID-19..

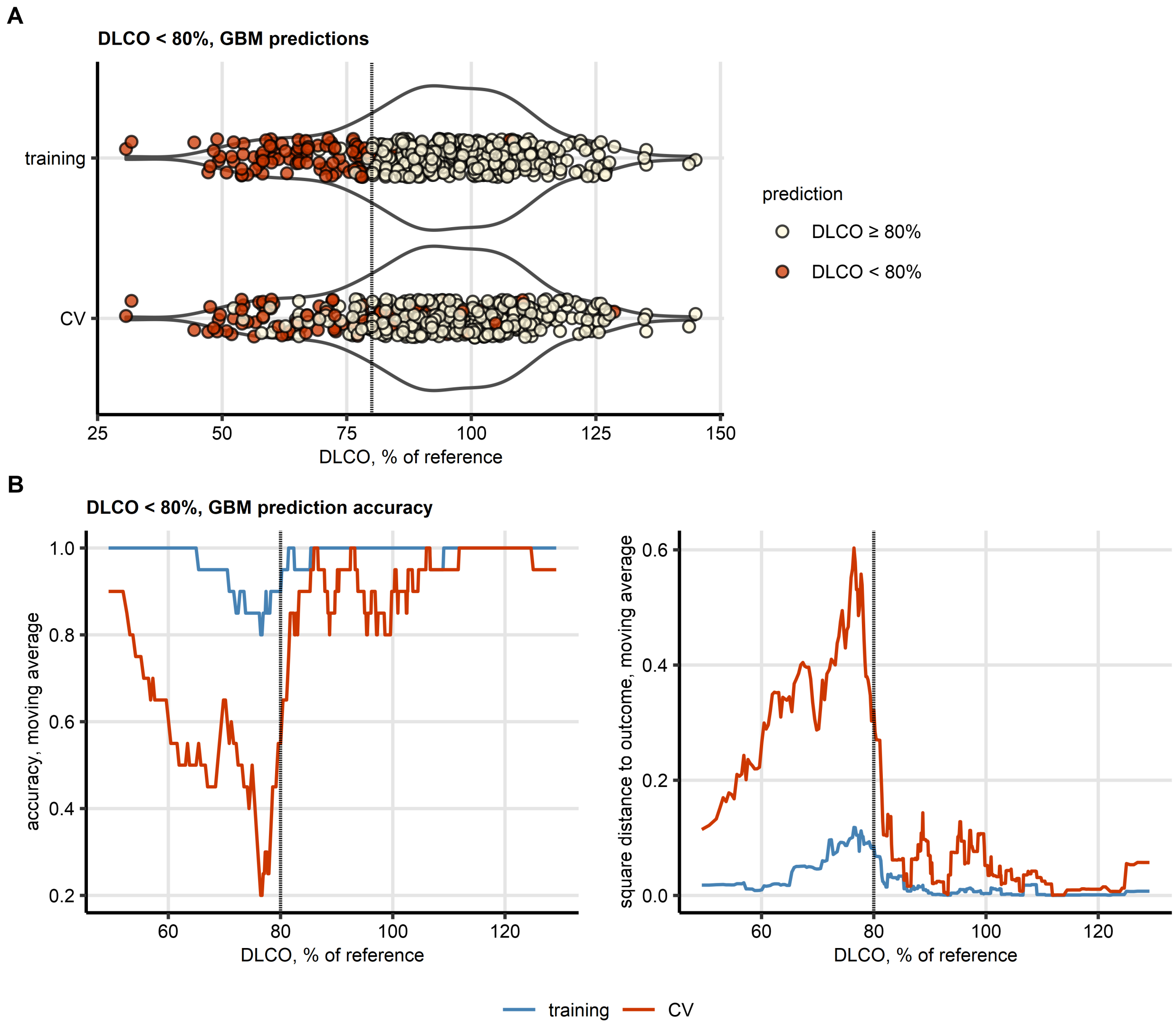

Supplementary Figure S14. Accuracy of prediction of insufficient diffusion capacity for carbon monoxide by the GBM model as a function of diffusion capacity for carbon monoxide expressed as percentage of patient's reference.

Predictions made by the best performing model of reduced diffusion capacity for carbon monoxide (DLCO < 80% of the patient's reference) were investigated in observations with increasing DLCO values expressed as percentage of the patient's reference.

(A) The actual DLCO values expressed as percentages of the patient's reference are displayed in violin plots. Single observations are depicted as points, point color codes for the predicted reduced DLCO <80% in the training data and 10-repeats 10-fold cross-validation (CV). The dashed line represents the 80% cutoff used for definition of reduced DLCO,

(B) Moving averages of accuracy (fraction of correct predictions) at prediction of DLCO < 80% and squared distances to the 0/1-coded DLCO < 80% in the training data set and 10-repeats 10-fold CV. The moving averages calculated with the sliding window of 20 observations are displayed as solid lines. The dashed lines represent the 80% cutoff used for definition of reduced DLCO.

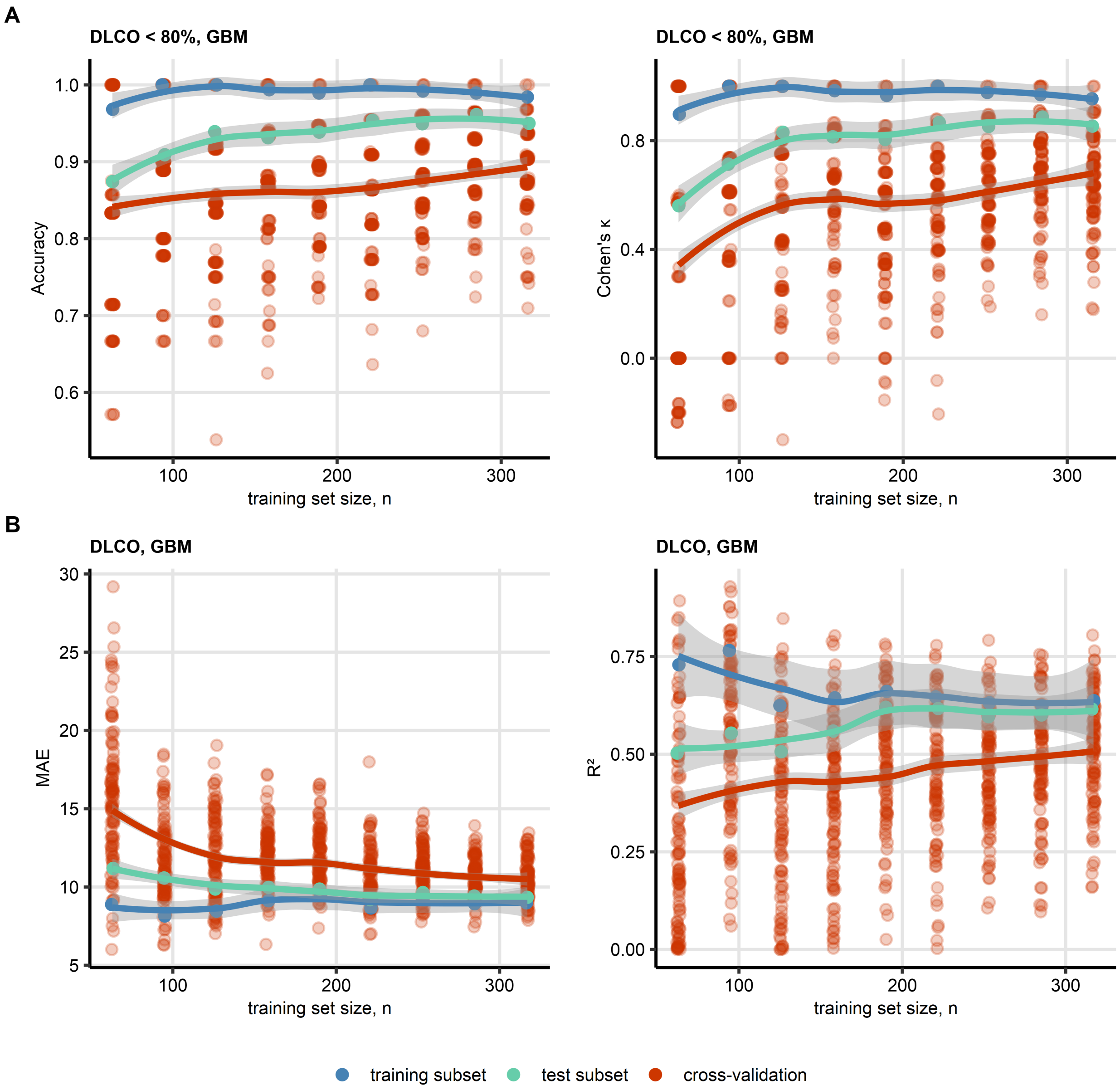

Supplementary Figure S15. Learning curves of the gradient boosted machines models of insufficiency and percentage of reference of diffusion capacity for carbon monoxide.

Gradient boosted machines (GBM) was the best performing algorithm at modeling of insufficiency of diffusion capacity for carbon monoxide (DLCO < 80% of reference) and diffusion capacity for carbon monoxide expressed as percentage of the reference value (DLCO, Figure 2). Over-fitting behavior of the GBM models was investigated by examination of learning curves, i.e. curves of model performance metrics (A, DLCO < 80%: accuracy and Cohen's κ, B, DLCO: mean absolute error [MAE] and R^2) for models trained in subsets of

observations with increasing sizes. The performance metrics were computed for the training subsets, test subsets (size of each corresponded to 25% of the training subsets) and 10-repeats 10-fold cross-validation, and were visualized in scatter plots. Each point represents a single prediction in the training or test subset, or an out-of-fold prediction in the cross-validation. Locally estimated scatterplot smoothing (LOESS) trends with standard error regions are visualized as solid lines with gray ribbons.

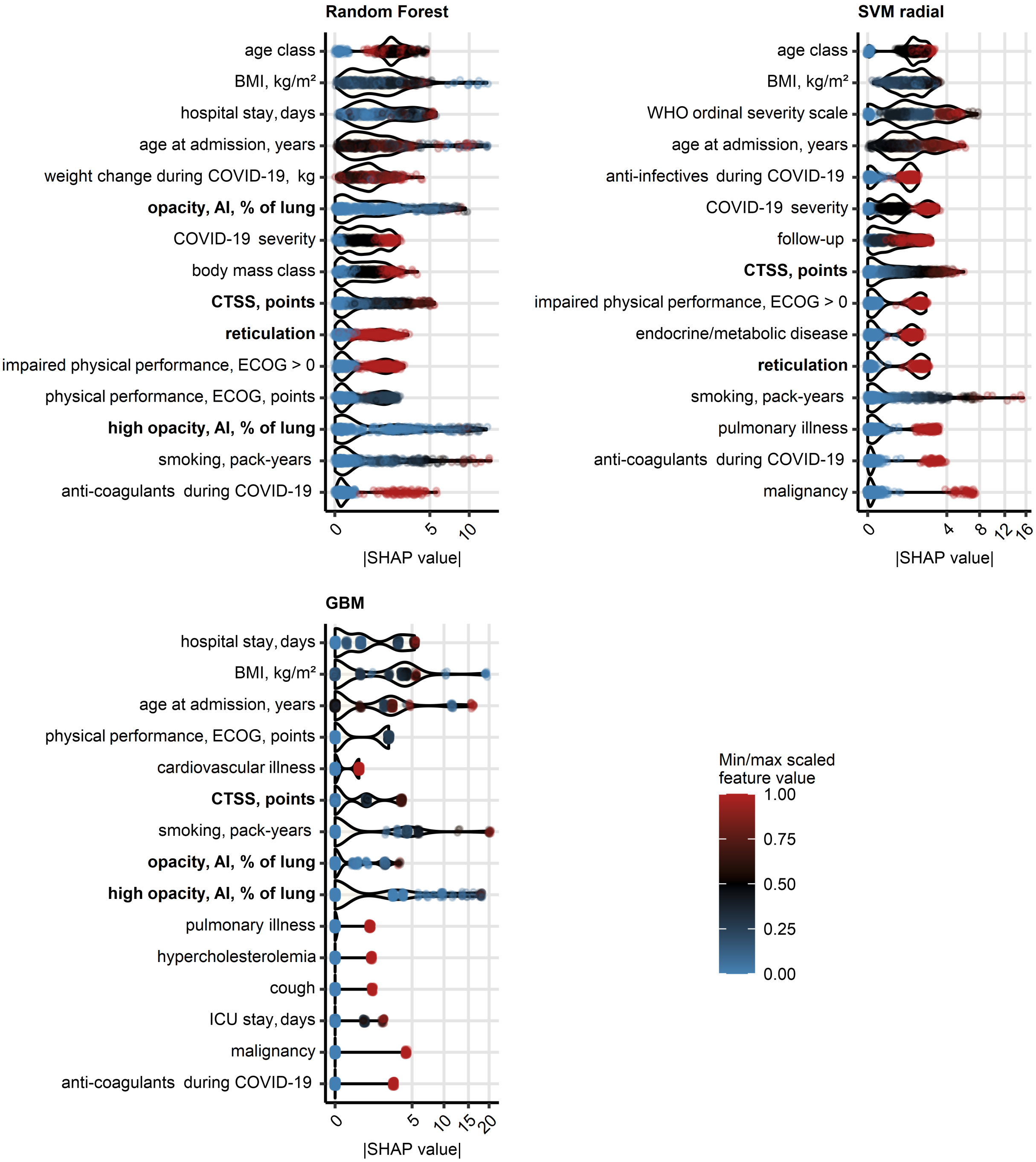

Supplementary Figure S16. Explanatory variable importance for models of diffusion capacity for carbon monoxide measured by Shapley additive explanations.

Importance of explanatory variables for the machine learning models of diffusion capacity for carbon monoxide (% of reference, Figure 2) was investigated by Shapley additive explanations (SHAP). Absolute SHAP values for explanatory variables with the 15 largest

mean SHAP values are presented in violin plots. Points represent single observations, point colors code for minimum/maximum scaled value of the explanatory variable. Explanatory variables obtained via computed tomography are highlighted with bold font in the Y axes.

CT: computed tomography; DLCO: diffusion capacity for carbon monoxide; BMI: body mass index; opacity and high opacity, AI: opacity and high opacity of the lung determined by artificial intelligence; CTSS: human-determined CT severity score, sum for all lobe; ECOG: Eastern Cooperative Oncology Group physical performance score; mMRC: modified Medical Research Council dyspnea scale; ICU: intensive care unit.

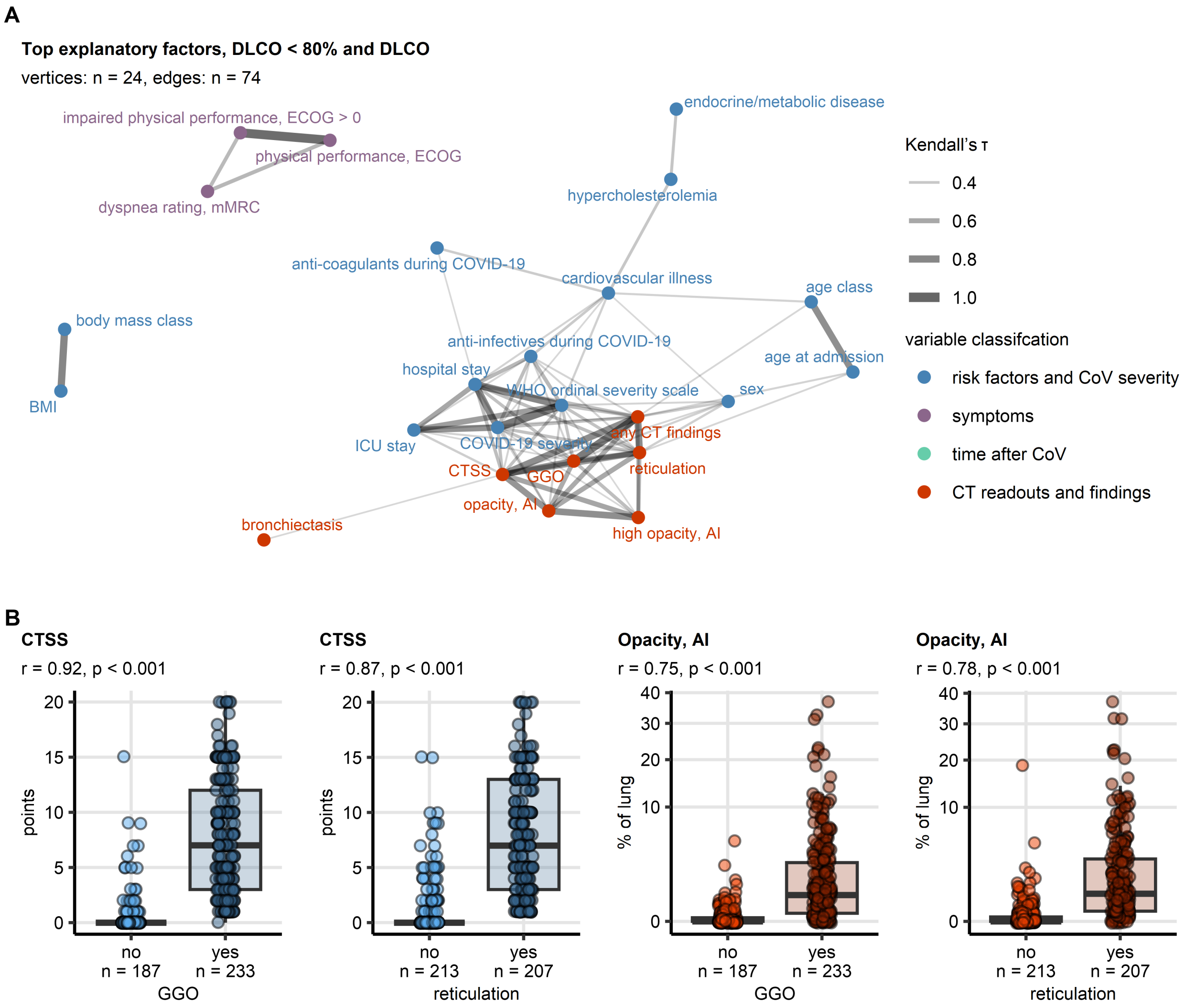

Supplementary Figure S17. Co-linearity of the most influential computed tomography-related variables for prediction of insufficiency and percentage of reference of diffusion capacity for carbon monoxide.

The most influential variables for prediction of insufficiency and percentage of reference of diffusion capacity for carbon monoxide by machine learning models were identified as features with the largest mean absolute Shapley additive explanations (SHAP) as presented in Figure 3 and Supplementary Figure S16.

(A) Weighted graph of correlations between the most influential explanatory variables. The graph edges are defined by correlations with Kendall's τ ≥ 0.3, the edge weights correspond to the τ values. Isolated vertices, i.e. explanatory variables without any correlations with τ ≥ 0.3, were removed. The graph was visualized with the Fruchterman-Reingold algorithm. The explanatory variables, i.e. vertices of the graph, are depicted as points; point color codes for

classification of the explanatory variable. Edges are represented by lines; line width and color codes for τ value.

(B) Comparison of CTSS and lung opacity between observations with and without ground glass opacity (GGO) and reticulations. Statistical significance was determined by blocked bootstrap test with r effect size statistic. Median values with interquartile ranges are depicted as boxes, whiskers span over 150% of the interquartile ranges, single observations are visualized as points. Effect sizes and p values are displayed in the plot captions. Numbers of observations are indicated in the X axes.

References

1. Sonnweber T, Sahanic S, Pizzini A, Luger A, Schwabl C, Sonnweber B, Kurz K, Koppelstätter S, Haschka D, Petzer V, et al. Cardiopulmonary recovery after COVID-19: An observational prospective multicentre trial. *European Respiratory Journal* (2021) 57: doi: 10.1183/13993003.03481-2020

2. Sonnweber T, Tymoszuk P, Sahanic S, Boehm A, Pizzini A, Luger A, Schwabl C, Nairz M, Grubwieser P, Kurz K, et al. Investigating phenotypes of pulmonary COVID-19 recovery: A longitudinal observational prospective multicenter trial. *eLife* (2022) 11: doi: 10.7554/ELIFE.72500

3. Luger AK, Sonnweber T, Gruber L, Schwabl C, Cima K, Tymoszuk P, Gerstner AK, Pizzini A, Sahanic S, Boehm A, et al. Chest CT of Lung Injury 1 Year after COVID-19 Pneumonia: The CovILD Study. *Radiology* (2022) 304:462–470. doi: 10.1148/radiol.211670

4. Sahanic S, Tymoszuk P, Luger AK, Hüfner K, Boehm A, Pizzini A, Schwabl C, Koppelstätter S, Kurz K, Asshoff M, et al. COVID-19 and its continuing burden after 12 months: a longitudinal observational prospective multicentre trial. *ERJ open research* (2023) 9:00317–2022. doi: 10.1183/23120541.00317-2022

5. Hansell DM, Bankier AA, MacMahon H, McLoud TC, Müller NL, Remy J. Fleischner Society: Glossary of terms for thoracic imaging. (2008) 246:697–722. doi: 10.1148/radiol.2462070712

6. Wickham Hadley. *ggplot2: Elegant Graphics for Data Analysis*. 1st ed. New York: Springer-Verlag (2016). https://ggplot2.tidyverse.org

7. Henry L, Wickham Hadley. rlang: Functions for Base Types and Core R and ’Tidyverse’ Features. (2022) https://cran.r-project.org/web/packages/rlang/index.html

8. Gagolewski M, Tartanus B. Package ’stringi’. (2021) https://cran.r-project.org/web/packages/stringi/index.html http://cran.ism.ac.jp/web/packages/stringi/stringi.pdf

9. Vaughan D, Dancho M, RStudio. furrr: Apply Mapping Functions in Parallel using Futures. (2022) https://cran.r-project.org/package=furrr

10. Folashade D, Microsoft Corporation, Weston S, Tenenbaum D. doParallel: Foreach Parallel Adaptor for the ’parallel’ Package. (2022) https://cran.r-project.org/web/packages/doParallel/index.html

11. Kassambara A. rstatix: Pipe-Friendly Framework for Basic Statistical Tests. (2021) https://cran.r-project.org/package=rstatix

12. Mangiafico S. rcompanion: Functions to Support Extension Education Program Evaluation. (2022) https://cran.r-project.org/package=rcompanion

13. Ripley B. MASS: Support Functions and Datasets for Venables and Ripley's MASS. (2022) https://cran.r-project.org/package=MASS

14. Csardi G, Nepusz T. The igraph software package for complex network research. *InterJournal* (2006) Complex Sy:1695. https://igraph.org

15. Kuhn M. Building predictive models in R using the caret package. *Journal of Statistical Software* (2008) 28:1–26. doi: 10.18637/jss.v028.i05

16. López-Ratón M, Rodríguez-Álvarez MX, Cadarso-Suárez C, Gude-Sampedro F. Optimalcutpoints: An R package for selecting optimal cutpoints in diagnostic tests. *Journal of Statistical Software* (2014) 61:1–36. doi: 10.18637/jss.v061.i08

17. Wright MN, Ziegler A. ranger: A Fast Implementation of Random Forests for High Dimensional Data in C++ and R. *Journal of Statistical Software* (2017) 77:1–17. doi: 10.18637/JSS.V077.I01

18. Breiman L. Random forests. *Machine Learning* (2001) 45:5–32. doi: 10.1023/A:1010933404324

19. Ripley BD. *Pattern recognition and neural networks*. Cambridge University Press (2014). doi: 10.1017/CBO9780511812651

20. Weston J, Watkins C. Multi-Class Support Vector Machines. (1998)

21. Karatzoglou A, Hornik K, Smola A, Zeileis A. kernlab - An S4 Package for Kernel Methods in R. *Journal of Statistical Software* (2004) 11:1–20. doi: 10.18637/JSS.V011.I09

22. Greenwell B, Boehmke B, Cunningham J, Developers G. gbm: Generalized Boosted Regression Models. (2022) https://cran.r-project.org/package=gbm

23. Friedman JH. Stochastic gradient boosting. *Computational Statistics & Data Analysis* (2002) 38:367–378. doi: 10.1016/S0167-9473(01)00065-2

24. Friedman JH. Greedy function approximation: A gradient boosting machine. *https://doiorg/101214/aos/1013203451* (2001) 29:1189–1232. doi: 10.1214/AOS/1013203451

25. Covert I, Lee SI. Improving KernelSHAP: Practical Shapley Value Estimation via Linear Regression. *Proceedings of Machine Learning Research* (2020) 130:3457–3465. https://arxiv.org/abs/2012.01536v3

26. Lundberg SM, Lee SI. A Unified Approach to Interpreting Model Predictions. *Advances in Neural Information Processing Systems* (2017) 2017-Decem:4766–4775. https://arxiv.org/abs/1705.07874v2

27. Mayer M, Stando A. shapviz: SHAP Visualizations. (2023) https://cran.r-project.org/web/packages/shapviz/index.html

28. Zeileis A, Grothendieck G, Ryan JA, Ulrich JM, Andrews F. zoo: S3 Infrastructure for Regular and Irregular Time Series (Z's Ordered Observations). (2022) https://cran.r-project.org/package=zoo

29. Yan L. ggvenn: Draw Venn Diagram by 'ggplot2'. (2021) https://cran.r-project.org/package=ggvenn

30. Sachs MC. Plotroc: A tool for plotting ROC curves. *Journal of Statistical Software* (2017) 79:1–19. doi: 10.18637/jss.v079.c02

31. Mayer M, Watson D, Biecek P. kernelshap: Kernel SHAP. (2023) https://cran.r-project.org/web/packages/kernelshap/index.html

32. Briatte F, Bojanowski M, Canouil M, Charlop-Powers Z, Fisher JC, Johnson K, Rinker T. ggnetwork: Geometries to Plot Networks with 'ggplot2'. (2021) https://cran.r-project.org/package=ggnetwork

33. Gohel D. flextable: Functions for Tabular Reporting. (2022) https://cran.r-project.org/web/packages/flextable/index.html

34. Wilke CO. *Fundamentals of Data Visualization: A Primer on Making Informative and Compelling Figures*. 1st ed. Sebastopol: O'Reilly Media (2019).

35. Allaire J, Xie Y, McPherson J, Luraschi J, Ushey K, Atkins A, Wickham H, Cheng J. rmarkdown: Dynamic Documents for R. (2022) https://cran.r-project.org/web/packages/rmarkdown/index.html

36. Xie Y. *Bookdown: Authoring books and technical documents with R Markdown*. (2016). doi: 10.1201/9781315204963

37. Benjamini Y, Hochberg Y. Controlling the False Discovery Rate: A Practical and Powerful Approach to Multiple Testing. *Journal of the Royal Statistical Society: Series B (Methodological)* (1995) 57:289–300. doi: 10.1111/j.2517-6161.1995.tb02031.x

38. McHugh ML. Interrater reliability: the kappa statistic. *Biochemia Medica* (2012) 22:276. doi: 10.11613/bm.2012.031

39. Cohen J. Statistical Power Analysis for the Behavioral Sciences. *Statistical Power Analysis for the Behavioral Sciences* (2013) doi: 10.4324/9780203771587

40. Cohen J. A Coefficient of Agreement for Nominal Scales. *Educational and Psychological Measurement* (1960) 20:37–46. doi: 10.1177/001316446002000104

41. Sharifi H, Lai YK, Guo H, Hoppenfeld M, Guenther ZD, Johnston L, Brondstetter T, Chhatwani L, Nicolls MR, Hsu JL. Machine Learning Algorithms to Differentiate Among Pulmonary Complications After Hematopoietic Cell Transplant. *Chest* (2020) 158:1090. doi: 10.1016/J.CHEST.2020.02.076

42. Murdaca G, Caprioli S, Tonacci A, Billeci L, Greco M, Negrini S, Cittadini G, Zentilin P, Spagnolo EV, Gangemi S. A Machine Learning Application to Predict Early Lung Involvement in Scleroderma: A Feasibility Evaluation. *Diagnostics* (2021) 11:1880. doi: 10.3390/DIAGNOSTICS11101880

43. Nikolaou V, Massaro S, Garn W, Fakhimi M, Stergioulas L, Price DB. Fast decliner phenotype of chronic obstructive pulmonary disease (COPD): applying machine learning for predicting lung function loss. *BMJ Open Respiratory Research* (2021) 8: doi: 10.1136/BMJRESP-2021-000980

44. Brier GW. VERIFICATION OF FORECASTS EXPRESSED IN TERMS OF PROBABILITY. *Monthly Weather Review* (1950) 78:1–3. doi: 10.1175/1520-0493(1950)078<0001:vofeit>2.0.co;2

45. Goldstein-Greenwood J. A Brief on Brier Scores | UVA Library. (2021) https://library.virginia.edu/data/articles/a-brief-on-brier-scores [Accessed September 5, 2023]

46. Viering T, Loog M. The Shape of Learning Curves: A Review. *IEEE transactions on pattern analysis and machine intelligence* (2023) 45:7799–7819. doi: 10.1109/TPAMI.2022.3220744

47. Zhang B, Horvath S. A general framework for weighted gene co-expression network analysis. *Statistical Applications in Genetics and Molecular Biology* (2005) 4: doi: 10.2202/1544-6115.1128/MACHINEREADABLECITATION/RIS
