# Supplementary figures and images for: Machine Learning Based Multi-Parameter Modeling for Prediction of Post-Inflammatory Lung Changes
Source: Diagnostics (Basel). 2025 Mar 20;15(6):783. doi: 10.3390/diagnostics15060783 (PMC11941013; doi:10.3390/diagnostics15060783)

# FVC

n = 420

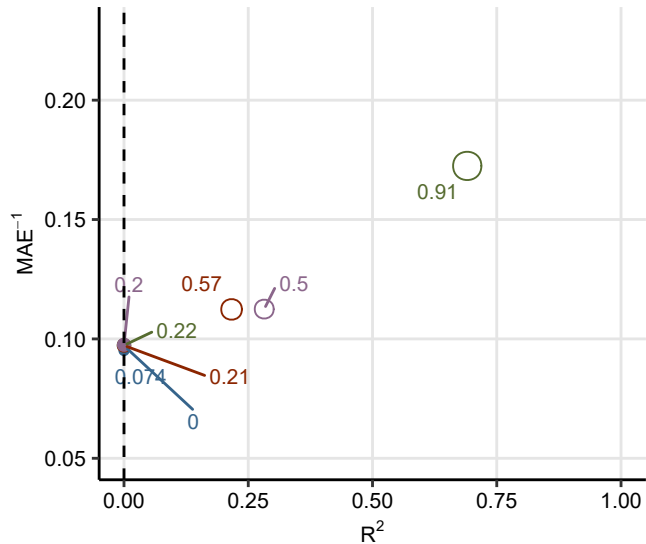

# FEV1

n = 420

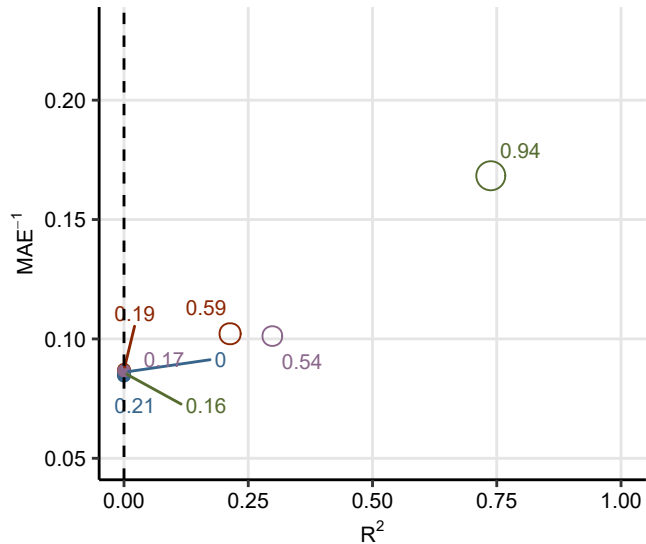

● GBM ● Neural network ● Random Forest ● SVM radial

Supplement: Supplementary file 1 [file diagnostics-15-00783-s001.zip › figure_s12_performance_fvc_fev_regression.pdf]

**A****DLCO < 80%, GBM predictions**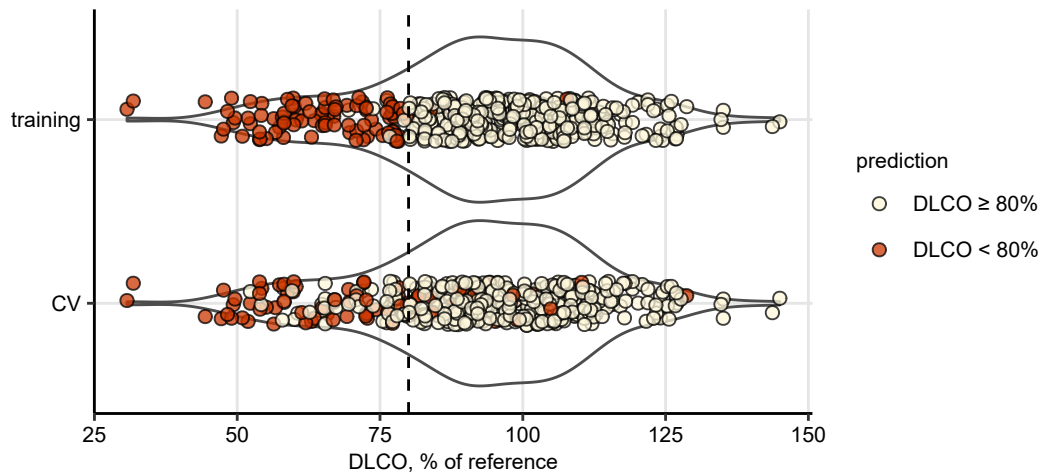**B****DLCO < 80%, GBM prediction accuracy**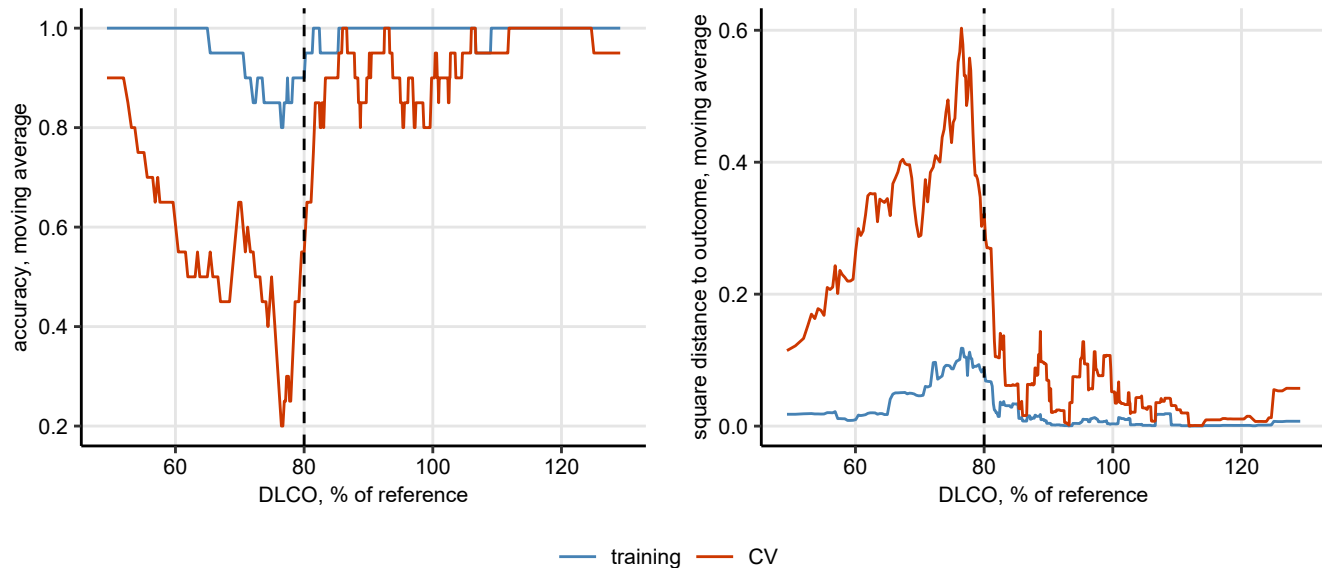

Supplement: Supplementary file 1 [file diagnostics-15-00783-s001.zip › figure_s14_reduced_dlco_predictions_dlco_percentage.pdf]

**A**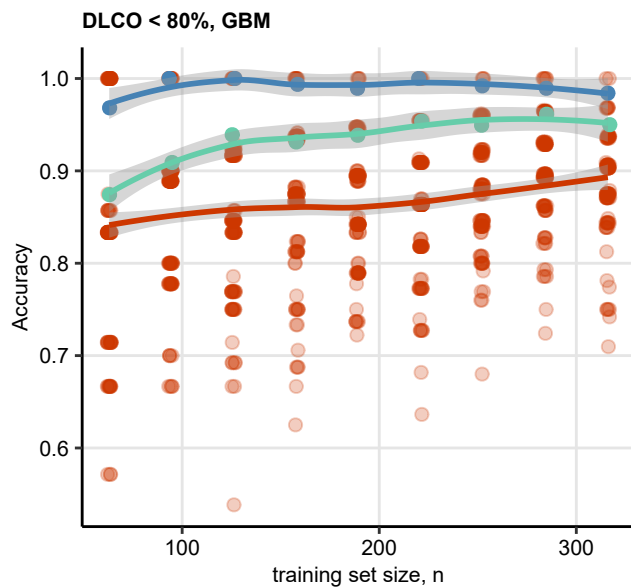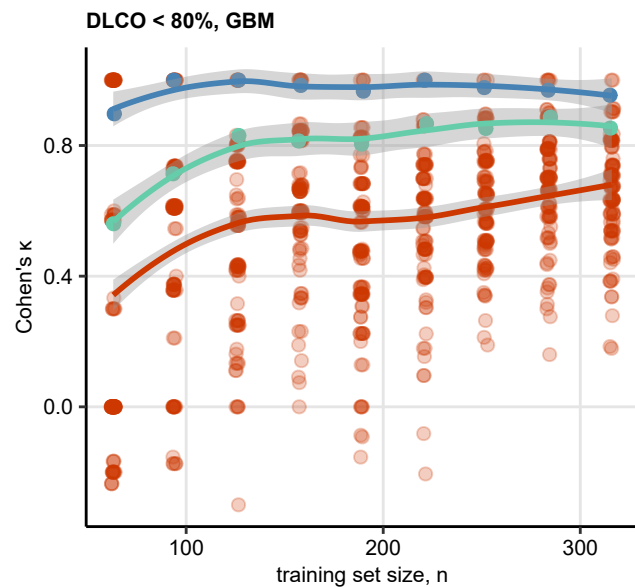**B**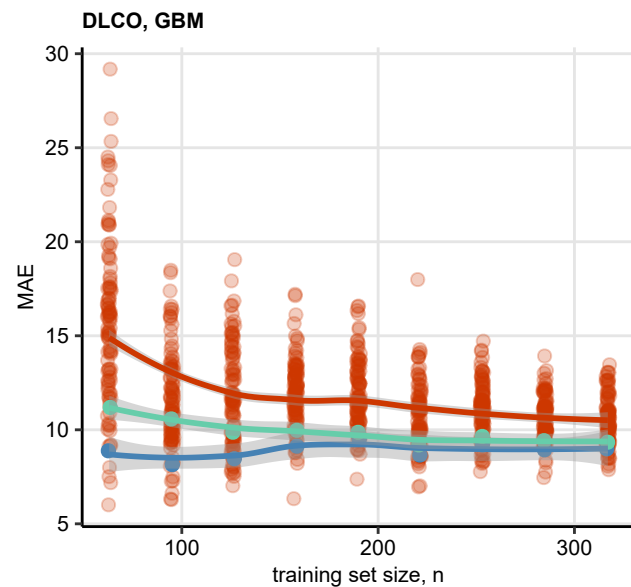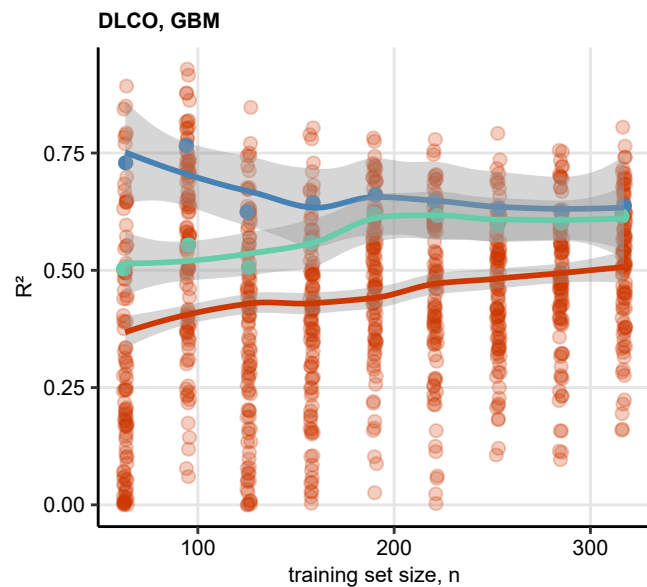

● training subset    ● test subset    ● cross-validation

Supplement: Supplementary file 1 [file diagnostics-15-00783-s001.zip › figure_s15_learning_curves_DLCO_models.pdf]

Random Forest

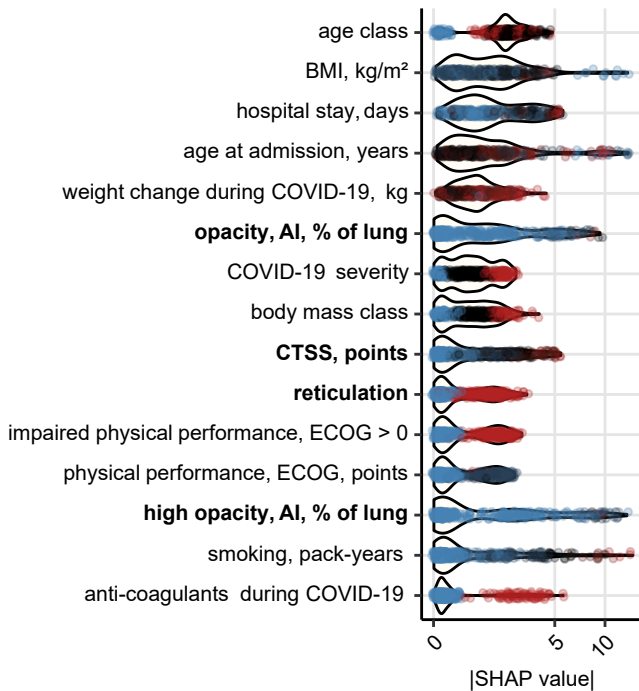

SVM radial

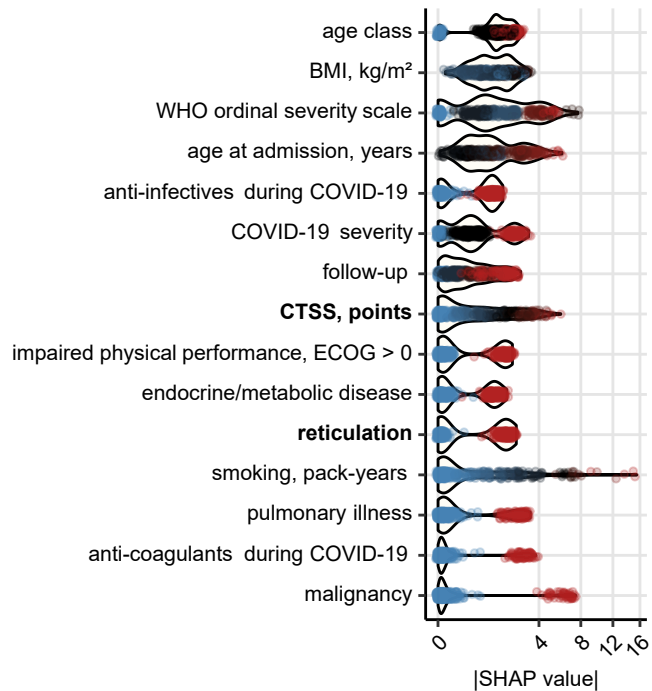

GBM

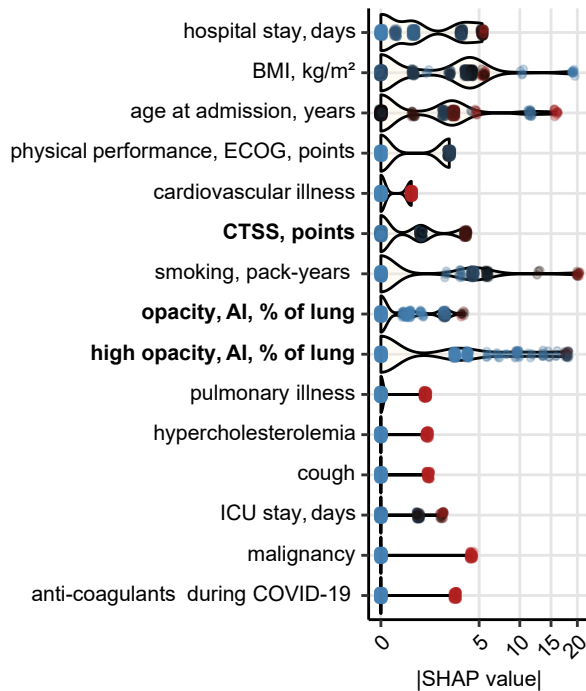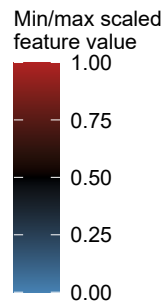

Supplement: Supplementary file 1 [file diagnostics-15-00783-s001.zip › figure_s16_dlco_shap_importance.pdf]

GGO

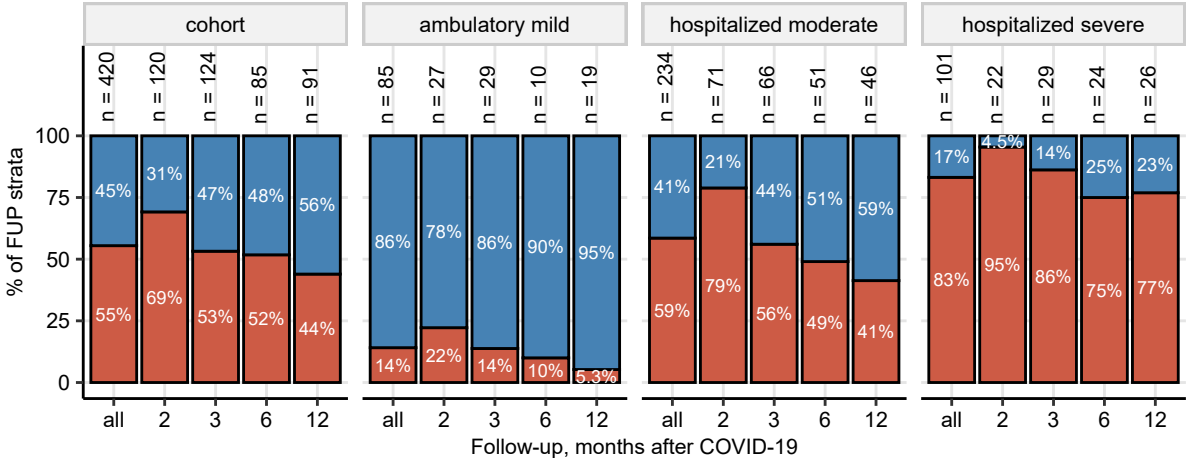

Reticulation

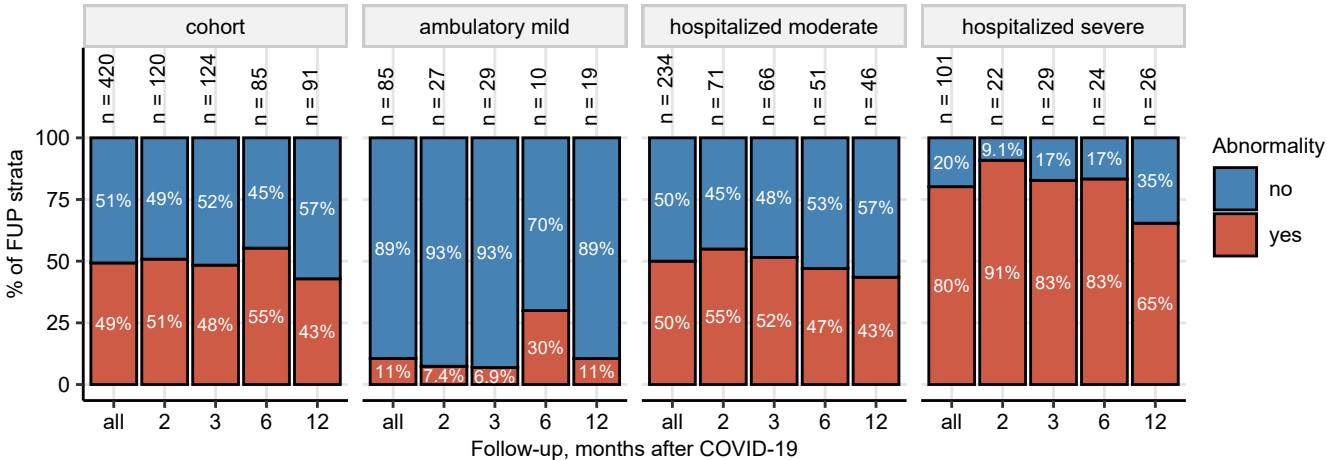

Consolidation

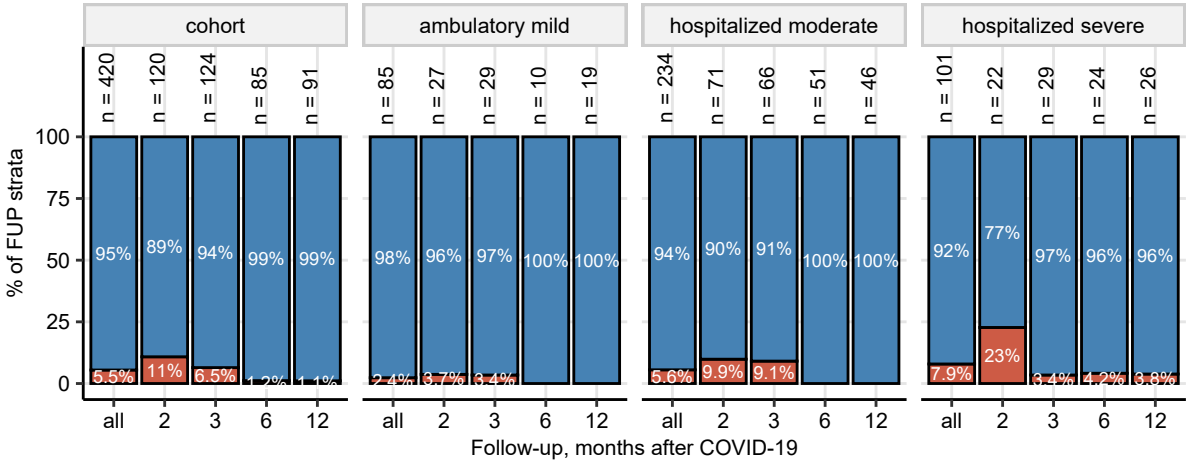

Supplement: Supplementary file 1 [file diagnostics-15-00783-s001.zip › figure_s1_ct_abnormality_time_course.pdf]

## CTSS

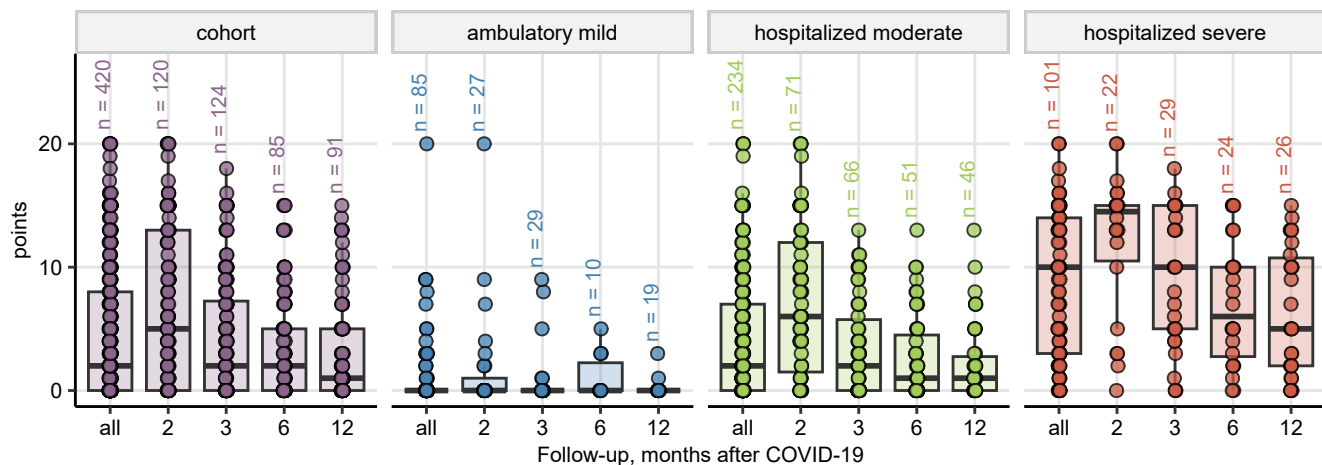

## Opacity, AI

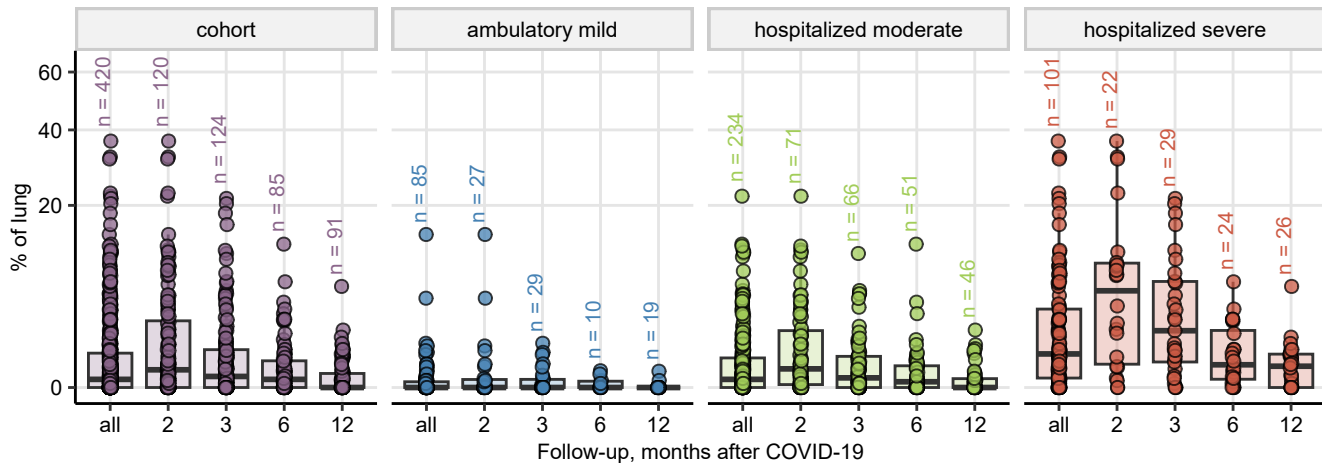

## High opacity, AI

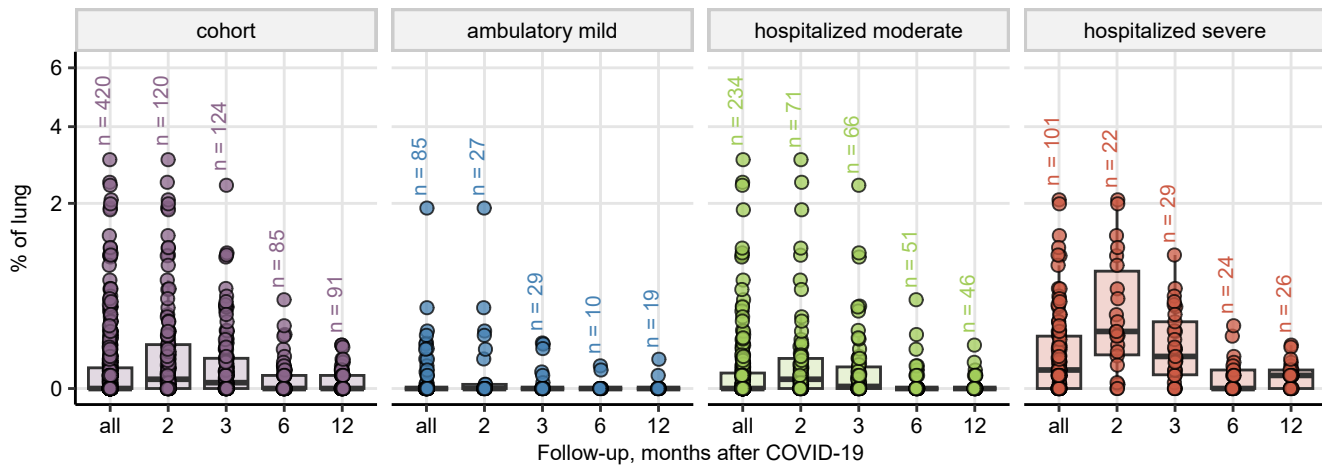

Supplement: Supplementary file 1 [file diagnostics-15-00783-s001.zip › figure_s2_ct_paramater_time_course.pdf]

**DLCO < 80%**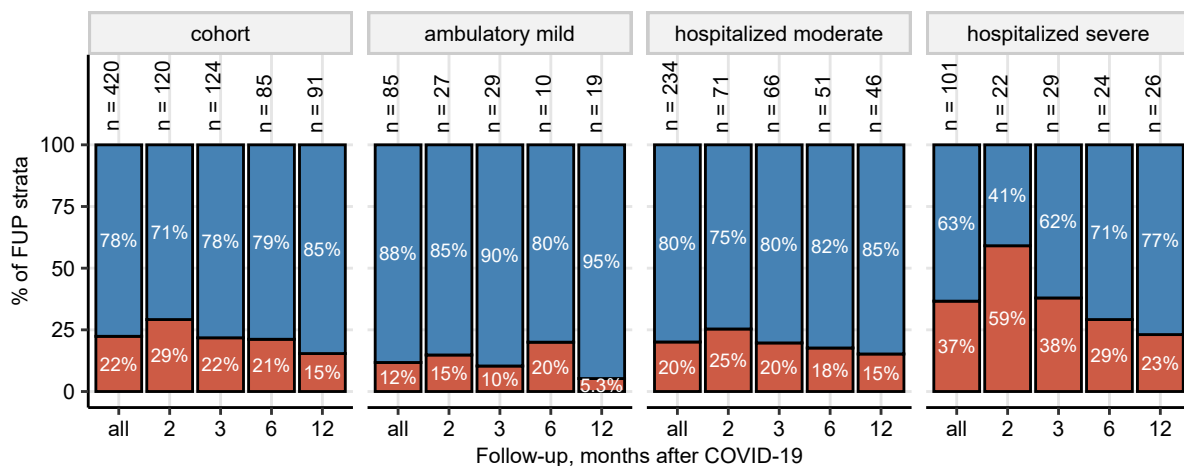**FVC < 80%**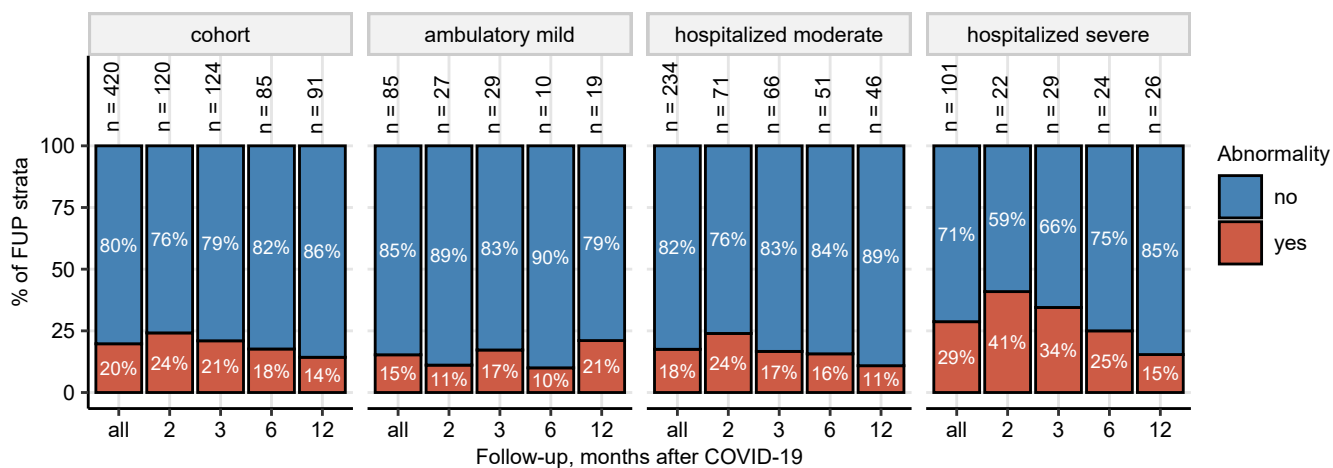**FEV1 < 80%**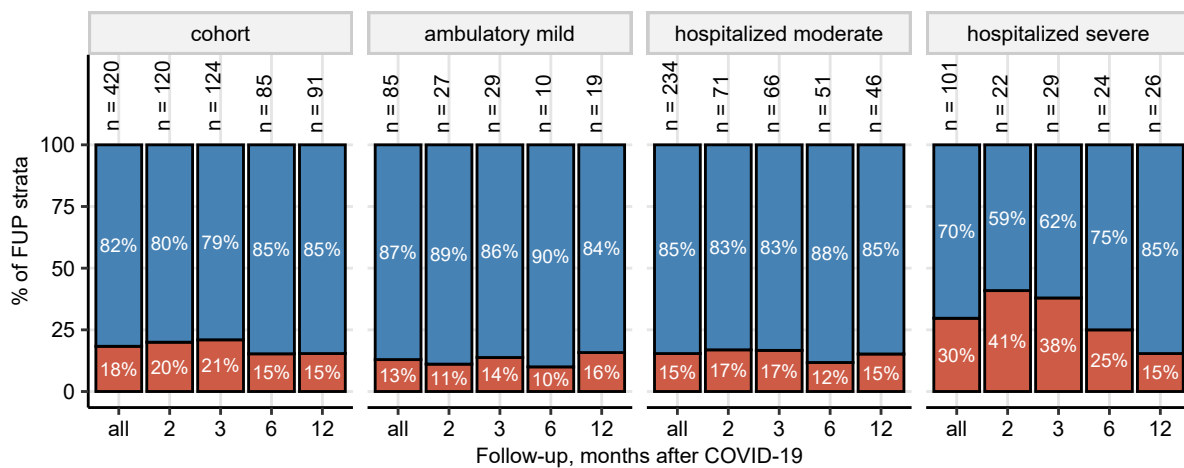

Supplement: Supplementary file 1 [file diagnostics-15-00783-s001.zip › figure_s4_lft_abnormality_time_course.pdf]

**DLCO**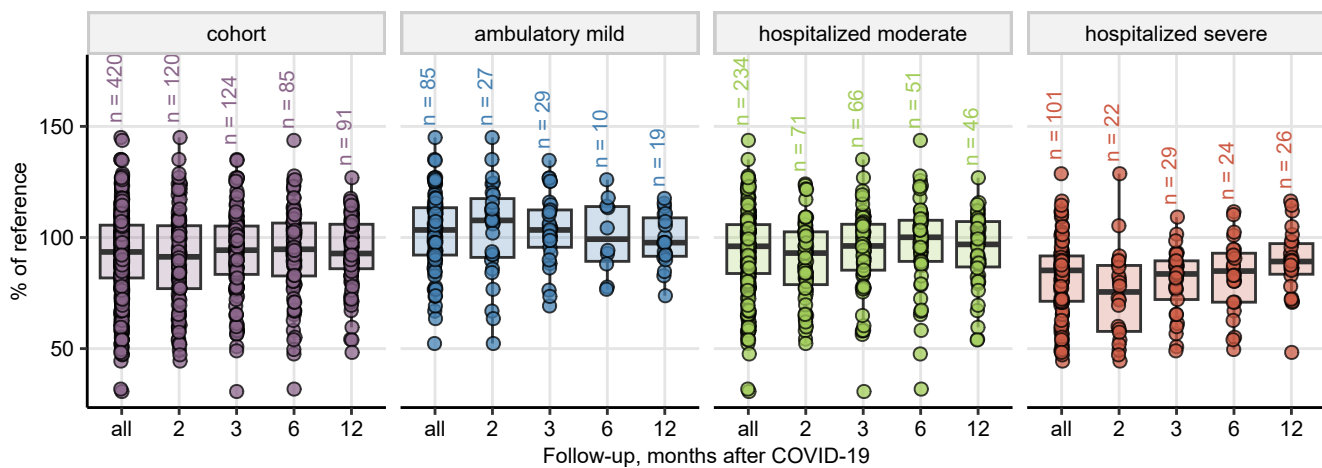**FVC**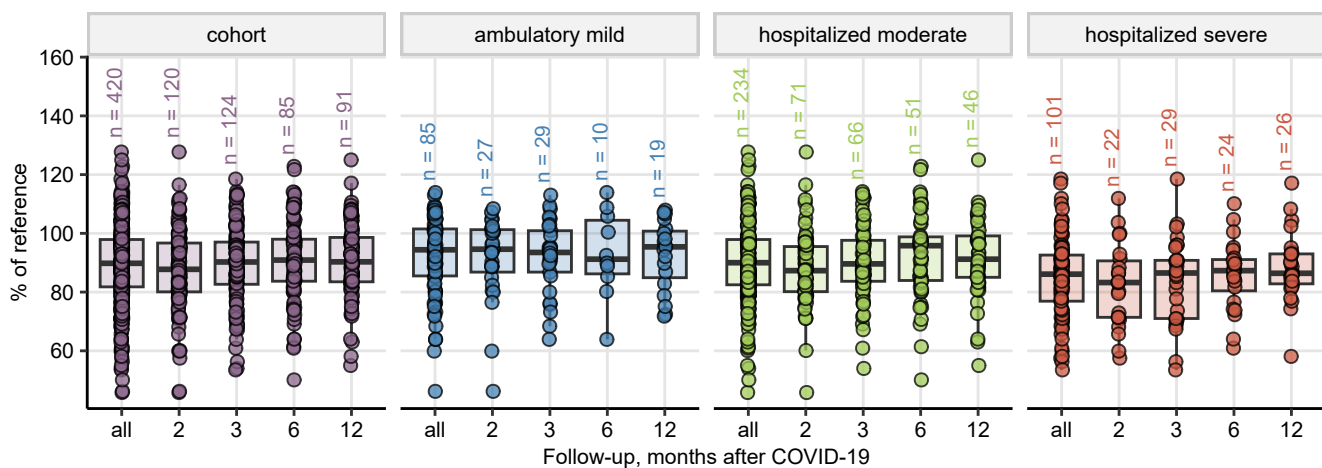**FEV1**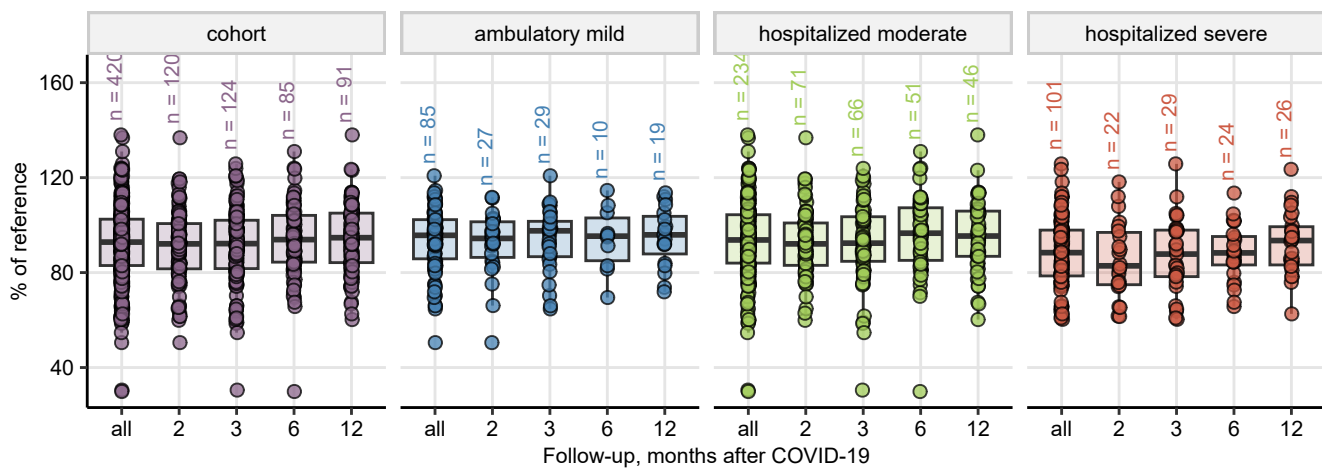

Supplement: Supplementary file 1 [file diagnostics-15-00783-s001.zip › figure_s5_lft_parameter_time_course.pdf]

## Dyspnea, mMRC > 0

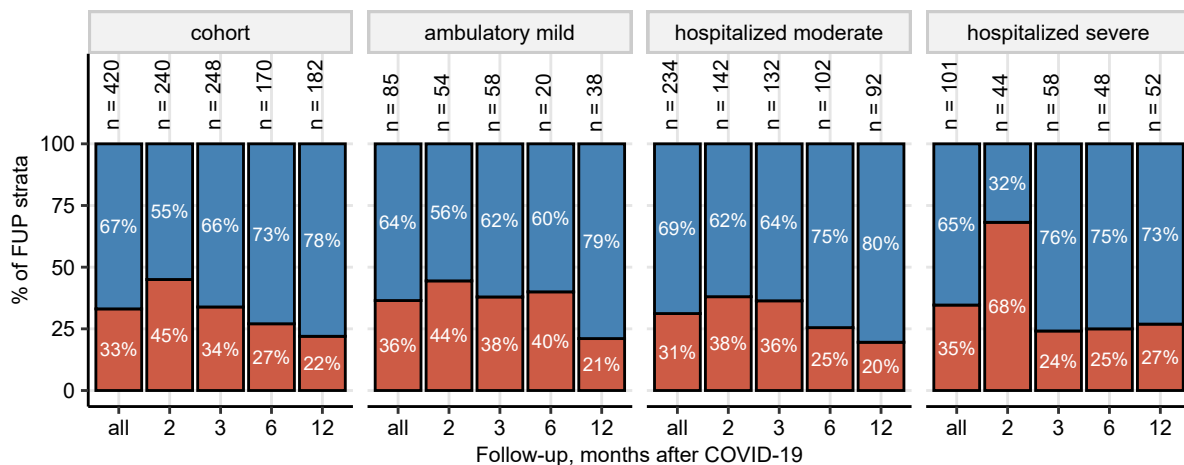

## Cough

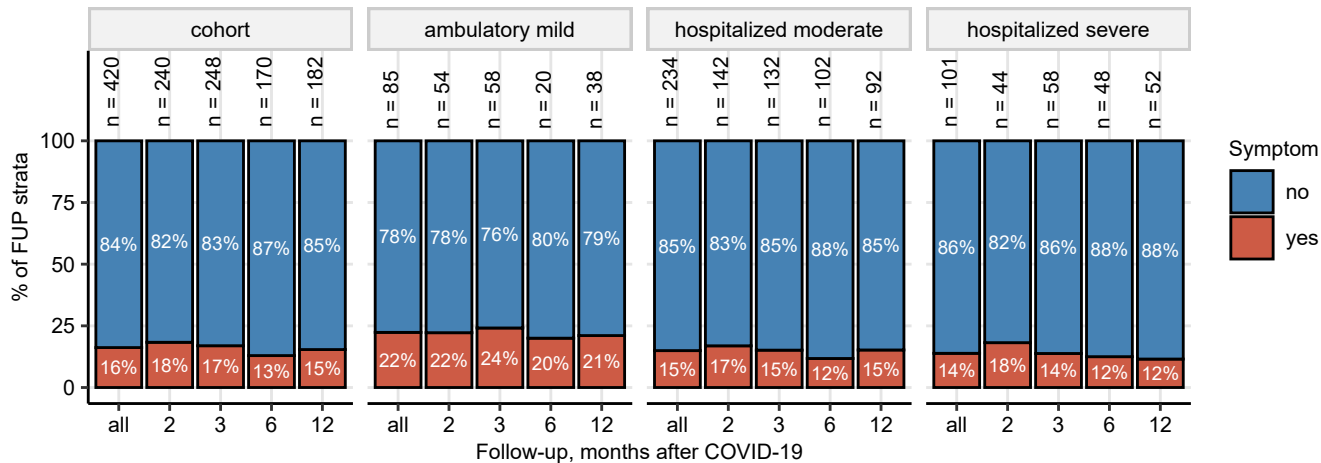

## Impaired physical performance, ECOG > 0

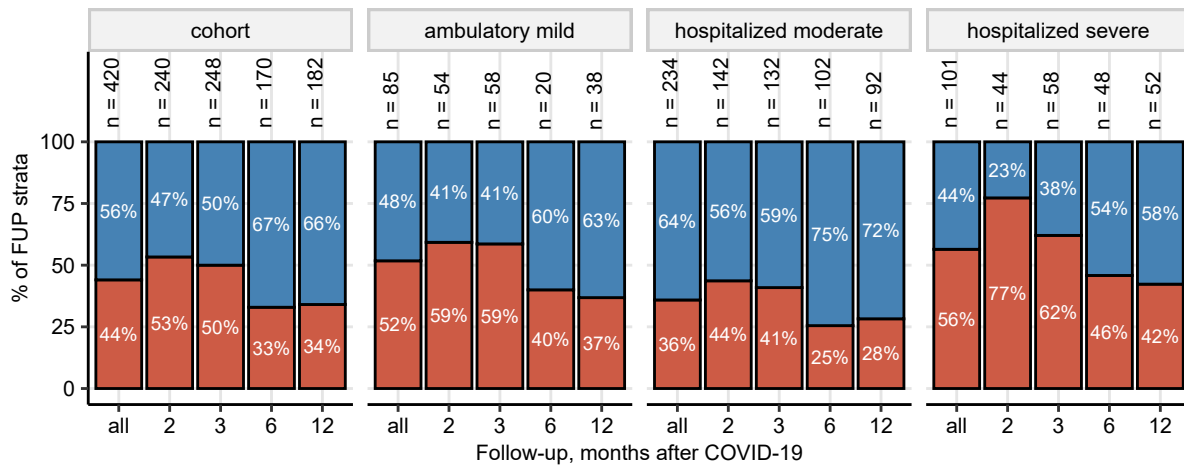

Supplement: Supplementary file 1 [file diagnostics-15-00783-s001.zip › figure_s7_symptom_time_course.pdf]

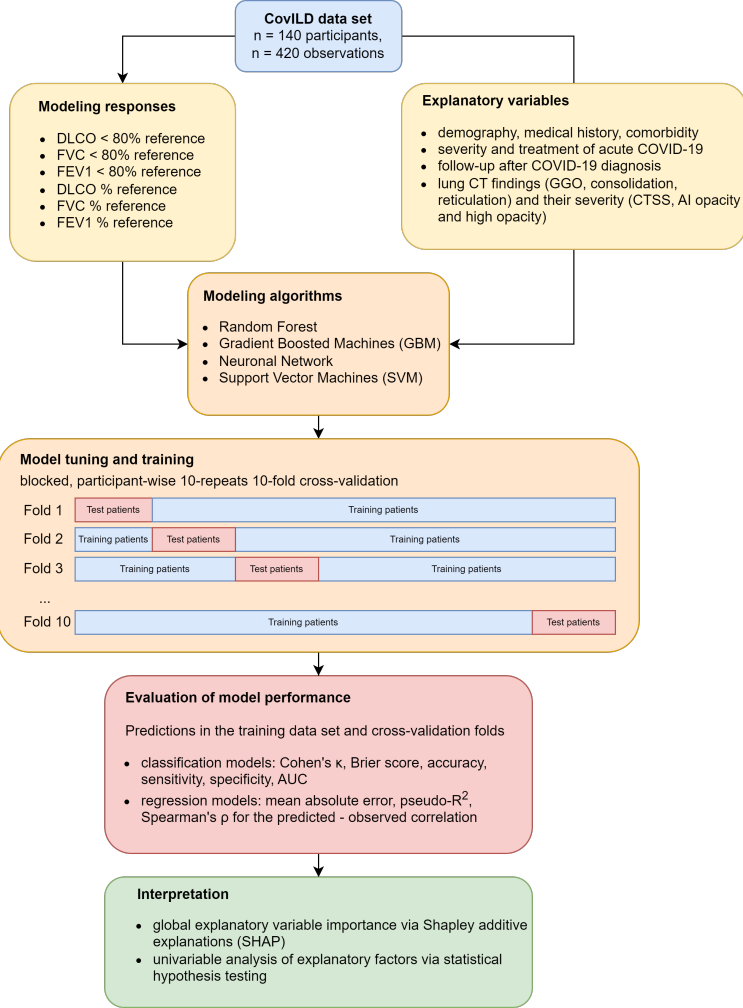

Supplement: Supplementary file 1 [file diagnostics-15-00783-s001.zip › figure_s8_modeling_strategy.pdf]
